# *PRM1* and *KAR5* function in cell-cell fusion and karyogamy to drive distinct bisexual and unisexual cycles in the *Cryptococcus* pathogenic species complex

**DOI:** 10.1371/journal.pgen.1007113

**Published:** 2017-11-27

**Authors:** Ci Fu, Joseph Heitman

**Affiliations:** Department of Molecular Genetics and Microbiology, Duke University Medical Center, Durham, NC, United States of America; University of California, UNITED STATES

## Abstract

Sexual reproduction is critical for successful evolution of eukaryotic organisms in adaptation to changing environments. In the opportunistic human fungal pathogens, the *Cryptococcus* pathogenic species complex, *C*. *neoformans* primarily undergoes bisexual reproduction, while *C*. *deneoformans* undergoes both unisexual and bisexual reproduction. During both unisexual and bisexual cycles, a common set of genetic circuits regulates a yeast-to-hyphal morphological transition, that produces either monokaryotic or dikaryotic hyphae. As such, both the unisexual and bisexual cycles can generate genotypic and phenotypic diversity *de novo*. Despite the similarities between these two cycles, genetic and morphological differences exist, such as the absence of an opposite mating-type partner and monokaryotic instead of dikaryotic hyphae during *C*. *deneoformans* unisexual cycle. To better understand the similarities and differences between these modes of sexual reproduction, we focused on two cellular processes involved in sexual reproduction: cell-cell fusion and karyogamy. We identified orthologs of the plasma membrane fusion protein Prm1 and the nuclear membrane fusion protein Kar5 in both *Cryptococcus* species, and demonstrated their conserved roles in cell fusion and karyogamy during *C*. *deneoformans* α-α unisexual reproduction and *C*. *deneoformans* and *C*. *neoformans* **a**-α bisexual reproduction. Notably, karyogamy occurs inside the basidum during bisexual reproduction in *C*. *neoformans*, but often occurs earlier following cell fusion during bisexual reproduction in *C*. *deneoformans*. Characterization of these two genes also showed that cell fusion is dispensable for solo unisexual reproduction in *C*. *deneoformans*. The blastospores produced along hyphae during *C*. *deneoformans* unisexual reproduction are diploid, suggesting that diploidization occurs early during hyphal development, possibly through either an endoreplication pathway or cell fusion-independent karyogamy events. Taken together, our findings suggest distinct mating mechanisms for unisexual and bisexual reproduction in *Cryptococcus*, exemplifying distinct evolutionary trajectories within this pathogenic species complex.

## Author summary

Sexuality is ubiquitous in eukaryotic systems, but it is present in diverse forms, ranging from distinct sexual individuals to parthenogenic organisms in both animals and plants. Consequently, different organisms have evolved different reproduction strategies in which cell-cell fusion and nuclear fusion (karyogamy) play fundamental roles. The opportunistic human fungal pathogen *Cryptococcus neoformans* can undergo both bisexual reproduction between **a** and α cells and selfing unisexual reproduction, which offsets the cost of finding a mating partner, coinciding with the observation that 99% of clinical and environmental isolates are mating type α. It has been a central interest to elucidate the similarities and differences between these two sexual cycles. Here, we identified and characterized two genes in the *Cryptococcus* species complex, *PRM1* and *KAR5*, which play conserved roles in plasma membrane fusion and karyogamy in fungi. We showed that unisexual reproduction is largely independent from cell-cell fusion and is mechanistically different from bisexual reproduction. We also demonstrated that karyogamy takes place at different stages during bisexual reproduction between two sister species, exemplifying distinct evolutionary trajectories within the pathogenic species complex.

## Introduction

Sexual reproduction is ubiquitous in eukaryotic systems and promotes genetic diversity important for successful evolutionary adaptation to ever-changing environments [1]. In addition to bisexual reproduction between mating partners of opposite sexes, many eukaryotic systems, including fish, amphibians, and reptiles, can undergo unisexual reproduction, termed parthenogenesis, often in the absence of the opposite sex [2]. During bisexual reproduction, parental gametes undergo cell fusion and nuclear fusion to produce recombinant progeny, whereas during parthenogenesis, the maternal genome undergoes reduplication through either cell-cell fusion or endoreplication to produce clonal offspring of the mother [2]. Analogous to parthenogenesis, several human fungal pathogens have been reported to undergo both unisexual and bisexual reproduction [3, 4]. In *Candida albicans* bisexual reproduction, **a**/**a** and α/α cells first undergo white-opaque switching to become mating competent and then form tetraploid cells via cell fusion and nuclear fusion. These cells then undergo a parasexual cycle to return to the diploid state. During *C*. *albicans* unisexual reproduction, loss of the Bar1 protease in **a**/**a** cells enables auto-response to *MF*α pheromone and promotes cell and nuclear fusion producing tetraploid cells [5]. During bisexual reproduction in the *Cryptococcus* species complex, cell fusion triggers a dramatic yeast-hyphal morphological transition, producing dikaryotic hyphae. The growing tips of these hyphae differentiate into basidia, in which two nuclei undergo nuclear fusion to produce basidiospores through meiosis [6]. During the unisexual cycle, α or **a** cells initiate hyphal growth and form monokaryotic hyphae, during which the haploid nucleus undergoes a ploidy increase through either cell-cell fusion followed by nuclear fusion, nuclear fusion between mother and daughter cells, or an endoreplication pathway, and the diploid nucleus inside the basidium then undergoes meiosis and produces haploid spore progeny [7, 8].

Sexual reproduction has only been observed under laboratory conditions In the *Cryptococcus* species complex. However, spore-like cells have been harvested from the environment, suggesting the sexual cycle may occur in natural environments [9, 10]. Unisexual reproduction has been documented for *C*. *neoformans*, *C*. *deneoformans*, and *C*. *gattii* [7, 11, 12]. Based on evidence from population genetics studies, natural isolates also recombine through unisexual reproduction, which may be of ecological significance because more than 99% of environmental and clinical isolates are the α mating type [13–16]. Of note, the unisexual cycle generates genotypic and phenotypic diversity *de novo*, similar to the bisexual cycle [17]. A common set of genetic circuits govern both unisexual and bisexual reproduction, [8, 18–20] and both sexual cycles involve similar meiotic recombination mechanisms [21]. The recombining nature of the unisexual cycle can enable a clonal population to reverse Muller’s ratchet and avoid an evolutionary dead end [22].

Despite similar regulatory genetic circuits, fundamental differences are obvious between the two modes of sexual reproduction [23–25]. Genetically, the unisexual cycle is initiated in the absence of an opposite-mating type partner, whereas the bisexual cycle is initiated upon **a**-α cell-cell fusion. Morphologically, the unisexual cycle produces monokaryotic hyphae with unfused clamp cells, while the bisexual cycle produces dikaryotic hyphae with fused clamp cells, which allow a nucleus to migrate between adjacent hyphal compartments to maintain dikaryotic hyphae [24, 25]. While diploidization is achieved through karyogamy in the bisexual cycle, it is not yet clear how diploidization is achieved during the unisexual cycle. Three hypotheses have been proposed, including 1) cell fusion followed by karyogamy; 2) karyogamy between mitotically dividing mother-daughter cells followed by either mis-segregation of the nucleus or cytokinesis arrest; and 3) endoreplication during hyphal growth [25, 26].

In all bisexually reproducing organisms, gamete fusion is a fundamental process requiring a set of dedicated fusion proteins [27]. In the fungal kingdom, Prm1 (Pheromone regulated multi-spanning membrane protein 1) is a conserved plasma membrane protein required for plasma membrane fusion during cell-cell fusion [28–30]. In *Saccharomyces cerevisiae* and *Neurospora crassa*, deletion of *PRM1* reduces fusion frequency by approximately half and leads to cell lysis. The mutant phenotype is alleviated in the presence of a high calcium and exacerbated upon calcium depletion [31, 32]. Prm1 is also required for asexual hyphal fusion in *N*. *crassa* [29]. In *Schizosaccharomyces pombe*, deletion of *PRM1* causes a 95% reduction in cell fusion frequency independent of extracellular calcium concentration, but does not lead to a cell lysis phenotype [30].

Cell fusion has been well studied in *Cryptococcus* sexual cycles. During bisexual reproduction, **a**-α cell-cell fusion is required for hyphae induction and clamp cell-hyphal fusion is required for proper nuclear migration between adjacent hyphal compartments to maintain dikaryotic hyphal growth [6, 33]. During unisexual reproduction, α-α cell-cell fusion occurs at a low frequency whereas the presence of **a** cells can enhance α-α cell fusion ~1000 fold in a *ménage à trois* fashion [7]. G proteins in the pheromone response pathway are required for cell-cell fusion [34], and the master transcription factor Mat2 governs the yeast-hyphal morphological transition [18]. An evolutionarily conserved Ire1 kinase/endoribonuclease in the unfolded protein response pathway has been shown to negatively regulate the pheromone response pathway and is required for cell-cell fusion [35]. However, genes that are directly involved in plasma membrane fusion during cell-cell fusion have not been identified. A transcriptomic study showed that expression of the *S*. *cerevisiae PRM1* homolog in *C*. *deneoformans* is highly upregulated during hyphal growth, suggesting it may function in the sexual cycle, but its involvement in cell-cell fusion had yet to be determined [18].

Karyogamy is an essential step for intermixing of parental genetic information during sexual reproduction. Two sets of genes regulate karyogamy in *S*. *cerevisiae*. The class I genes, including *KAR1*, *KAR3*, *KAR4*, and *KAR9*, regulate nuclear congression, while the class II genes, including *KAR2*, *KAR5*, *KAR7*, *KAR8*, and *PRM3*, mediate inner and outer nuclear membrane fusion [36, 37]. Lee and Heitman identified the *Cryptococcus* karyogamy genes *KAR2*, *KAR3*, *KAR4*, *KAR7*, and *KAR8* based on homology to *S*. *cerevisiae* [38]. While homologs of *KAR2* and *KAR7* were identified in *Cryptococcus* with roles in filamentation and meiosis, respectively, homologs of *KAR3*, *KAR4*, and *KAR8* did not show karyogamy defects during unisexual or bisexual reproduction. This suggests that these genes are either rewired in *Cryptococcus* compared with *S*. *cerevisiae* or are functionally redundant in regulating nuclear fusion. *KAR2*, an ER-resident chaperone protein, is essential in *Cryptococcus*, and its overexpression partially rescues the filamentation defect of the *ire1* mutant [35, 39]. *KAR7* maintains a conserved role in mediating nuclear membrane fusion during both *Cryptococcus* unisexual and bisexual reproduction. However, a diploid strain without *KAR7* produced hyphae and basidia but failed to undergo sporulation, suggesting *KAR7* may play additional roles in meiotic processes. In *S*. *cerevisiae*, Kar5 localizes to both inner and outer nuclear membranes at the spindle pole body, and coordinates the outer and inner nuclear membrane, facilitating the inner nuclear membrane fusion step during karyogamy [40–42]. However, a *KAR5* homolog was not identified in *Cryptococcus*. A study on the *Chlamydomonas* nuclear fusion gene *GEX1* by Ning and colleagues [43] showed that protist and plant *GEX1* genes and fungal *KAR5* genes belong to an ancient cysteine rich domain (CRD) containing protein family that is conserved throughout eukaryotes, suggesting that they may share a conserved role in nuclear membrane fusion. In that same study, a *KAR5* ortholog was identified for a basidiomycetous fungus, *Puccinia graminis* [43].

In this study, we identified *PRM1* and *KAR5* orthologs in both *C*. *neoformans* and *C*. *deneoformans* and investigated their conserved functions in mediating plasma membrane and nuclear membrane fusion. Utilizing these two genes, we studied cell fusion and nuclear fusion in the *C*. *neoformans* bisexual cycle and the *C*. *deneoformans* unisexual and bisexual cycles. *C*. *neoformans* and *C*. *deneoformans* bisexual cycles were dependent on cell and nuclear fusion at different stages during sexual development, whereas, cell fusion was largely dispensable in the solo unisexual cycle of *C*. *deneoformans* and the ploidy duplication during unisexual reproduction is dependent on either endoreplication or cell fusion-independent karyogamy events. Our results provide mechanistic insights relevant to studies of mating mechanisms of unisexual reproduction and parthenogenesis in other eukaryotic systems.

## Results

### Identification of *PRM1* and *KAR5* in *Cryptococcus*

**Fig 1:**
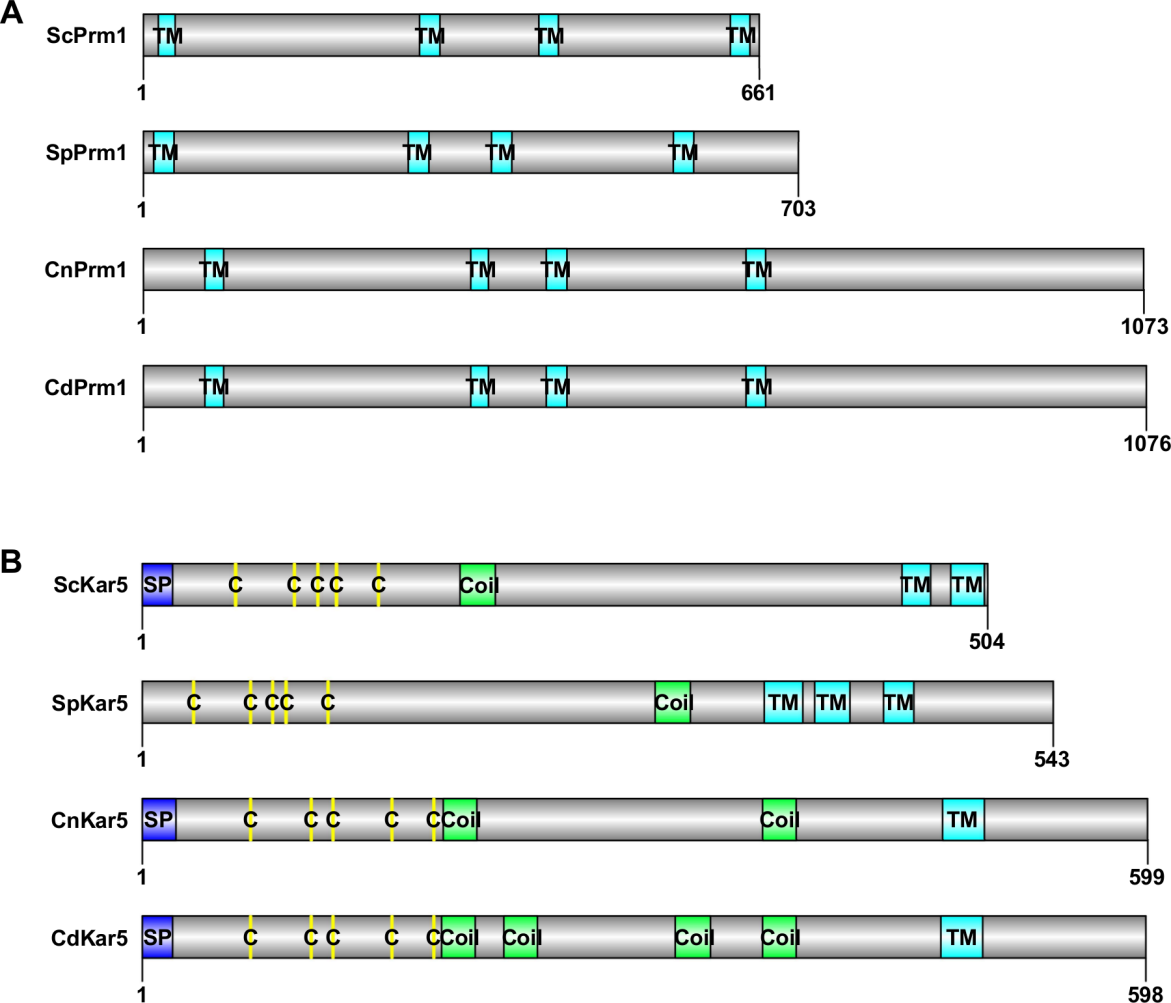
Schematic diagrams of *Cryptococcus* Prm1 and Kar5 proteins. **(A)** The Prm1 proteins from *S*. *cerevisiae*, *S*. *pombe*, *C*. *neoformans*, and *C*. *deneoformans* are drawn to scale. The four Prm1 proteins contain four transmembrane domains (TM) indicated by cyan boxes. In contrast to ScPrm1 and SpPrm1, CnPrm1 and CdPrm1 have a long C-terminal tail following the last transmembrane domain. **(B)** The Kar5 proteins from *S*. *cerevisiae*, *S*. *pombe*, *C*. *neoformans*, and *C*. *deneoformans* are drawn to scale. CnKar5 and CdKar5 protein domain structures are conserved with ScKar5 and SpKar5. All four proteins contain a cysteine-rich domain (C) indicated by yellow lines, a coiled-coil domain (Coil) indicated by green boxes, and C-terminal transmembrane domains (TM) indicated by cyan boxes. ScKar5, CnKar5, and CdKar5 contain an N-terminal signal peptide (SP) indicated by a blue box.

To study cell-cell fusion during the *Cryptococcus* sexual cycles, we performed BLASTP searches to identify plasma membrane fusion protein, Prm1, known to orchestrate cell-cell fusion during mating in other fungi. BLASTP searches using *S*. *cerevisiae*, *C*. *albicans*, *Aspergillus fumigatus*, *S*. *pombe*, and *N*. *crassa* Prm1 protein sequences [28–30] identified CNAG_05866 (Cn Prm1) and CNF01070 (CdPrm1) as candidate *PRM1* genes in *C*. *neoformans* and *C*. *deneoformans*, respectively (S1A and S1B Fig). The CnPrm1 and CdPrm1 proteins share 91% sequence identity and are the only candidate proteins that shared significant sequence similarity with Prm1 proteins from other fungal organisms. Reciprocal BLASTP searches confirmed the orthologous nature of these fungal *PRM1* genes. Both CnPrm1 and CdPrm1 are predicted to share a similar protein topology with ScPrm1 and SpPrm1, and contain four transmembrane domains based on Phobius prediction [44]. However, the *Cryptococcus* Prm1 proteins have a long C-terminal tail following the last transmembrane domain (Fig 1A).

Another crucial cellular process during sexual reproduction is karyogamy, the fusion of nuclei. One of the karyogamy proteins in *S*. *cerevisiae*, Kar5, facilitates nuclear membrane fusion during mating [40, 42]. We identified CNAG_04850 as the *KAR5* gene in *C*. *neoformans* using the Kar5 protein sequence of *Puccinia graminis* [43], which belongs to the same phylum (Basidiomycota) as *Cryptococcus*. The same BLASTP search failed to identify the *CdKAR5* gene, but using the *CnKAR5* genomic sequence we identified an unannotated region on chromosome 10 from bp 790071 to 792560 that encodes the *KAR5* ortholog in *C*. *deneoformans*. BLASTP searches and phylogenetic analyses of Kar5 proteins from several fungal organisms suggested that Kar5 protein sequences are divergent across different fungal species (S1C and S1D Fig). Multiple sequence alignment and topology predictions by Phobius prediction and COILS/PCOILS confirmed that CnKar5 and CdKar5 share a similar protein topology with ScKar5 and SpKar5, with an N-terminal signal peptide and a CRD domain, followed by coiled-coiled domains and a C-terminal transmembrane domain, except that SpKar5 does not have the N-terminal signal peptide (Fig 1B and S1E Fig) [44, 45].

### *PRM1* is required for cell-cell fusion during *C*. *neoformans* bisexual reproduction

**Fig 2:**
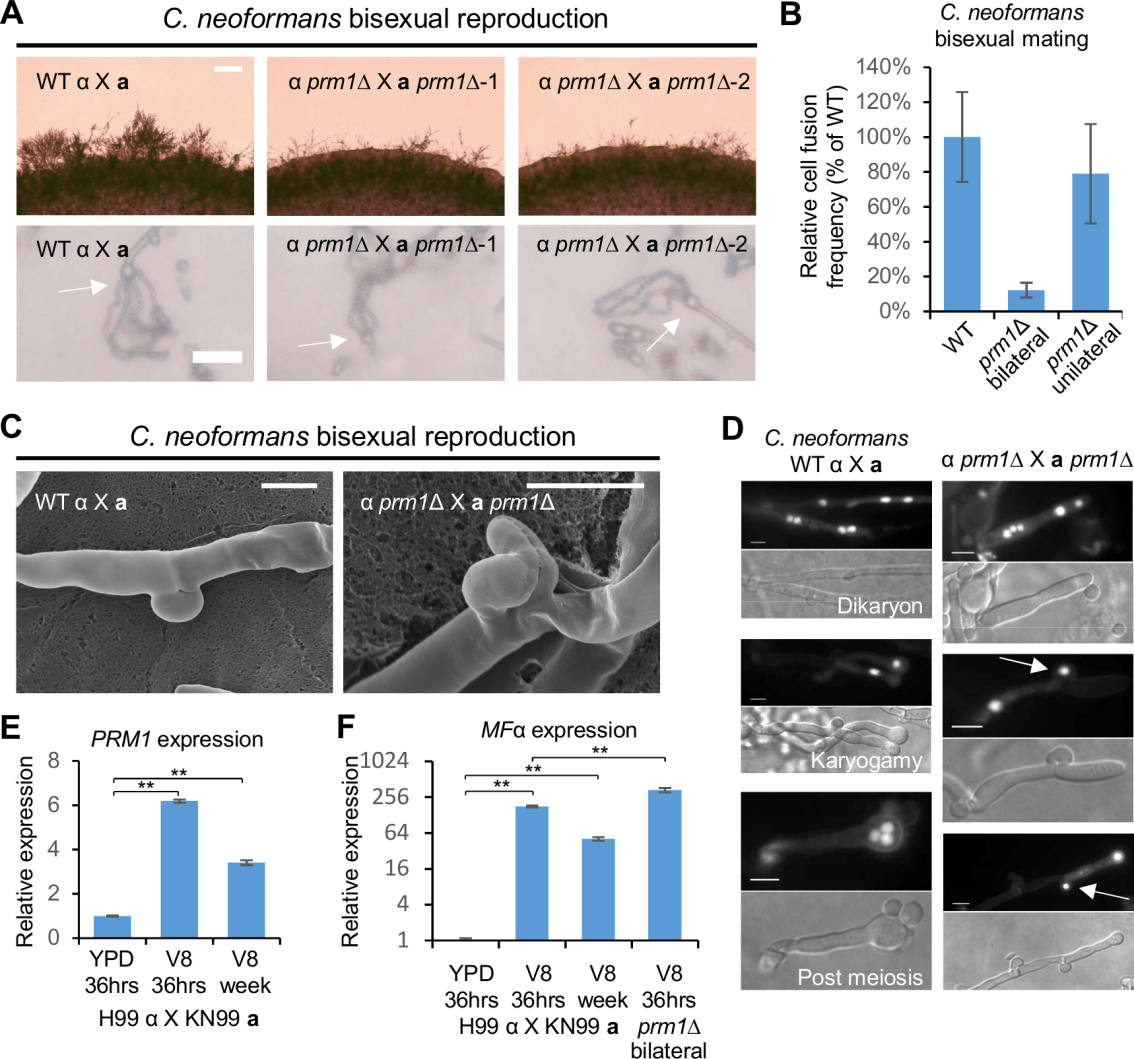
Deletion of *PRM1* blocks cell-cell fusion and clamp cell fusion during *Cryptococcus neoformans* bisexual reproduction. **(A)** Mating phenotypes for a wild type cross between H99α and KN99**a** and two independent *prm1* bilateral mutant crosses between CF30 and CF448, and between CF56 and CF562. All mating patches were spotted on MS medium and incubated in the dark at room temperature. The top row shows hyphal growth on the edge of mating patches five days after inoculation. The scale bar is 100 μm. The bottom row shows the basidium and spore chain morphology (indicated by arrows)10 days after inoculation. The scale bar equals 20 μm. **(B)** Unilateral and bilateral *prm1* mutant cell fusion frequency compared to wild type. **(C)** Scanning electron microscopy of clamp cell morphology of wild type cross (H99α X KN99**a**) and *prm1* bilateral mutant cross (CF56 X CF562). The scale bar is 5 μm. **(D)** DAPI staining of mature hyphae and nuclei inside hyphae and basidia of *C*. *neoformans* wild type (left panel) and *prm1* (right panel) bisexual crosses. Arrows in the *prm1* column indicate nuclei trapped in unfused clamp cells. The scale bar is 5 μm. Gene expression patterns for **(E)** *PRM1* and **(F)** *MF*α were examined by RT-PCR (* indicates *p* <0.05 and ** indicates *p* <0.005 for each pairwise comparison). A wild type cross (H99α X KN99**a**) was grown on YPD medium for 36 hours, and on V8 medium for 36 hours or one week. The *prm1* bilateral mutant cross (CF56 X CF562) was grown on V8 medium for 36 hours. The Y axis for panel F is in base-2 log scale. The error bars represent the standard deviation of the mean for the three biological replicates.

Deletion of *PRM1* caused a significant filamentation delay during *C*. *neoformans* bisexual reproduction (Fig 2A). However, abundant hyphal production and sporulation were still observed after 10 days (S2A Fig). To evaluate the overall impact of *PRM1* deletion on overall mating progress, we quantified the relative spore production of *prm1* mutants compared to the wild type at 7 days by Percoll gradient centrifugation. Deletion of *PRM1* caused a mild reduction in spore production (87.3 ± 9% of wild type, *p* = 0.207) (S3A Fig).

We conducted a wild type mating between CF757 (JEC20**a** *URA5-NAT*) and CF762 (JEC21α *ADE2-NEO*) as a control. A total of 47 spore derived colonies were randomly chosen and analyzed (S4A Fig). Among the 47 progeny, all eight genotypes of Mendelian inheritance were recovered at a distribution of frequency ranging from 2.1% to 23.4% (17% parental genotype *MAT***a** *URA5-NAT*, 2.1% for parental genotype *MAT*α *ADE2-NEO*, 12.8% for *MAT*α *URA5-NAT*, 23.4% for *MAT***a** *ADE2-NEO*, 6.4% for *MAT***a** *URA5-NAT ADE2-NEO*, 6.4% for *MAT*α *URA5-NAT ADE2-NEO*, 17% for *MAT***a**, and 14.7% for *MAT*α) (S4B and S4C Fig). This provides evidence that the cells isolated by Percoll gradient centrifugation are indeed spores.

To address the involvement of Prm1 in cell-cell fusion, we performed cell fusion assays using two genetically marked mating partners. *prm1* mutants showed a bilateral (*prm1*Δ X *prm1*Δ) cell fusion defect with a fusion frequency of 12% ± 4% relative to the wild type level (Fig 2B), but no defect in unilateral (*prm1*Δ X WT) cell fusion. The basal level of cell fusion activity may allow *prm1* mutants to produce abundant hyphae after a 10-day incubation on mating inducing medium (S2A Fig).

During *C*. *neoformans* bisexual reproduction, the dikaryotic hyphae generate clamp cells, which fuse with adjacent hyphal compartments to allow a nucleus to translocate between hyphal compartments and maintain the dikaryon status [6]. To test whether Prm1 plays a role in clamp cell-hyphal fusion, we examined hyphae by scanning electron microscopy (SEM). The clamp cell and a peg from the adjacent hyphal compartment both exhibited elongated tubular morphology in *prm1* mutants compared to clamp cell connections in the wild type (Fig 2C), suggesting that these clamp cells and peg protrusions failed to undergo cell fusion. Transmission election microscopy (TEM) showed that the plasma membranes failed to undergo fusion in the clamp cells (S5 Fig). DAPI staining of hyphal nuclei showed that a single nucleus was trapped in the *prm1* mutant clamp cells, resulting in an abnormal number of nuclei in a single hyphal compartment (Fig 2D).

**Fig 3:**
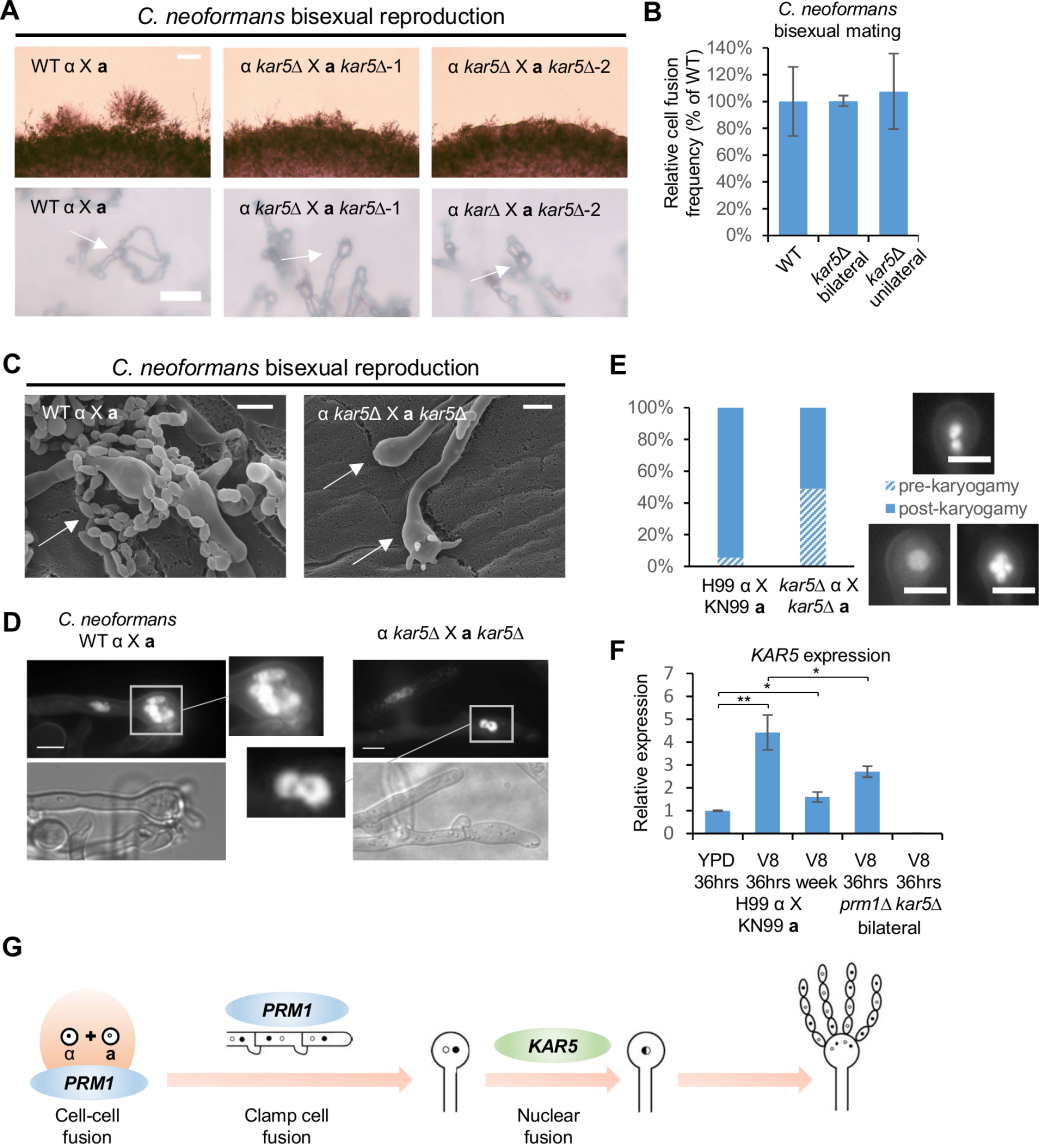
Deletion of *KAR5* causes a sporulation defect during *C*. *neoformans* bisexual reproduction. **(A)** Mating phenotypes for a wild type cross between H99α and KN99**a** and two independent *kar5* bilateral mutant crosses between CF57 and CF549, and CF208 and CF305. The scale bars are 100 μm and 20 μm for top row and bottom row, respectively. **(B)** Unilateral and bilateral *kar5* mutant cell fusion frequency compared to wild type. **(C)** Scanning electron microscopy of basidium morphology and sporulation patterns (indicated by arrows) for wild type cross (H99α X KN99**a**) and *kar5* bilateral mutant cross (CF57 X CF549). The scale bar is 5 μm. **(D)** DAPI staining of nuclei inside basidia from *C*. *neoformans* wild type (left panel) and *kar5* mutant (right panel) bisexual crosses. Basidia indicated by white boxes were magnified to show nuclei morphology. The scale bar is 5 μm. **(E)** Quantification of pre-karyogamy and post-karyogamy events for wild type and *kar5* mutant crosses based on DAPI staining of nuclei inside basidia. Representative pre-karyogamy (two nuclei) and post-karyogamy (one nucleus and post meiosis) events were shown on the right. The scale bar is 5 μm. **(F)** Gene expression patterns for *KAR5* were examined by RT-PCR (* indicates *p* <0.05 and ** indicates *p* <0.005 for each pairwise comparison). Wild type cross (H99α X KN99**a**) was grown on YPD medium for 36 hours, and on V8 medium for 36 hours or one week. *prm1* bilateral mutant cross (CF56 X CF562) and *kar5* bilateral mutant cross (CF57 X CF549) were grown on V8 medium for 36 hours. The error bars represent the standard deviation of the mean for the three biological replicates. **(G)** Proposed *C*. *neoformans* bisexual reproduction model. *PRM1* is required for cell-cell fusion and clamp cell fusion, and *KAR5* is required for karyogamy inside the basidium.

Clamp cell fusion is regulated by the pheromone signaling pathway, both *PRM1* and *MF*α expression were maintained at a significantly high level after mating for seven days on mating inducing V8 medium compared to non-mating inducing YPD medium (3.4-fold increase for *PRM1*, *p* <0.005; and 50.8-fold increase for *MF*α, *p* <0.005) (Fig 2E and 2F). *prm1* mutants exhibited a significant increase in *MF*α expression compared to wild type (1.9-fold increase, *p* <0.005), suggesting that the cell fusion defect dampens *MF*α repression that occurs in response to *SXI1*α-*SXI***a** repression following nuclear pairing (Fig 2F). These results indicate that Prm1 plays a role in both cell-cell fusion and clamp cell-hyphal fusion during *C*. *neoformans* bisexual reproduction (Fig 3G).

### *KAR5* is required for karyogamy during *C*. *neoformans* bisexual reproduction

Like *prm1* mutants, *kar5* mutants showed a significant delay in filamentation during *C*. *neoformans* bisexual reproduction (Fig 3A); the mutants produced abundant hyphae after 10 days (S2B Fig). In contrast to other *prm1* mutant phenotypes, *kar5* mutants were not defective in cell fusion but exhibited sporulation defects (Fig 3A and 3B). SEM studies showed that the abnormal basidia were either bald or had more than four budding sites compared to the four sites in the wild type (Fig 3C). However, the wild type phenotype (four spore chains) was observed in *kar5* mutants after longer mating incubation periods. Similar to *prm1* mutants, deletion of *KAR5* caused a mild reduction in spore production (77.2% ± 8.8% *p* <0.05) (S3B Fig), suggesting that deletion of *KAR5* did not completely block sporulation. We stained the nuclei within the abnormal basidia generated by *kar5* mutants with DAPI and found two nuclei in close contact within the *kar5* mutant bald basidia in contrast to either one nucleus or four meiotic nuclei present in wild type basidia (Fig 3D and 3E). Quantification of 129 wild type basidia and 131 *kar5* mutant basidia stained with DAPI showed that 5.7% wild type basidia versus 48.9% *kar5* mutant basidia contained two nuclei, suggesting that deletion of *KAR5* inhibited, but did not completely block karyogamy inside the basidia (Fig 3E). The nuclear morphology of the *C*. *neoformans kar5* mutant was similar to the *kar5* mutant karyogamy phenotype in *S*. *cerevisiae* [42], supporting the hypothesis that *KAR5* plays a conserved role in mediating karyogamy during *C*. *neoformans* bisexual reproduction. *KAR5* expression was upregulated upon mating induction and maintained at a significantly high level after mating for a week compared to non-mating inducing conditions (1.6-fold increase, *p* <0.05). Deletion of *PRM1* significantly reduced *KAR5* expression (1.6-fold decrease, *p* <0.05), suggesting control of gene expression following cell-cell fusion during *C*. *neoformans* bisexual reproduction (Fig 3F and 3G).

### *PRM1* plays a central role during *C*. *deneoformans* bisexual reproduction

**Fig 4:**
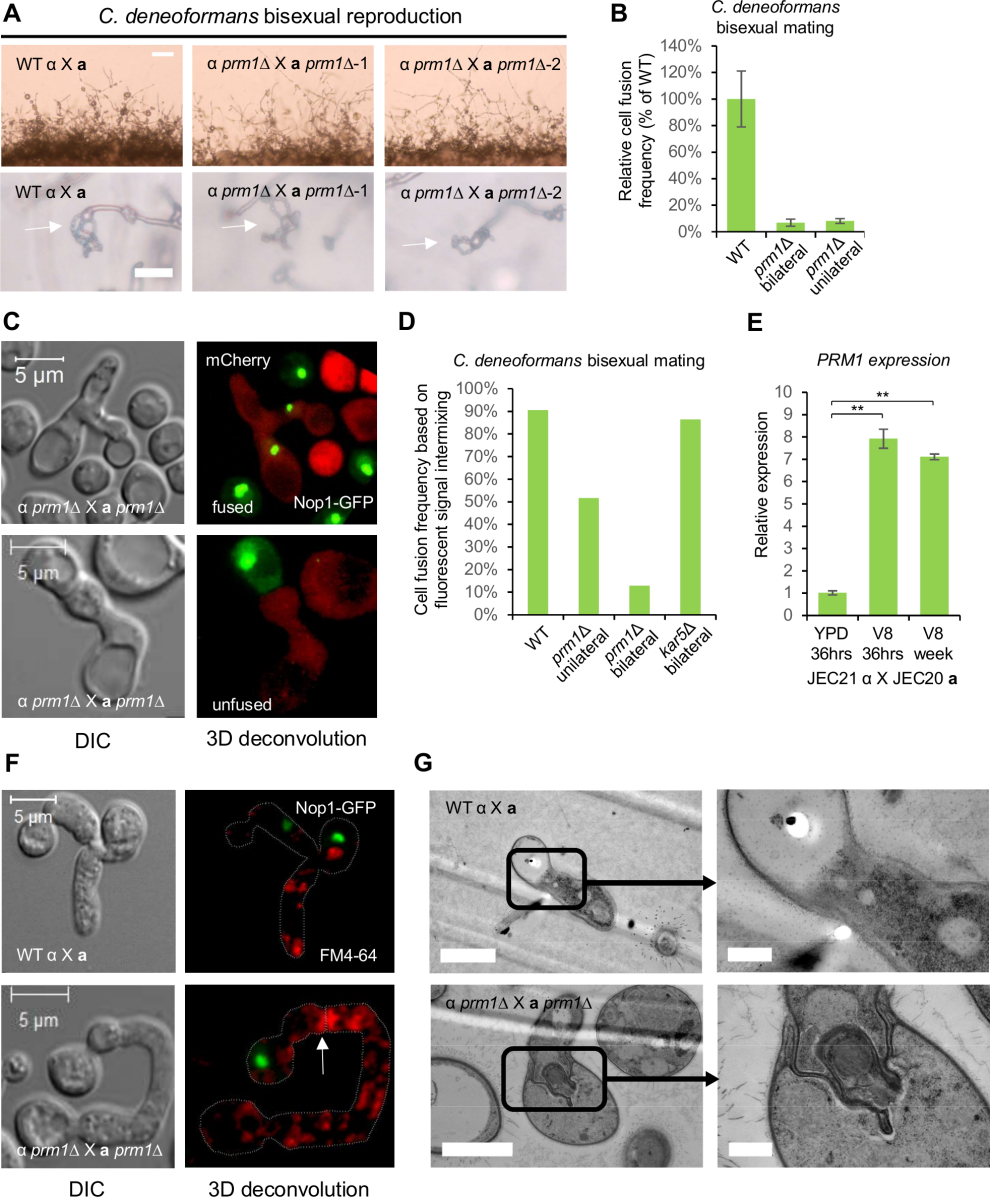
*prm1* mutants are defective in plasma membrane fusion during *C*. *deneoformans* bisexual reproduction. **(A)** Mating phenotypes for a wild type cross between JEC21α and JEC20**a** and two independent *prm1* bilateral mutant crosses between CF1 and CF313, and CF316 and CF517. The scale bars are 100 μm and 20 μm for top row and bottom row, respectively. **(B)** Unilateral and bilateral *prm1* mutant cell fusion frequency compared to wild type. **(C)** CF712 (JEC21α *prm1Δ*::*NAT mCherry-NEO*) was mated with CF768 (JEC20**a** *prm1Δ*::*NEO NOP1-GFP-NAT*) on V8 medium for 24 hours. Confocal microscopy showed that both fused and unfused cell fusion pairs were present during bilateral *prm1* mutant mating based on the presence or absence of fluorescent signal intermixing between fusion partners. The scale bar is 5 μm. **(D)** Wild type mating between CF830 (JEC21α *NOP1-GFP-NAT*) and JEC20**a**, unilateral mating between JEC21α and CF768 (JEC20**a** *prm1Δ*::*NEO NOP1-GFP-NAT*), bilateral mating between CF1 (JEC21α *prm1Δ*::*NEO*) and CF768 (JEC20**a** *prm1Δ*::*NEO NOP1-GFP-NAT*), and bilateral mating between CF487 (JEC21α *kar5*Δ::*NEO*) and CF723 (JEC20**a** *kar5*Δ::*NEO NOP1-GFP-NAT*) were conducted and the cell fusion frequency was determined based on GFP fluorescence signal intermixing between fusion partners. **(E)** Gene expression patterns for *PRM1* were examined by RT-PCR (* indicates *p* <0.05 and ** indicates *p* <0.005 for each pairwise comparison). Wild type cross (JEC21α X JEC20**a**) was grown on YPD medium for 36 hours, and on V8 medium for 36 hours or one week. The error bars represent the standard deviation of the mean for the three biological replicates. **(F)** Fusion pairs between CF830 (JEC21α *NOP1-GFP-NAT*) and JEC20**a**, and between CF1 (JEC21α *prm1Δ*::*NEO*) and CF768 (JEC20**a** *prm1Δ*::*NEO NOP1-GFP-NAT*) were stained with FM4-64 to show the plasma membrane structures at the conjugation sites. The scale bar is 20 μm. **(G)** Fused wild type cell fusion pair (top panel) between CF830 (JEC21α *NOP1-GFP-NAT*) and CF1076 (JEC20**a** *H3-mCherry-NAT*) and unfused cell fusion pair (bottom panel) between CF712 (JEC21α *prm1Δ*::*NAT mCherry-NEO*) and CF768 (JEC20**a** *prm1Δ*::*NEO NOP1-GFP-NAT*) were examined by transmission electron microscopy. Membrane structures at the conjugation sites were further examined at higher magnification. In the left panels, the scale bars are 2 μm, and in the right panels, the scale bars are 0.5 μm.

In contrast to *C*. *neoformans*, *C*. *deneoformans prm1* mutants showed a mild delay in hyphal production (Fig 4A), and exhibited a significant reduction in spore production compared to wild type *C*. *deneoformans* (27% ± 2.2%) bisexual reproduction (S3A Fig). *PRM1* deletion caused both bilateral and unilateral cell fusion defects with fusion frequencies of 6.9% ± 2.6% and 8.2% ± 1.8% of the wild type levels, respectively (Fig 4B). To understand the mechanistic requirement for Prm1 in cell-cell fusion during *C*. *deneoformans* bisexual reproduction, we monitored cell-cell fusion of *prm1* mutants with confocal microscopy. In the same *prm1* mutant cell fusion sample, both fused and unfused cells were detected by the presence and absence of inter-cellular mixing of fluorescent signals between the Nop1-GFP and mCherry labeled fusion pairs (Fig 4C, S1 and S2 Movies). Based on quantification of fluorescent signal intermixing, the wild type cell fusion frequency was 90.6%, while the *prm1* mutant unilateral cell fusion and bilateral cell fusion frequencies were 51.7% and 12.8%, respectively (Fig 4D and S6 Fig). The unilateral cell fusion defect suggests that the Prm1 plays a more important role during bisexual reproduction in *C*. *deneoformans* compared to *C*. *neoformans*; in agreement with this observation, *PRM1* expression level was maintained at a similar high level after mating for seven days compared with 36 hours (Fig 4E).

To visualize the structures of the plasma membrane at the conjugation sites between the fusion pairs, we stained both wild type and *prm1* mutant fusion pairs with the lipophilic dye FM4-64. The *prm1* mutant fusion pairs exhibited robust staining of the plasma membrane boundaries at the conjugation site (Fig 4F). Compared to the fused wild type cells, the plasma membrane of the *prm1* mutant fusion pairs failed to undergo membrane fusion at the conjugation sites, and a layer of cell wall material was present between the plasma membranes (Fig 4G and S7A Fig). In 2 out of 20 observed fusion pairs by TEM, the plasma membranes formed extensive invaginations into the opposite cytosolic compartments without membrane fusion (Fig 4G). Similar to *C*. *neoformans* bisexual reproduction, *prm1* mutants also exhibited a clamp cell-hyphal fusion defect during *C*. *deneoformans* bisexual reproduction (S7B Fig). However, both wild type and *prm1* mutant crosses produced hyphae with unfused clamp cells, which are characteristics of monokaryotic hyphae (S7B and S8A Figs). Overall, these results suggest that *PRM1* plays a more significant role in *C*. *deneoformans* bisexual reproduction in comparison to *C*. *neoformans*.

### Karyogamy occurs early during *C*. *deneoformans* bisexual reproduction

**Fig 5:**
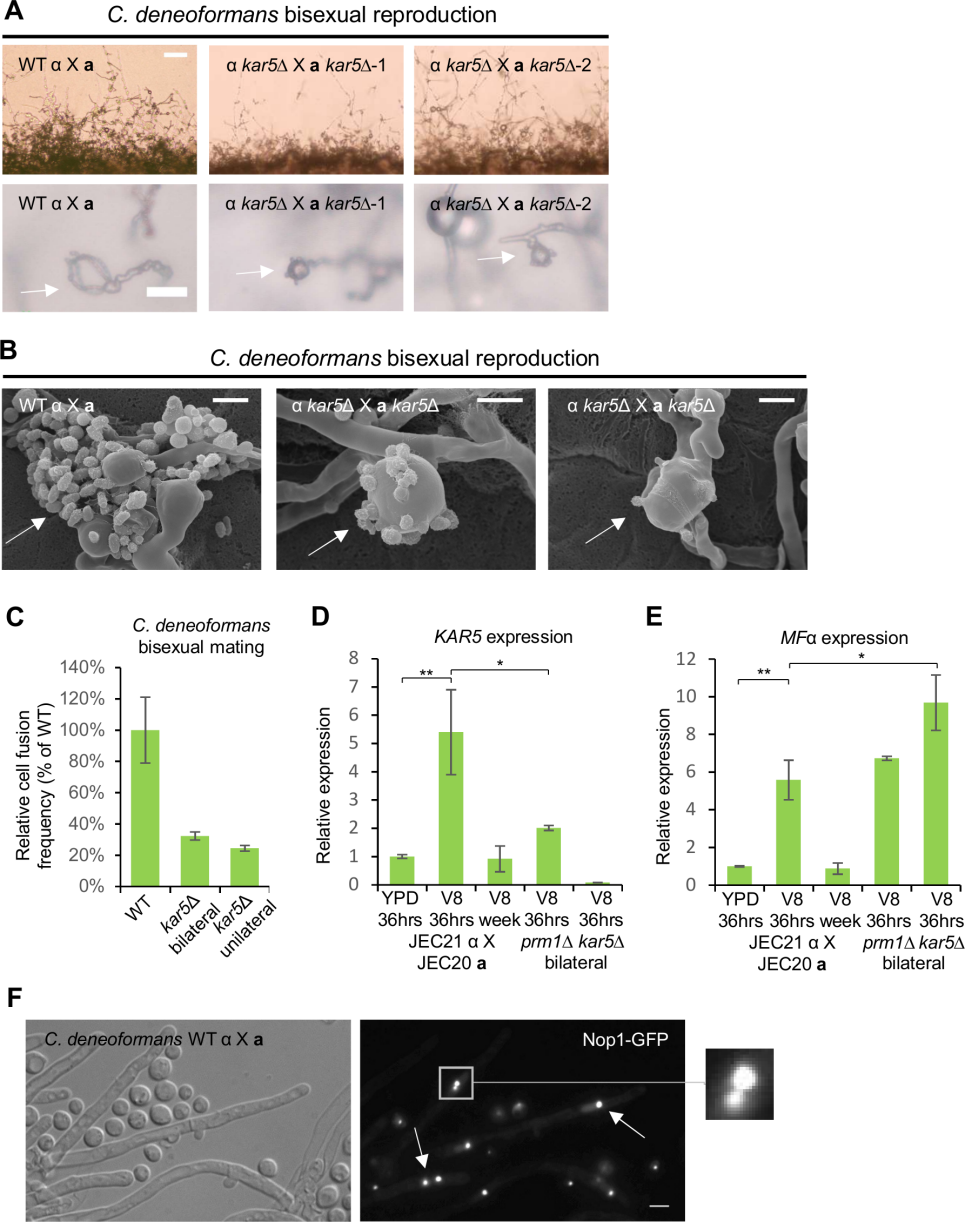
*kar5* mutants are defective in cell fusion and basidium sporulation during *C*. *deneoformans* bisexual reproduction. **(A)** Mating phenotypes for a wild type cross between JEC21α and JEC20**a** and two independent *kar5* bilateral mutant crosses between CF226 and CF364, and CF487 and CF464. The scale bars are 100 μm and 20 μm for top row and bottom row, respectively. **(B)** Scanning electron microscopy of basidia morphology and sporulation patterns (indicated by arrows) for wild type cross (JEC21α X JEC20**a**) and *kar5* bilateral mutant cross (CF226 X CF364). The scale bar is 5 μm. **(C)** Unilateral and bilateral *kar5* mutant cell fusion frequency compared to wild type. Gene expression patterns for **(D)** *KAR5* and **(E)** *MF*α were examined by RT-PCR (* indicates *p* <0.05 and ** indicates *p* <0.005 for each pairwise comparison). Wild type cross (JEC21α X JEC20**a**) was grown on YPD medium for 36 hours, and on V8 medium for 36 hours or one week. *prm1* bilateral mutant cross (CF1 X CF313) and *kar5* bilateral mutant cross (CF226 X CF364) were grown on V8 medium for 36 hours. The error bars represent the standard deviation of the mean for the three biological replicates. **(F)** The GFP labeled nucleolar marker Nop1 was used to study *C*. *neoformans* bisexual cross (CF830 α *NOP1-GFP-NAT* X JEC20**a**) hyphal nuclear morphology. Arrows indicate instances of both monokaryotic and dikaryotic hyphae during early stages of bisexual reproduction, and the area within the box was magnified to highlight two nuclei in close contact.

Like *prm1* mutants, *kar5* mutants showed a mild delay in hyphal production, and produced significantly fewer spores compared to wild type (9.5% ± 2.7%) (Fig 5A and S3B Fig). SEM studies demonstrated that *C*. *deneoformans kar5* mutants produced basidia with abnormal sporulation patterns during bisexual reproduction, similar to *C*. *neoformans kar5* mutants (Fig 5B). Deletion of *KAR5* caused both bilateral and unilateral cell fusion defects with fusion frequencies of 32.3% ± 6% and 24.5% ± 2.7% compared to the wild type level, respectively (Fig 5C). To test whether *KAR5* is directly involved in cell-cell fusion, we quantified cell-cell fusion events for *kar5* mutants based on fluorescent signal intermixing, and found that *kar5* mutant cell-cell fusion frequency was 86.4%, similar to wild type (Fig 4D and S6D Fig), suggesting that Kar5 plays a role in post-fusion survival mechanisms for cell fusion products, and that the function of CdKar5 in bisexual reproduction is diverged from CnKar5 (Fig 3B, and 5C). Furthermore, *CdKAR5* and *CdMF*α expression were upregulated upon mating induction, but returned to basal level after mating for seven days, which is distinct from *CnKAR5* and *CnMF*α expression patterns (Figs 2F, 3F, 5D and 5E). However, *CdKAR5* expression was significantly reduced for *Cdprm1* mutants compared to wild type (2.7-fold decrease, *p* <0.05) (Fig 5D), similar to *C*. *neoformans* (Fig 3F).

To elucidate the phenotypic differences of *prm1* and *kar5* mutants during bisexual reproduction between *C*. *neoformans* and *C*. *deneoformans*, we stained wild type and mutant hyphal nuclei with DAPI. In contrast to the dikaryotic hyphae produced by *C*. *neoformans* (Fig 2D), *C*. *deneoformans* bisexual reproduction produced monokaryotic hyphae (S8A Fig), similar to those produced during *C*. *deneoformans* unisexual reproduction (S8B Fig). To dissect the involvement of Prm1 and Kar5 in monokaryotic hyphae formation during *C*. *deneoformans* bisexual mating, we tracked nuclear dynamics using the nucleolar marker Nop1-GFP [38]. During early bisexual mating at 48 hours, wild type produced both monokaryotic and dikaryotic hyphae (Fig 5F and S9A Fig), whereas *prm1* mutants mainly produced monokaryotic hyphae (S9A Fig). In both wild type and *kar5* mutant hyphae, pairs of congressed nuclei were observed, resembled the *C*. *neoformans kar5* mutant karyogamy phenotype inside basidia during bisexual reproduction (Figs 3D and 5F and S9A Fig). After 10 days, monokaryotic and dikaryotic hyphae were present in the wild type cross, while *prm1* mutants mainly produced monokaryotic hyphae and *kar5* mutants mainly produced dikaryotic hyphae (S9B Fig). After six weeks, wild type and *prm1* mutants mainly produced monokaryotic hyphae, whereas, *kar5* mutants produced both monokaryotic and dikaryotic hyphae (S9C Fig). Live cell imaging of hyphal nuclear morphology suggests the following: 1) karyogamy may take place early in bisexual reproduction in *C*. *deneoformans*; 2) deletion of *PRM1* leads to monokaryotic hyphae formation; and 3) deletion of *KAR5* blocks early karyogamy in fused cells and could explain the observed post-fusion survival defect for the fused cells, which in turn promoted dikaryon hyphae formation.

**Fig 6:**
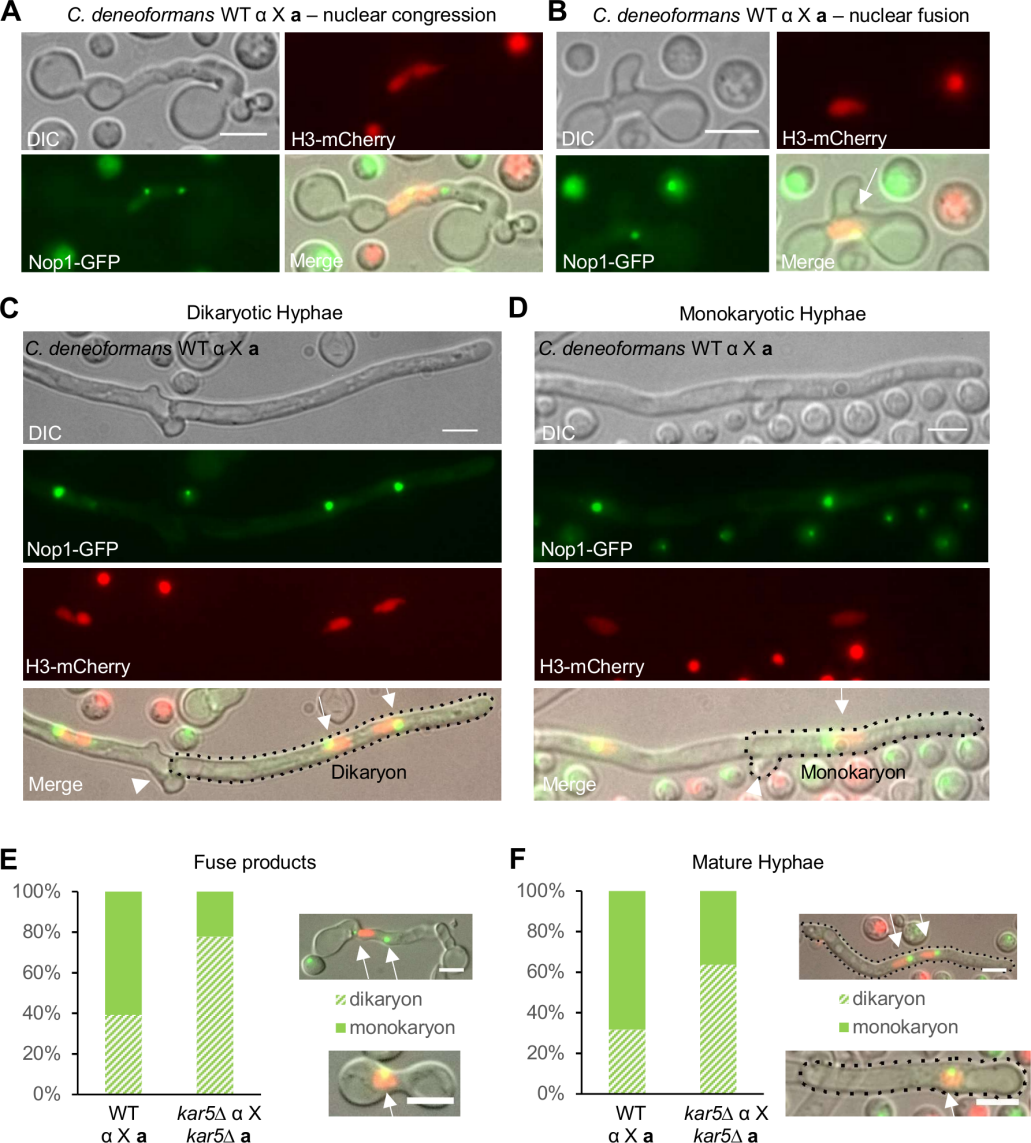
Karyogamy occurs at different stages during *C*. *deneoformans* bisexual reproduction. GFP labeled nucleolar marker Nop1 and mCherry labeled nuclear marker Histone H3 protein were used to study the formation of monokaryotic hyphae during *C*. *denoeformans* bisexual mating (CF830 α *NOP1-GFP-NAT* X CF1076 **a** *H3-mCherry-NAT*). **(A)** Nuclear congression occurs in **a**-α fused cells. **(B)** Nuclear fusion occurs in **a**-α fused cells. Arrow points to the fused nucleus, as indicated by the mixing of the fluorescent signals. **(C-D)** *C*. *deneoformans* bisexual reproduction produces both **(C)** dikaryotic hyphae with fused clamp cells and **(D)** monokaryotic hyphae with unfused clamp cells. Single hyphal compartments are marked with dotted circles. Arrows point to nuclei labeled with both GFP and mCherry. Arrowheads point to a fused clamp cell in panel C and an unfused clamp cell in panel D. The scale bar is 5 μm. **(E-F)** Quantification of monokaryon and dikaryon fusion products **(E)** or mature hyphae **(F)** for wild type (CF830 α *NOP1-GFP-NAT* X CF1076 **a** *H3-mCherry-NAT*) and *kar5* mutant (CF1185 α *kar5*Δ::*NEO H3-mCherry-NAT* X CF723 **a** *kar5*Δ::*NEO NOP1-GFP-NAT*) crosses. Representative dikaryon and monokaryon fusion products are shown on the right. Single hyphal compartments are marked with dotted circles, and each arrow points to one nucleus. The scale bar is 5 μm.

To confirm that karyogamy occurs early during *C*. *deneoformans* bisexual reproduction, we followed the hyphal nuclear dynamics between mating partners labeled with fluorescent markers (nucleolar marker Nop1-GFP and nuclear marker H3-mCherry). We observed nuclear congression in fused **a**-α cells (Fig 6A); and a single nucleus labeled with both fluorescent protein markers was observed, confirming that karyogamy can occur immediately after cell fusion (Fig 6B). We also observed both dikaryotic hyphae with fused clamp cells and monokaryotic hyphae with unfused clamp cells during the early mating process (Fig 6C and 6D). These hyphae expressed both parental fluorescent markers, indicating that karyogamy can occur at different stages during *C*. *deneoformans* bisexual reproduction. To test whether Kar5 functions in karyogamy immediately after cell fusion and deletion of *KAR5* leads to dikaryon formation, we quantified monokaryon and dikaryon fusion products and mature hyphae labeled with both nuclear fluorescent markers in wild type and *kar5* mutant crosses (Fig 6E and 6F). Among 125 wild type and 126 *kar5* mutant cell fusion products, 60.8% wild type versus 22.2% *kar5* mutant fused cells were monokaryotic (Fig 6E). Among 133 wild type and 132 *kar5* mutant mature hyphae, 68.4% wild type versus 36.4% *kar5* mutant mature hyphae were monokaryotic (Fig 6F). These results confirmed that deletion of *KAR5* inhibited, but did not completely block karyogamy in early cell fusion products, and promoted dikaryon formation.

### *PRM1* and *KAR5* are largely dispensable for *C*. *deneoformans* solo unisexual reproduction

**Fig 7:**
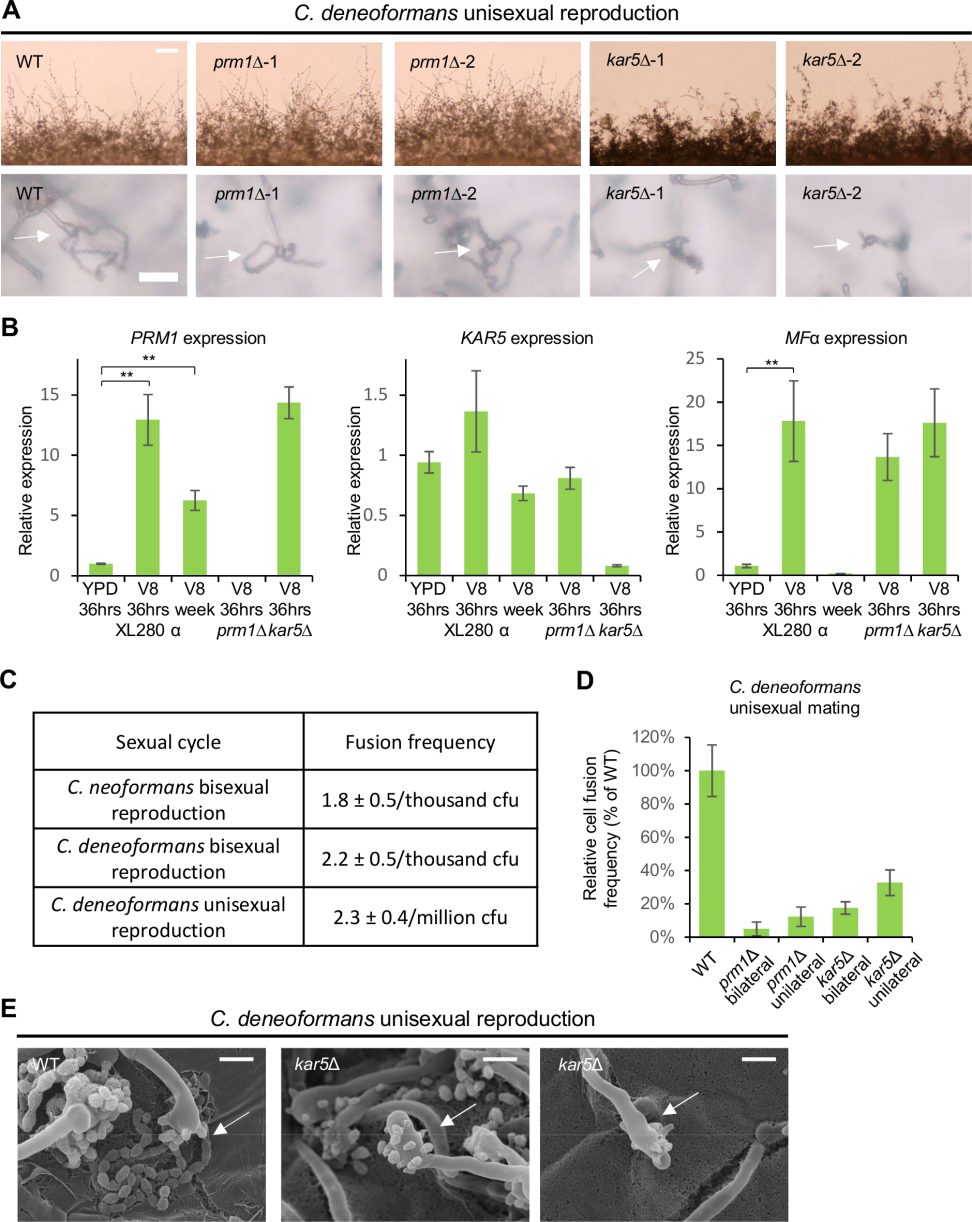
*PRM1* and *KAR5* are largely dispensable for unisexual reproduction. **(A)** Mating phenotypes for wild type XL280α, two independent *prm1* mutants (CF317 and CF659), and two independent *kar5* mutants (CF150 and CF260) during *C*. *deneoformans* unisexual reproduction. The scale bars are 100 μm and 20 μm for top row and bottom row, respectively. **(B)** Gene expression patterns for *PRM1*, *KAR5*, and *MF*α were examined by RT-PCR (* indicates *p* <0.05 and ** indicates *p* <0.005 for each pairwise comparison). Wild type (XL280α) was grown on YPD medium for 36 hours, and on V8 medium for 36 hours or one week. *prm1* mutant (CF317) and *kar5* mutant (CF150) were grown on V8 medium for 36 hours. The error bars represent the standard deviation of the mean for the three biological replicates. **(C)** Comparison of wild type cell-cell fusion frequency among three different sexual cycles between *C*. *neoformans* and *C*. *deneoformans*. **(D)** Unilateral and bilateral *prm1* mutant and *kar5* mutant cell fusion frequency compared to wild type. **(E)** Scanning electron microscopy of basidium morphology and sporulation patterns (indicated by arrows) of the wild type (XL280α) and the *kar5* mutant (CF150). The scale bar is 5 μm.

In contrast to bisexual reproduction, deletion of *PRM1* and *KAR5* in *C*. *deneoformans* did not impact filamentation during solo unisexual reproduction (Fig 7A), and caused less reduction in spore production relative to the wild type level (57.3% ± 7.2% and 52.8% ± 4.6%, respectively) (Fig 7A and S3 Fig). *PRM1* and *MF*α expression were upregulated upon mating induction, but, *KAR5* expression was maintained at a low level and was not affected by pheromone induction (Fig 7B), suggesting *KAR5* may play a less important role in solo unisexual reproduction. Similar to what is seen during bisexual reproduction in *C*. *deneoformans*, *PRM1* expression was maintained at a significantly high level after mating for seven days compared to non-mating inducing conditions (6.2-fold increase, *p* <0.005), whereas pheromone signaling subsided to basal level, indicating that *PRM1* expression is not tightly coordinated with the pheromone signaling pathway in *C*. *deneoformans* (Fig 7B). Although *PRM1* expression was significantly upregulated, cell fusion occurred at a 1000-fold lower frequency during unisexual reproduction in *C*. *deneoformans* compared to both *C*. *neoformans* and *C*. *deneoformans* bisexual reproduction (Fig 7C). Among cells that underwent cell-cell fusion during unisexual reproduction, deletion of *PRM1* and *KAR5* caused both bilateral and unilateral cell fusion defects (Fig 7D), and deletion of *KAR5* produced basidia with abnormal sporulation patterns (Fig 7E), which were also observed during bisexual reproduction. These results suggest that during unisexual reproduction, a minority of cells undergo α-α cell fusion followed by karyogamy, similar to *C*. *deneoformans* bisexual reproduction.

Although cell fusion is largely dispensable for solo unisexual reproduction, karyogamy may function independently of cell fusion between mother and daughter cells or inside basidium. Deletion of *KAR7* has been indicated to block nuclear congression inside the basidium during unisexual reproduction [38]. To test whether *KAR5* has similar functions, we stained wild type, *kar5* mutant, and *kar7* mutant basidia with DAPI. Interestingly, all strains produced basidia with one, two, or more than two nuclei, which may represent three different stages of meiosis inside basidia (one nucleus as pre-meiosis, two nuclei as post meiosis I, and more than two nuclei as post meiosis II) (S10A Fig). Among 114 wild type, 116 *kar5* mutant, and 115 *kar7* mutant basidia, only 1.8% wild type, 4.3% *kar5* mutant, and 1.7% *kar7* mutant basidia contained two nuclei (S10B Fig), which is different from the *cnkar5* mutant with 48.9% basidia containing two pre-karyogamy nuclei during bisexual reproduction (Fig 3E), suggesting that nuclear fusion occurs differently during unisexual reproduction of strain XL280α. If *KAR5* and *KAR7* were required for karyogamy in the basidia, we would have expected to see a higher population of basidia with 2 nuclei trapped at a pre-karyogamy stage compared to wild type. However, wild type and *kar5* mutants exhibited similar basidia nuclear morphology with few two nuclei basidia (S10B Fig), indicating that *KAR5* is not required for a later stage of unisexual reproduction, and nuclear fusion is not occurring inside the basidium. The *kar7* mutant produced 24.3% basidia versus 60.5% basidia in wild type with more than two nuclei (S10B Fig), suggesting that *KAR7* plays a role in meiosis during unisexual reproduction, supporting the previous observation that a diploid *kar7*/*kar7* mutant has a defect in sporulation [38]. To validate these results, we examined basidia nuclear morphology based on nuclear fluorescent signals of wild type (CF836), *kar5* mutant (CF718), and *kar7* mutant (CF1442) cells labeled with Nop1-GFP, and observed similar results (S11 Fig), supporting the hypothesis that karyogamy occurs at a low frequency and karyogamy defects do not impact basidia nuclear morphology during solo unisexual reproduction.

Given that cell fusion is dispensable in solo unisexual reproduction, and *kar5* is not required for meiotic basidia formation, we aimed to confirm that meiosis was involved during spore production. We generated *prm1 spo11* and *kar5 spo11* double mutants and observed two short spore chains compared to the four long spore chains produced by *prm1* and *kar5* single mutants (S12 Fig). The lack of normal spore chains confirms that spore production in unisexual reproduction is indeed dependent on the key meiotic gene *SPO11* as shown previously [8].

### Diploidization is achieved early in hyphae during unisexual reproduction in *C*. *deneoformans*

It is unclear how diploidization occurs during solo unisexual reproduction. By following mating partners of the same mating type labeled with different fluorescent markers (nucleolar marker Nop1-GFP and nuclear marker H3-mCherry), we showed that hyphae frequently originated from single cells rather than as of α-α cell fusion products (S3 Movie), which further confirmed that cell fusion is dispensable for the solo yeast-hyphal morphological transition.

**Fig 8:**
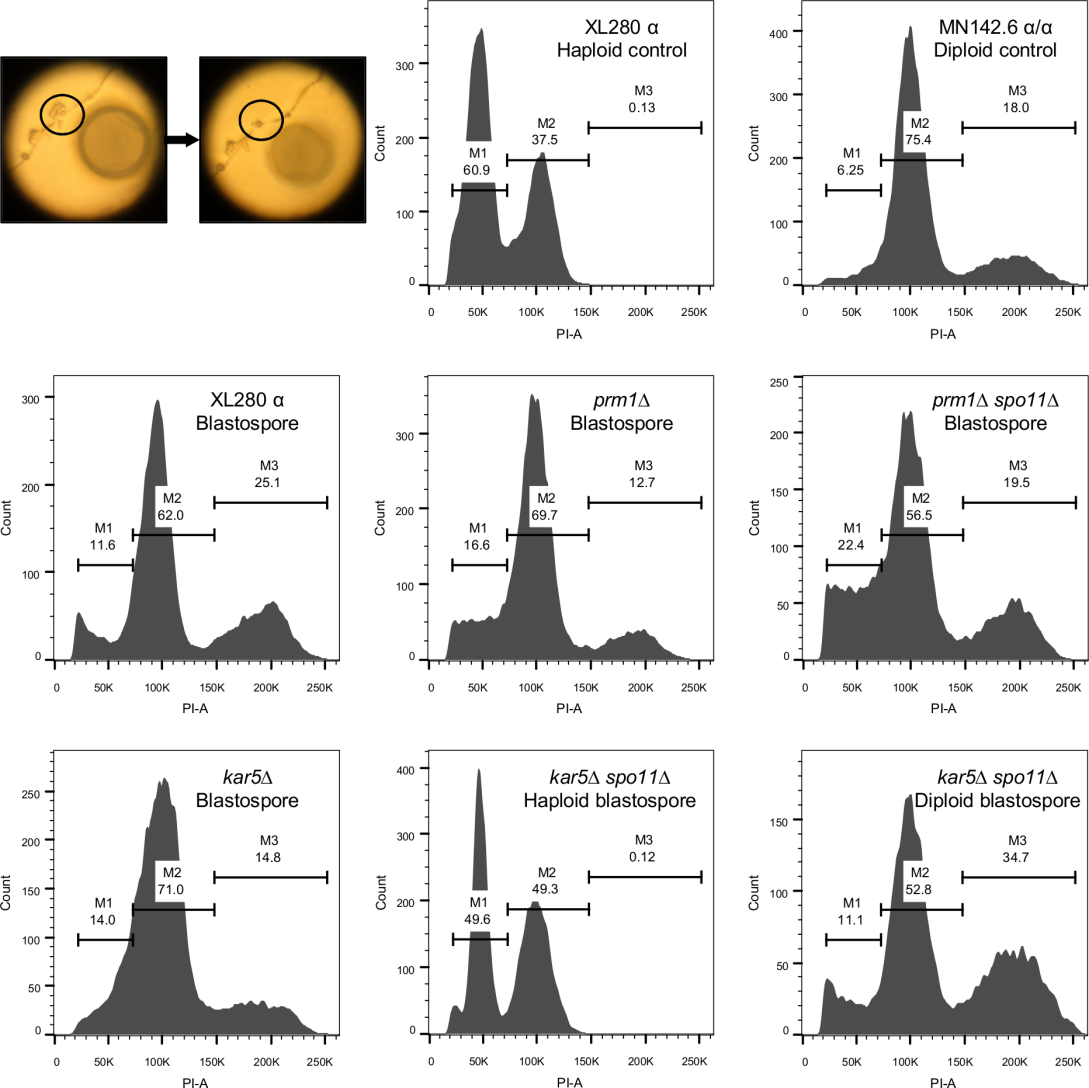
Ploidy determination by FACS for blastospores produced during unisexual reproduction. The upper left panel is the diagram for dissection of blastospores. The circle at left is before and the circle at right is after the blastospores were removed for dissection. The upper middle and right panels are FACS results for haploid control XL280α and diploid control MN142.6 α/α. The middle and lower panels are representative FACS results for blastospores produced by the indicated strains. Wild type XL280α, *prm1*Δ, *prm1*Δ *spo11*Δ, and *kar5*Δ produced diploid blastospores, whereas, *kar5*Δ *spo11*Δ produced both haploid and diploid blastospores.

To understand when and where diploidization takes place during solo unisexual reproduction, we dissected nascent blastospores from the growing hyphae (Fig 8) and analyzed their ploidy by FACS (Table 1 and S3 Table). In the wild type, 66 out of 71 blastospores dissected from eight budding sites germinated with a survival rate of 93%. FACS analysis of 16 blastospore derived colonies, including two from each budding site, showed that all were diploid (Fig 8). *prm1* and *kar5* mutants exhibited blastospore germination defects with survival rates of 19.2% and 62.1% respectively. *prm1 spo11* and *kar5 spo11* double mutants exhibited blastospore survival rates of 63.3% and 92.5% respectively. FACS analysis revealed all blastospores of the *prm1* mutant, 12 blastospores from 6 budding sites (four blastospores from two budding sites failed to germinate) of the *prm1 spo11* double mutant, and 14 blastospores from seven budding sites (two blastospores from one budding site failed to germinate) of the *kar5* mutant were diploid. In analysis of 19 blastospores from 10 budding sites in the *kar5 spo11* double mutant, 6 blastospores were haploid, and 13 were diploid (Fig 8). The six haploid blastospores were dissected from three budding sites, suggesting that blastospores originating from the same budding site may have the same ploidy composition. To infer whether the observed single nucleus in the *kar7* mutant basidia might be a product of karyogamy (S10 and S11 Figs), we dissected 248 blastospores from 46 budding sites for the *kar7* mutant, and only 16 blastospores from 10 budding sites germinated with a survival rate of 6.45%, suggesting Kar7 is required for wild type blastospore survival (Table 1 and S3 Table). Among 15 blastospores analyzed, 9 were diploid, 3 were haploid, and 3 were aneuploid (Table 1 and S13 Fig), suggesting that the nuclei inside *kar7* mutant basidia are likely largely diploid and that diploidization occurs earlier and outside of the basidium. The limited sample size of dissected blastospores presented here may explain why a few haploid blastospores were only recovered from the *kar5 spo11* double mutant and *kar7* mutant but not from wild type or the other mutant strains. That 74 out of a total of 86 (86%) tested blastospores were diploid suggests that diploidization occurs early in the hyphae during unisexual reproduction, and this process may be dependent on an endoreplication pathway or early karyogamy events between mother and daughter cells or inside the growing hyphae. Prm1 and Kar5 were dispensable for diploidization, but may contribute to blastospore survival, implying that Prm1 and Kar5 could have additional cellular functions.

**Table 1:** Ploidy determination by FACS for blastospores produced during unisexual development.

Strain	Budding sites dissected (n)	Blastospores dissected (n)	Blastospores germinated (n)	Germination rate	Blastospores tested for ploidy* (n)	Diploid (n)	Haploid (n)	Aneuploid (n)
**XL280α**	8	71	66	93.0%	16	16	0	0
***prm1*Δ**	8	52	10	19.2%	10	10	0	0
***prm1*Δ *spo11*Δ**	8	60	38	63.3%	12	12	0	0
***kar5*Δ**	8	58	36	62.1%	14	14	0	0
***kar5*Δ *spo11*Δ**	10	40	37	92.5%	19	13	6	0
***kar7*Δ**	46**	248	16	6.45%	15	9	3	3

* For each budding site, no more than two blastospores were chosen for FACS determination of ploidy.

** Out of the 46 budding sites dissected for *kar7*Δ mutant, only 10 budding sites yielded germinated blastospores.

## Discussion

Without an obligate requirement for a mating partner, unisexual reproduction mitigates the two-fold cost of bisexual reproduction in finding an opposite mate. However, lacking genome diversity, clonal unisexual reproduction could be considered an evolutionary dead-end. In *Cryptococcus*, this assumption is challenged, as unisexual reproduction can generate genotypic and phenotypic diversity *de novo* by forming aneuploid progeny through meiosis [17]. Given that more than 99% of the natural isolates are α mating type, the presence of a unisexual cycle allows a clonal population to adapt to changing environments, which provides ecological significance to the *Cryptococcus* pathogenic species complex [46]. In this study, we demonstrated that a small population of cells undergo cell-cell fusion and nuclear fusion during unisexual reproduction, which enables recombination between cells of the same mating type. In response to selection pressures in the environment, the cell fusion dependent unisexual reproduction could facilitate selection of beneficial alleles in a large same sex population and reverse Muller’s ratchet [22]. Same sex cell-cell fusion can be further stimulated by the presence of small population of the opposite mating type [7]. Besides the similar ecological benefits conferred by unisexual and bisexual reproduction, many studies have shown that both modes of sexual cycles share a common signaling network that regulates the yeast-to-hyphal morphological transition and meiotic recombination [25, 26, 47]. Despite the similarities, there are key mechanistic differences between the two. In this study, we focused on two key cellular processes involved in sexual reproduction, cell-cell fusion and nuclear fusion, and studied their involvement in unisexual and bisexual reproduction in two sister *Cryptococcus* species harboring different sexual cycles.

*Cryptococcus* orthologs of the *S*. *cerevisiae* cell fusion gene *PRM1* perform conserved roles during *Cryptococcus* sexual reproduction. Prm1 facilitates cell fusion between **a** and α mating partner cells, cell fusion between α-α cells, and clamp cell-hyphal fusion during dikaryotic hyphal growth. During *C*. *neoformans* bisexual reproduction, deletion of *PRM1* caused a bilateral (*prm1*Δ X *prm1*Δ) cell fusion defect, which is similar to what has been observed in *S*. *cerevisiae* and *N*. *crassa* [29, 31]. However, during *C*. *deneoformans* bisexual reproduction, deletion of *PRM1* caused both unilateral (*prm1*Δ X WT) and bilateral (*prm1*Δ X *prm1*Δ) cell fusion defects, suggesting that Prm1 plays a more significant role in *C*. *deneoformans*.

Cell-cell fusion and clamp cell-hyphal fusion in *Cryptococcus* is analogous to cell fusion between conidial anastomosis tubes and hyphal fusion in filamentous fungi [48, 49]. Like in *S*. *cerevisiae*, *N*. *crassa*, and *S*. *pombe*, deletion of *PRM1* resulted in plasma membrane curvature at the membrane merger site (Fig 4D), but these membranes were separated by a layer of cell wall (Fig 4G and S6 Fig), similar to the *prm1* mutant phenotype in *S*. *pombe*. Although deletion of *PRM1* caused a cell fusion defect, it did not completely block cell fusion in *Cryptococcus*, suggesting that Prm1 is not the sole membrane fusion protein. Additional candidate cell fusion genes have been identified in *S*. *cerevisiae* and *N*. *crassa*, including *FIG1*, *LFD1*, and *LFD2*; but BLASTP searches failed to identify homologs of these genes in *Cryptococcus* [31, 32]. Prm1 may be the evolutionary conserved core component for cell fusion in the fungal kingdom, and species-specific plasma membrane fusion machinery may have evolved independently.

Similarly, the *Cryptococcus* karyogamy machinery has been previously shown to function differently than that of *S*. *cerevisiae* [38]. Deletion of *KAR5* did not completely block either unisexual or bisexual reproduction, suggesting that additional karyogamy genes may have redundant functions with *KAR5*. The nuclear morphology inside *cnkar5* mutant basidia and *cdkar5* mutant early fusion products is similar to the *kar5* mutant karyogamy defect phenotype in *S*. *cerevisiae*, indicating *KAR5* plays a conserved role in nuclear fusion between *Saccharomyces* and *Cryptococcus* [41]. During *C*. *neoformans* bisexual reproduction, deletion of *KAR5* blocked nuclear fusion inside basidia, whereas, during *C*. *deneoformans* bisexual reproduction, deletion of *KAR5* blocked nuclear fusion at an early developmental step and caused growth arrest for the cell fusion products leading to an apparent cell fusion defect. Early karyogamy in *C*. *deneoformans* wild type relieved the requirement for pheromone signaling for directing clamp cell-hyphal fusion during dikaryotic hyphal growth, and the pheromone expression level was rapidly reduced to a basal level in the wild type. However, deletion of *KAR5* promoted dikaryotic hyphal growth, and as a consequence the pheromone signaling pathway in *kar5* mutants was significantly upregulated compared to the wild type. The pheromone expression patterns validated *KAR5*’s function in karyogamy.

**Fig 9:**
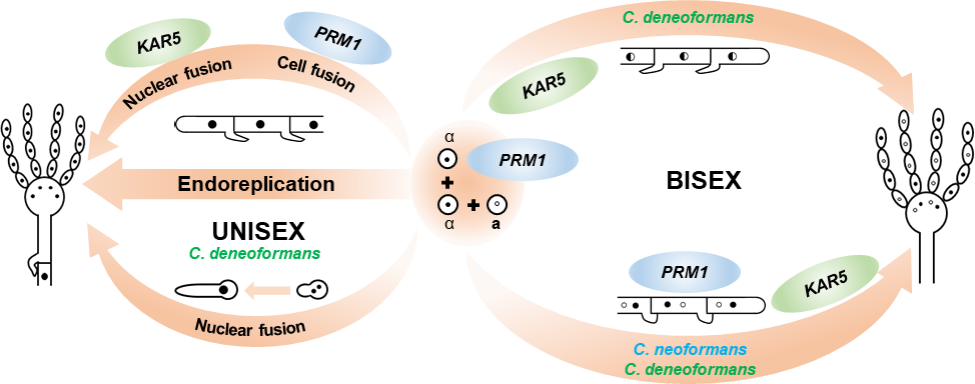
Sexual cycles in *Cryptococcus*. During *C*. *neoformans* bisexual reproduction, **a**-α cell-cell fusion generates dikaryotic hyphae and karyogamy occurs inside the basidia. During *C*. *deneoformans* bisexual reproduction, karyogamy takes place at different stages and generates both dikaryotic and monokaryotic diploid hyphae. During *C*. *deneoformans* unisexual reproduction, diploidization in the hyphae is achieved early during differentiation through either endoreplication or cell fusion-independent karyogamy events. Cell fusion plays less significant roles during solo unisexual reproduction.

During *C*. *neoformans* and *C*. *deneoformans* bisexual reproduction, the involvement of *KAR5* in nuclear fusion revealed that karyogamy machinery takes place at different sexual development stages between these two closely related sister species. As reported by Ning and colleagues, the Kar5 protein belongs to a divergent nuclear fusion protein family [43]. Neither CnKar5 nor CdKar5 share sequence similarities outside of the conserved CRD domain with Kar5 proteins from other ascomycetous fungi. Interestingly, the CnKar5 and CdKar5 protein sequences share 85% identity, compared to the average of 93% identity for the 5569 orthologs shared by these two sister species [50]. This suggests that the *KAR5* gene has undergone more rapid divergent evolution. The divergence of these proteins may contribute to the mechanistic differences in the karyogamy machinery and may represent a barrier for inter-species nuclear fusion (Fig 9). Several diploid or aneuploid environmental and clinical hybrid isolates of the two *Cryptococcus* species have been reported, but the few that produce spores have a <10% germination rate [51]. Incompatibility in components of the karyogamy machinery may help to generate a physical barrier for mating and drive speciation events within the *Cryptococcus* species complex.

Although we validated the conserved roles for *PRM1* and *KAR5*, neither is the sole fusion protein for plasma membrane fusion or nuclear membrane fusion; and deletion of these two factors caused different impacts on bisexual cycles in *Cryptococcus* (Fig 9). In *C*. *neoformans*, Prm1 participates in cell-cell fusion during the initial mating process and mediates clamp cell-hyphal fusion, which is required for maintaining dikaryotic hyphal growth, and Kar5 functions in karyogamy inside the basidia during bisexual reproduction. whereas, in *C*. *deneoformans*, Prm1 plays a more significant role in cell-cell fusion, and Kar5 can function in karyogamy immediately following cell fusion, which produces monokaryotic diploid hyphae (Fig 9). However, the observed monokaryotic hyphae could be derived from unisexual reproduction, as pheromone produced by cells of the opposite mating type can promote unisexual reproduction [7]. To address this, we used GFP- and mCherry-labeled nuclear markers to show that the nuclei inside of the monokaryotic hyphae are indeed karyogamy products labeled with both fluorescent markers and thus the products of bisexual reproduction (Fig 6). Collectively, these results demonstrated that there are major differences in both the cell fusion machinery and the karyogamy program during bisexual reproduction between these two closely related sister species (Fig 9).

In contrast to bisexual reproduction, deletion of *PRM1* did not cause a significant phenotypic defect during solo unisexual reproduction in *C*. *deneoformans*. Although *PRM1* was highly upregulated during the unisexual cycle, α-α cell fusion occurred at a 1000-fold lower frequency compared to **a**-α cell fusion. Furthermore, live cell imaging of yeast cell germination during unisexual reproduction provided compelling evidence that the yeast-hyphal morphological transition is largely independent of cell-cell fusion. It is likely *PRM1* may be a fortuitous transcriptional target during unisexual reproduction. However, it is worth noting that those cells that undergo cell-cell fusion do complete the unisexual cycle follow a pathway similar to the bisexual mating mechanism in *C*. *deneoformans*, and both *PRM1* and *KAR5* mediate cell-cell and nuclear fusion during modes of unisexual reproduction that results from α-α cell fusion as detected with genetically marked strains.

In bisexual reproduction, pheromone expression is dampened by the formation of the transcription factor complex Sxi1α-Sxi2**a** after **a**-α cell fusion [52]. Interestingly, pheromone expression was also dampened quickly during unisexual reproduction, but the transcriptional downregulation trigger must differ from bisexual reproduction because the opposite mating type was absent. During bisexual reproduction in both *C*. *neoformans* and *C*. *deneoformans*, *KAR5* expression was upregulated and dampened by *PRM1* deletion. *KAR5* expression was maintained at a basal level and was not affected by the deletion of *PRM1* during unisexual reproduction. Furthermore, deletion of *KAR5* did not change basidia nuclear morphology compared to wild type, demonstrating that *KAR5* is not required for unisexual reproduction. The fact that wild type, the *kar5* mutant, and the *kar7* mutant produced very few basidia with the two nuclei, indicating either that karyogamy does not occur inside the basidia during unisexual reproduction or that karyogamy occurs transiently and it is hard to capture by DAPI staining or nucleolar fluorescent marker Nop1-GFP. Interestingly, FACS analyses showed that the majority of blastospores produced along the hyphae from unisexual reproduction were diploid, supporting the hypothesis that nuclear fusion does not occur inside the basidium. Despite the fact that deletion of *KAR5* does not impact unisexual reproduction and nuclear fusion does not occur inside basidium, we can not entirely rule out that karyogamy could occur during unisexual reproduction, as deletion of *KAR5* did not completely block karyogamy during bisexual reproduction, and karyogamy genes in *Cryptococcus* share redundant functions [38]. Karyogamy occurs early during *C*. *deneoformans* bisexual reproduction, and it could also occur early in mother and daughter cells or growing hyphae, which leads to ploidy duplication. However, we favor the interpretation that karyogamy is dispensable for solo unisexual reproduction and an endoreplication pathway, which has been implicated in the formation of polyploid titan cells during *Cryptococcus* animal infection, contributes to ploidy duplication [53, 54] (Fig 9), which must be differentially controlled compared to titan cell formation, as titan cells reach a much higher ploidy [53].

With the ability to undergo both unisexual and bisexual reproduction, *Cryptococcus* serves as a model system to study the mating mechanisms for different sexual cycles. Our findings reveal the evolutionary differences in bisexual reproduction within the *Cryptococcus* species complex and suggest that the unisexual mating mechanism is plastic and complex, providing mechanistic insights to studies of mating mechanisms of unisexual reproduction and parthenogenesis in other eukaryotic systems.

## Materials and methods

### Strains, media, and growth conditions

Strains and plasmids used in this study are listed in S1 Table. All strains used to study bisexual reproduction in *C*. *neoformans* were generated in the congenic *MAT*α H99 and *MAT***a** KN99 strain backgrounds [33]. All strains used to study bisexual reproduction in *C*. *deneoformans* were generated in the congenic *MAT*α JEC21 and *MAT***a** JEC20 strain backgrounds [55]. All strains used to study unisexual reproduction in *C*. *deneoformans* were generated in the *MAT*α XL280 strain background [7]. Yeast cells were grown at 30°C on Yeast extract Peptone Dextrose (YPD) medium. Strains harboring dominant selectable markers were grown on YPD medium supplemented with nourseothricin (NAT) or G418 (NEO). Mating assays were performed on either 5% V8 juice agar medium (pH = 5.0 for *C*. *neoformans* and pH = 7.0 for *C*. *deneoformans*) or Murashige and Skoog (MS) medium minus sucrose (Sigma-Aldrich) in the dark at room temperature for the designated time period.

### Bioinformatics and phylogenetic analysis

To identify the *PRM1* orthologs in *C*. *neoformans* and *C*. *deneoformans*, BLASTP searches using the *S*. *cerevisiae*, *S*. *pombe*, *C*. *albicans*, *N*. *crassa*, and *A*. *fumigatus* Prm1 protein sequences were conducted against *C*. *neoformans* H99 and *C*. *deneoformans* JEC21 genomes on FungiDB (www.fungidb.org) [56]. This approach identified CNAG_05866 in *C*. *neoformans* and CNF01070 for *C*. *deneoformans* as candidate *PRM1* genes. Reciprocal BLAST searches confirmed that these two genes are *PRM1* orthologs in *Cryptococcus* spp. Phobius prediction suggested that both CdPrm1 and CnPrm1 have four transmembrane domains at the same amino acid positions (67–87, 352–371, 433–455, and 647–688) [44].

To identify the *KAR5* othologs in *C*. *neoformans* and *C*. *deneoformans*, a BLASTP search using the *P*. *graminis* Kar5 protein sequence against the *C*. *neoformans* H99 genome identified CNAG_04850 as a candidate *KAR5* gene for *C*. *neoformans*. However, the same BLASTP search failed to identify a candidate *KAR5* gene for *C*. *deneoformans*. A subsequent BLASTP search using the *C*. *neoformans KAR5* gene sequence against the *C*. *deneoformans* JEC21 genome identified a region from bp 790071 to 792560 on chromosome 10 encoding a protein that shares 85% identity with the *C*. *neoformans* candidate Kar5 protein sequence. Multiple sequence alignment of candidate *Cryptococcus* Kar5 protein sequences with predicted Kar5 protein sequences from other fungal species using the MUSCLE program confirmed they contain Cysteine Rich Domain (CRD) [43, 57]. Phylogenetic analyses for Prm1 and Kar5 were tested with 1000 bootstrap replicas by using the maximum likelihood method in MEGA7 [58, 59]. Phobius prediction predicted that both CdKar5 and CnKar5 have an N-terminal signal peptide and a C-terminal transmembrane domain at amino acid positions 1–16 and 476–501 for CdKar5, and 1–21 and 477–502 for CnKar5 [44]. The COILS/PCOILS program predicted that CdKar5 has four coiled-coil domains at amino acid positions 179–199, 216–236, 318–339, and 368–389, and that CnKar5 has two coiled-coil domains at amino acid positions 180–200 and 370–390 [45].

### Gene disruption and fluorescent protein expression

S1 Table and S2 Table lists the plasmids and primers, respectively, used in this study. To generate deletion mutants for genes of interest, deletion constructs consisting of the 5’ and 3’ regions of the targeted genes flanking an appropriate selection marker (*NAT* or *NEO* cassette) were generated by overlap PCR as previously described [60]. The deletion constructs were introduced into the respective strains via biolistic transformation as previously described [61]. Stable transformants were selected on YPD medium supplemented with NAT (100 mg/L) or G418 (200 mg/L). Gene replacements by homologous recombination were confirmed by PCR and Southern hybridization. To generate *C*. *deneoformans* wild type strains with dominant selectable markers for cell fusion assays, an analogous method was used to insert a dominant selectable marker (*NAT* cassette) into the intergenic region immediately downstream of the *URA5* gene (CNG03730) and a dominant selectable marker (*NEO* cassette) into the intergenic region between CNE02520 and CNE02530, which is downstream of the *ADE2* gene (CNE02500).

To visualize the cytosol in *Cryptococcus*, a plasmid encoding the cytosolic mCherry gene and containing a dominant selectable marker (*NEO* cassette) was generated. The mCherry coding sequence was amplified from pLKB25 [62] and inserted into pXL1 after the *GPD1* promoter using the Gibson assembly method, which assembles multiple DNA fragments with 20 to 40 bp overlap sequences in a single reaction containing exonuclease, DNA polymerase, and ligase [63], resulting in pCF1. To monitor nuclear morphology and dynamics during *Cryptococcus* sexual reproduction, plasmid pSL04 encoding a GFP-tagged nucleolar protein Nop1 from a previous study [38] and a plasmid encoding an mCherry-tagged histone H3 were used. To express the H3-mCherry chimera, the 1075-bp 5’UTR and the 683-bp 3’UTR of the H3 gene were used as promoter (P) and terminator (T), respectively. The H3 promoter and coding sequences before the stop codon and the H3 terminator sequence were amplified from JEC21α genomic DNA, and the mCherry coding sequence was inserted between the H3 coding sequence and H3 terminator by overlap PCR. The chimera expression cassette H3P-H3-mCherry-H3T was then inserted into pAI3 using the Gibson assembly method [63], resulting in pCF9. *C*. *deneoformans* strains were biolistically transformed with the pCF1, pSL04, and pCF9 plasmids, and the fluorescent protein expression cassettes were randomly inserted into the genomes. Stable transformants were screened based on fluorescent signals and the selectable markers.

### Cell-cell fusion assay

In *C*. *neoformans* bisexual reproduction, YSB119 (H99α *aca1*Δ::*NAT ura5 ACA1-URA5*) and YSB121 (KN99**a** *aca1*Δ::*NEO ura5 ACA1-URA5*) were used as genetically marked wild type strains to study the fusion competency of *prm1* (CF56 and CF562) and *kar5* (CF57 and CF549) mutants. In *C*. *deneoformans* bisexual reproduction, CF757 (JEC20**a** *URA5-NAT*) and CF762 (JEC21α *ADE2-NEO*) were used as wild type strains to study the fusion competency of *prm1* (CF1 and CF313) and *kar5* (CF487 and CF364) mutants. In*C*. *deneoformans* unisexual reproduction, CF750 (XL280α *URA5-NAT*) and CF752 (XL280α *ADE2-NEO*) were used as wild type strains to study the fusion competency of *prm1* (CF317 and CF659) and *kar5* (CF150 and CF260) mutants. Strains for each fusion pair were grown overnight in YPD liquid medium at 30°C. Cells were washed twice with ddH_2_O and diluted to a final density of OD_600_ = 2. Then, 50 μl of equal-volume mixed cells were spotted on V8 medium and incubated for 48 hours (for bisexual reproduction) or 72 hours (for unisexual reproduction) in the dark at room temperature. The cells were then removed, washed with ddH_2_O, and plated in serial dilution on both YPD medium and YPD medium supplemented with both NAT and G418. The cells were incubated for five days at room temperature. Cell-cell fusion frequency was measured by counting the average number of double drug resistant cfu/total cfu.

To quantify the cell-cell fusion frequency during *C*. *deneoformans* bisexual reproduction based on fluorescent signal mixing, CF830 (JEC21α *NOP1-GFP-NAT*) was mated with JEC20**a** for wild type fusion frequency, CF768 (JEC20**a** *prm1*Δ::*NEO NOP1-GFP-NAT*) was mated with either JEC21α for *prm1* mutant unilateral cell fusion frequency or with CF1 (JEC21α *prm1Δ*::*NEO*) for *prm1* mutant bilateral cell fusion frequency, and CF723 (JEC20**a** *kar5Δ*::*NEO NOP1-GFP-NAT*) was mated with CF487 (JEC21α *kar5Δ*::*NEO*) for *kar5* mutant bilateral cell fusion frequency. Cells were prepared as described above and collected for direct fluorescence microscopic observation after 24 hours of incubation. Approximately 100 fusion events were recorded for each mating and were identified by the presence of conjugation tubes connecting the fusion pairs. Fusion frequency was determined by the number of fusion pairs with Nop1-GFP labeled nuclei in both cellular compartments/total fusion events.

### Percoll gradient purification of spores

To determine whether *prm1* and *kar5* mutants were defective in spore production, spores were isolated by Percoll gradient centrifugation as previously described [64]. For *C*. *neoformans* bisexual reproduction, CF56 (H99α *prm1Δ*::*NAT*) crossed with CF562 (KN99**a** *prm1*Δ::*NEO*) and CF57 (H99α *kar5*Δ::*NAT*) crossed with CF549 (KN99**a** *kar5*Δ::*NEO*) were compared to the wild type cross between H99α and KN99**a**. For *C*. *deneoformans* bisexual reproduction, CF1 (JEC21α *prm1*Δ::*NEO*) crossed with CF313 (JEC20**a** *prm1*Δ::*NAT*) and CF487 (JEC21α *kar5*Δ::*NEO*) crossed with CF364 (JEC20**a** *kar5*Δ::*NAT*) were compared to wild type cross between JEC21α and JEC20**a**. For *C*. *deneoformans* unisexual mating, CF317 (XL280α *prm1*Δ::*NEO*) and CF260 (XL280α *kar5*Δ::*NEO*) were compared to the wild type XL280α. For each mating, triplicates were performed for statistical analysis. Strains were grown overnight in YPD liquid medium. Cells were washed twice with ddH_2_O and diluted to a final cell density of OD_600_ = 0.5. Then, 10 μl of equal-volume mixed cells were spotted on V8 medium and incubated for seven days in the dark at room temperature. The entire mating patch was suspended in 60% Percoll (GE Health) in PBS with 0.1% Triton X100. After centrifugation at 10,000 X *g* for 30 mins in an SW41Ti ultracentrifuge rotor (Beckman-Coulter), a band of spores near the bottom of the Percoll gradient was recovered with a 1-ml tuberculin syringe and transferred into an Eppendorf tube. The total spore production was determined by multiplying the spore density, measured by hemocytometer, with the final volume.

Wild type matings between CF757 (JEC20**a** *URA5-NAT*) and CF762 (JEC21α *ADE2-NEO*) were conducted as controls. The isolated cells were serially diluted and plated on YPD medium and allowed to recover for five days at 30°C. A total of 47 colonies were randomly chosen and grown on YPD medium supplemented with either NAT or G418 to assess growth phenotypes (S2 Fig). Mating type specific primer pairs were used to determine the *MAT* locus for the progeny.

### RNA extraction and RT-PCR

For all three modes of sexual reproduction studied, *prm1* and *kar5* mutant strains and wild type strains were grown overnight in YPD liquid medium. Cells were washed twice with ddH_2_O and diluted to OD_600_ = 2. Then 250 Δl of an equal-volume mixture of cells were spotted on V8 medium or YPD medium and incubated for 36 hours (YPD and V8) or one week (V8), as the pheromone pathway has been shown to be upregulated upon mating induction on V8 medium and the expression levels are maintained at relatively high levels between 24 and 48 hours [8]. Mating patches were harvested and flash frozen in liquid nitrogen. RNA was extracted using TRIzol reagent (Thermo) following the manufacturer’s instructions. RNA was treated with Turbo DNAse (Ambion), and single-stranded cDNA was synthesized by AffinityScript RT-RNAse (Stratagene). For each sample, cDNA synthesized without the RT/RNAse block enzyme mixture was used as a “no RT control” to control for genomic DNA contamination. The relative expression level of target genes was measured by quantitative real-time PCR using Brilliant III ultra-fast SYBR green QPCR mix (Stratagene) in an Applied Biosystems 7500 Real-Time PCR System. For each target, a “no template control” was performed to analyze melting curves to exclude primer artifacts. Technical triplicates and biological triplicates were performed for each sample. Gene expression levels were normalized using the endogenous reference gene *GPD1* and determined by using the comparative ΔΔCt method. The primers used for RT-PCR are listed in S2 Table. The Student’s t-test was used to determine if the relative gene expression levels between different strains exhibited statistically significant differences (*P* <0.05).

### Nuclear and plasma membrane staining

To visualize the nuclei during sexual reproduction, cells were stained with DAPI as previously described [62]. In brief, a 1-mm^3^ MS agar block containing hyphae on the edge of mating patches was excised and transferred to a small petri dish. The agar block was fixed in 3.7% formaldehyde and permeabilized in 1% Triton X100. The agar block was stained with 2 Δg/ml DAPI (Sigma) and transferred to a glass slide and covered with a cover slip for fluorescent microscopic observation.

To visualize the plasma membrane of the conjugation tubes during *C*. *deneoformans prm1* mutant bisexual reproduction, strain CF1 (JEC21α *prm1Δ*::*NEO*) was crossed with CF768 (JEC20**a** *prm1*Δ::*NEO NOP1-GFP-NAT*). After incubation on V8 medium for 24 hours, cells were harvested and resuspended in cold YPD liquid medium on ice. FM4-64 (Thermo) was added at a final concentration of 10 μM and the cells were stained on ice for 15 mins. The cells were then washed with cold YPD medium and fixed in 3.7% formaldehyde in PBS for 10 mins. After a final wash with PBS, the stained cells were examined immediately by confocal microscopy.

### Microscopy

Hyphal growth on the edge of mating patches, basidia, and spore chains were captured using a Nikon Eclipse E400 microscope equipped with a Nikon DXM1200F camera.

For fluorescence imaging of hyphae, an agar block supporting hyphal growth was excised and transferred onto a glass slide and covered with a coverslip. For fluorescence imaging of short early hyphae and fusion pairs, early mating patches were harvested and suspended in ddH_2_O and cells were placed on a glass slide containing a 2% agar patch and covered with a coverslip. Fluorescent images were obtained using a Deltavision system (Olympus IX-71 base) equipped with a Coolsnap HQ2 high resolution SSD camera. Images were processed using the software FIJI.

Confocal fluorescent images were captured by confocal laser scanning microscopy using a Zeiss LSM 710 Confocal Microscope at the Duke Light Microscopy Core Facility. Plan-Apochromat 63X/1.40 Oil DIC M27 objective lenses were used for imaging, and a smart setup was used for image acquisition configuration. Confocal fluorescent images and movies were processed using the ZEN software.

SEM and TEM were performed at the North Carolina State University Center for Electron Microscopy, Raleigh, NC, USA. Samples were prepared for SEM as previously described [8]. In brief, 1-mm^3^ MS agar blocks containing hyphae on the edge of mating patches were excised and fixed in 0.1 M sodium cacodylate buffer, pH = 6.8, containing 3% glutaraldehyde at 4°C for several weeks. Before viewing, the agar block was rinsed with cold 0.1 M sodium cacodylate buffer, pH = 6.8 three times and post-fixed in 2% osmium tetroxide in cold 0.1 M cacodylate buffer, pH = 6.8 for 2.5 hours at 4°C. Then the block was critical-point dried with liquid CO_2_ and sputter coated with 50 Å of gold/palladium using a Hummer 6.2 sputter coater (Anatech). The samples were viewed at 15KV with a JSM 5900LV scanning electron microscope (JEOL) and captured with a Digital Scan Generator (JEOL) image acquisition system. For TEM, conjugation tubes were prepared by crossing strain CF712 (JEC21α *prm1*Δ::*NAT mCherry-NEO*) with CF768 (JEC20**a** *prm1*Δ::*NEO NOP1-GFP-NAT*). After incubation on V8 medium for 24 hours, cells were harvested and analyzed with a B-C Astrios Sorter to enrich fusion pairs that were positive for both GFP and mCherry fluorescence at the Duke Cancer Institute Flow Cytometry Shared Resource. Hyphae were prepared by crossing strain CF56 (H99α *prm1*Δ::*NAT*) with CF562 (KN99**a** *prm1*Δ::*NEO*). After incubation on V8 medium for four weeks, hyphae on the edge of the mating patches were harvested for observation of clamp cell morphology. Upon harvest, cells or hyphae were immediately fixed in 3% glutaraldehyde in 0.1 M sodium cacodylate buffer, pH = 6.8, at 4°C for several weeks. The sample preparation was performed as previously described [62]. In brief, cells were post-fixed with 4% KMnO_4_ and pre-embedded in 2% agarose. After dehydration with an increasing gradient of ethanol solutions and filtration with Spurr’s resin, the agarose block was embedded in 100% Spurr’s in BEEF capsules. Thin sections were cut and collected on 200-mesh grids, followed by staining with 4% aqueous uranyl acetate and Reynold’s lead citrate. Grids were viewed using a Philips 400T transmission electron microscope. TEM images were processed with Photoshop (Adobe).

### Flow cytometry

Ploidy of blastospores was determined by Fluorescence Activated Cell Sorting (FACS) analysis as previously described [65]. XL280α and MN142.6 (XL280α/α *ura5*Δ::*NAT*/*ura5*Δ::*NEO*) were used as haploid and diploid controls respectively. Dissected blastospores were grown on YPD medium between three and five days at 30°C to yield colonies. Cells were harvested and washed with PBS buffer. After fixation in 70% ethanol at 4°C overnight, cells were washed once with 1 ml of NS buffer (10 mM Tris-HCl, pH = 7.2, 250 mM sucrose, 1 mM EDTA, pH = 8.0, 1 mM MgCl_2_, 0.1 mM CaCl_2_, 0.1 mM ZnCl_2_, 0.4 mM phenylmethylsulfonyl fluoride, and 7 mM β-mercaptoethanol), and stained in 180 μl NS buffer with 20 μl 10 mg/ml RNase and 5 μl 0.5 mg/ml propidium iodide at 4°C overnight. Then, 50 μl stained cells were diluted in 2 ml of 50 mM Tris-HCl, pH = 8.0 and sonicated for 1 min. For each sample, 10,000 cells were analyzed on the FL1 channel on the Becton-Dickinson FACScan at Duke Cancer Institute Flow Cytometry Shared Resource. Data analysis was performed using the software FlowJo.

## Supporting information

S1 Fig Identification of the *PRM1* and *KAR5* genes in *C*. *neoformans* and *C*. *deneoformans*. **(A)** We identified *PRM1* homologs for *C*. *neoformans* (CNAG_05866) and *C*. *deneoformans* (CNF01070) using BLASTP searches of Prm1 protein sequences from *S*. *cerevisiae*, *S*. *pombe*, *C*. *albicans*, *N*. *crassa*, and *A*. *fumigatus* against the *C*. *neoformans* and *C*. *deneoformans* protein databases. BLASTP and reciprocal BLASTP E-values for CNAG_05866 are listed. **(B)** Phylogenetic analysis of Prm1 protein sequences based on the maximum likelihood method in MEGA7. The percentage of trees in which the associated taxa clustered together is shown at each split. Branch length indicates the number of substitutions per site. **(C)** Identification of the *KAR5* genes for *C*. *neoformans* (CNAG_04850) and *C*. *deneoformans* by BLASTP searches of Kar5 protein sequences from *S*. *cerevisiae*, *S*. *pombe*, *C*. *albicans*, *N*. *crassa*, *A*. *fumigatus*, and *P*. *graminis* against the *C*. *neoformans* and *C*. *deneoformans* protein databases. BLASTP and reciprocal BLASTP E-values for CNAG_04850 are listed. Only the *P*. *graminis* Kar5 protein sequence showed sequence similarity with *C*. *neoformans* and *C*. *deneoformans* Kar5 protein sequences. **(D)** Phylogenetic analysis of Kar5 protein sequences from eight fungal species based on the maximum likelihood method in MEGA7. Node and branch annotations for Kar5 tree are the same as for the Prm1 tree. **(E)** Multiple sequence alignment of Kar5 protein sequences using the MUSCLE alignment program revealed the conserved Cysteine Rich Domain (CRD) for the distantly related Kar5 proteins in the eight fungal species included.
(TIF)

S2 Fig Hyphal production for *prm1* and *kar5* mutants during *C*. *neoformans* bisexual reproduction. **(A)** A wild type cross between H99α and KN99**a** and two independent *prm1* bilateral mutant crosses (between CF30 and CF448, and between CF56 and CF562) were incubated on MS medium in the dark at room temperature for 10 days. **(B)** A wild type cross between H99α and KN99**a** and two independent *kar5* bilateral mutant crosses (between CF57 and CF549, and between CF208 and CF305) were incubated on MS medium in the dark at room temperature for 10 days. The scale bar is 100 μm.
(TIF)

S3 Fig Spore production for *prm1* and *kar5* mutants during all three *Cryptococcus* sexual cycles. **(A)** Relative spore production of *prm1* mutants and **(B)** Relative spore production of *kar5* mutants compared to wild type after 7-days incubation on V8 medium.
(TIF)

S4 Fig Validation of spores isolated by Percoll gradient centrifugation by genetic analysis. Spores from a wild type cross CF757 (JEC20**a** *URA5-NAT*) and CF762 (JEC21α *ADE2-NEO*) were isolated following seven days of co-incubation. To test recombination among F1 progeny, 47 spore-derived colonies were randomly chosen for phenotypic and genetic analysis. **(A)** The 47 progeny were grown on YPD medium supplemented with NAT or G418 to test for selectable marker inheritance. **(B)** Mating type for each progeny was determined by *MAT* locus specific primer sets. Genotypes for each progeny are provided in the grid in the same order as the progeny were grown on the YPD medium. **(C)** Parental and non-parental genotypes are summarized in the graphical table.
(TIF)

S5 Fig Plasma membrane structures of unfused clamp cells during *C*. *neoformans* bisexual reproduction as visualized by transmission electron microscopy. CF56 (H99α *prm1Δ*::*NAT*) was mated with CF562 (KN99**a** *prm1Δ*::*NEO*) on V8 medium for four weeks and hyphae on the edge of the mating patch were collected for TEM. **(A)** Cross section of a fused clamp cell morphology is provided on the left, and the diagram is shown on the right. The scale bar is 2 μm. **(B)** Plasma membrane structures at three unfused conjugation sites were further examined at higher magnification. The diagram for the clamp cell cross section is provided on the right. In the left panels, the scale bars are 2 μm, and in the middle panels, the scale bars are 0.5 μm.
(TIF)

S6 Fig Cell fusion frequency determined by fluorescent signal intermixing between fusion partners. Equal number of cells for each fusion pair were mixed and incubated on V8 medium for 24 hours. Cells were harvested and examined under fluorescent microscope to determine cell fusion frequency based on Nop1-GFP fluorescent signal intermixing between each fusion pair. **(A)** Wild type cell fusion between CF830 (JEC21α *NOP1-GFP-NAT*) and JEC20**a**. **(B)** Unilateral cell fusion between JEC21α and CF768 (JEC20**a** *prm1*Δ::*NEO NOP1-GFP-NAT*). **(C)** Bilateral mating between CF1 (JEC21α *prm1*Δ::*NEO*) and CF768 (JEC20**a** *prm1*Δ::*NEO NOP1-GFP-NAT*). **(D)** Bilateral mating between CF487 (JEC21α *kar5*Δ::*NEO*) and CF723 (JEC20**a** *kar5*Δ::*NEO NOP1-GFP-NAT*). Closed shapes (left panels) and arrows (right panels) indicate successful cell fusion pairs, whereas open shapes (left panels) and arrowheads (right panels) indicate unfused cell fusion pairs. The scale bars are 20 μm.
(TIF)

S7 Fig Defective cell fusion phenotypes during *C*. *deneoformans* bisexual reproduction. **(A)** Plasma membrane structures of unfused yeast cells during *C*. *deneoformans* bisexual reproduction as visualized by transmission electron microscopy. Unfused cell fusion pairs between CF712 (JEC21α *prm1*Δ::*NAT mCherry-NEO*) and CF768 (JEC20**a** *prm1*Δ::*NEO NOP1-GFP-NAT*) were examined by transmission electron microscopy. Plasma membrane structures at the conjugation sites were further examined at higher magnification. The diagram for the fusion pair cross section is provided on the right. For the left panels, the scale bars are 2 μm, and for the middle panels, the scale bars are 0.5 μm. **(B)** *prm1* mutants are defective in clamp cell fusion during *C*. *deneoformans* bisexual reproduction. SEM of the unfused clamp cell morphology for the wild type cross (JEC21α X JEC20**a**) and of the defective clamp cell fusion morphology for the *prm1* mutant cross (CF1 X CF313). The scale bar is 5 μm.
(TIF)

S8 Fig *C*. *deneoformans* produces monokaryotic hyphae during both unisexual and bisexual reproduction. **(A)** Wild type cross between JEC20**a** and JEC21α, *prm1* mutant cross between CF1 and CF313, and *kar5* mutant cross between CF226 and CF364 for bisexual reproduction, and **(B)** Wild type strain XL280α, *prm1* mutant CF659, and *kar5* mutant CF260 were incubated on V8 medium in the dark at room temperature for four weeks to generate hyphae and basidia from unisexual reproduction. DAPI staining showed wild type, *prm1* mutants, and *kar5* mutants all produced monokaryotic hyphae during both unisexual and bisexual reproduction. The scale bar is 5 μm.
(TIF)

S9 Fig Tracking hyphal nuclear morphology with GFP labeled nucleolar marker Nop1 protein for wild type, *prm1*, and *kar5* mutants during *C*. *deneoformans* bisexual reproduction. Wild type cross CF830 (JEC21α *NOP1-GFP-NAT*) X JEC20**a**, *prm1* bilateral mutant cross CF1 (JEC21α *prm1Δ*::*NEO*) X CF768 (JEC20**a** *prm1Δ*::*NEO NOP1-GFP-NAT*), and *kar5* bilateral mutant cross CF487 (JEC21α *kar5*Δ::*NEO*) X CF723 (JEC20**a** *kar5*Δ::*NAT NOP1-GFP-NAT*) were examined by direct fluorescence microscopy to track hyphal nuclear morphology at different stages of sexual reproduction. **(A)** At 48 hours, wild type and *prm1* mutants produced both monokaryotic and dikaryotic hyphae (arrows point to monokaryotic hyphae, and arrowhead points to mitotically dividing dikaryotic wild type hyphae). *kar5* mutants produce hyphae with two nuclei in close contact (arrows). **(B)** At 10 days, the wild type cross produced both monokaryotic and dikaryotic hyphae, *prm1* mutants mainly produced monokaryotic hyphae, and *kar5* mutants mainly produced dikaryotic hyphae. **(C)** At six weeks, wild type and *prm1* mutants mainly produced monokaryotic hyphae, and *kar5* mutants produced both monokaryotic and dikaryotic hyphae. The scale bar is 5 μm.
(TIF)

S10 Fig Nuclear morphology determined by DAPI staining during *C*. *deneoformans* unisexual reproduction for wild type, *kar5*, and *kar7* mutants. **(A)** Representative basidia containing one nucleus (left panel), or two nuclei (middle panel), or more than two nuclei (right panel) with DAPI staining are shown for wild type XL280α, *kar5* mutant (CF260), and *kar7* mutant (SL277). Arrows point to DAPI stained nuclei inside basidia. The scale bars are 5 μm. **(B)** Basidia containing one nucleus, or two nuclei, or more than four post-meiotic nuclei were quantified for the above strains.
(TIF)

S11 Fig Nuclear morphology determined by Nop1-GFP during *C*. *deneoformans* unisexual reproduction for wild type, *kar5*, and *kar7* mutants. **(A)** Representative basidia containing one nucleus (left panel), or two nuclei (middle panel), or more than two nuclei (right panel) with the nucleolar marker Nop1-GFP fluorescent signals are shown for wild type XL280α (CF836), *kar5* mutant (CF718), and *kar7* mutant (CF1442). Arrows point to Nop1-GFP signal inside basidia. The scale bars are 5 μm. **(B)** Basidia containing one nucleus, or two nuclei, or more than two nuclei were quantified for the above strains.
(TIF)

S12 Fig *prm1 spo11* and *kar5 spo11* double mutants exhibit a sporulation defect. **(A)** *prm1* mutant (CF317) and two independent *prm1 spo11* double mutants (CF894 and CF901), and **(B)** *kar5* mutant (CF260) and two independent *kar5 spo11* double mutants (CF883 and CF884) were incubated on V8 medium in the dark at room temperature for four weeks. *prm1 spo11* double mutants and *kar5 spo11* double mutants produced two spore chains compared to the four spore chains produced by *prm1* or *kar5* mutants. Schemes showing wild type and mutant sporulation patterns were provided at the lower left corner of each image. The scale bar equals 10 μm.
(TIF)

S13 Fig Ploidy determination by FACS for blastospores produced by *kar7* mutant during unisexual reproduction. The upper panels are FACS results for haploid control XL280α and diploid control MN142.6 α/α. The lower panels are representative FACS results for haploid, aneuploid, and diploid blastospores produced by the *kar7* mutant.
(TIF)

S1 Movie 3D animation of the fused cell sample in Fig 4A. CF712 (JEC21α *prm1*Δ::*NAT mCherry-NEO*) was mated with CF768 (JEC20**a** *prm1*Δ::*NEO NOP1-GFP-NAT*), and the cytosolic mCherry fluorescence signal and nucleolar marker Nop1-GFP signal were intermixed in the fusion pair.
(AVI)

S2 Movie 3D animation for the unfused cell sample in Fig 4A. CF712 (JEC21α *prm1*Δ::*NAT mCherry-NEO*) was mated with CF768 (JEC20**a** *prm1Δ*::*NEO NOP1-GFP-NAT*), and the cytosolic mCherry fluorescence signal and nucleolar marker Nop1-GFP signal were restricted to distinct cellular compartments.
(AVI)

S3 Movie Time-lapse confocal microscopy for hyphal initiation during *C*. *deneoformans* unisexual reproduction. Cells from CF836 (XL280α *NOP1-GFP-NAT*) and CF1091 (XL280α *H3-mCherry-NAT*) were mixed and co-incubated on V8 medium for 24 hours at room temperature. Early hyphae were examined for nuclear fluorescence under confocal microscope for 30 minutes.
(AVI)

S1 Table Strains and plasmids used in this study. (DOCX)

S2 Table Primers used in this study. (DOCX)

S3 Table Blastospores dissected in this study. (DOCX)
