# Supplementary figures and images for: PRM1 and KAR5 function in cell-cell fusion and karyogamy to drive distinct bisexual and unisexual cycles in the Cryptococcus pathogenic species complex
Source: PLoS Genet. 2017 Nov 27;13(11):e1007113. doi: 10.1371/journal.pgen.1007113 (PMC5720818; doi:10.1371/journal.pgen.1007113)

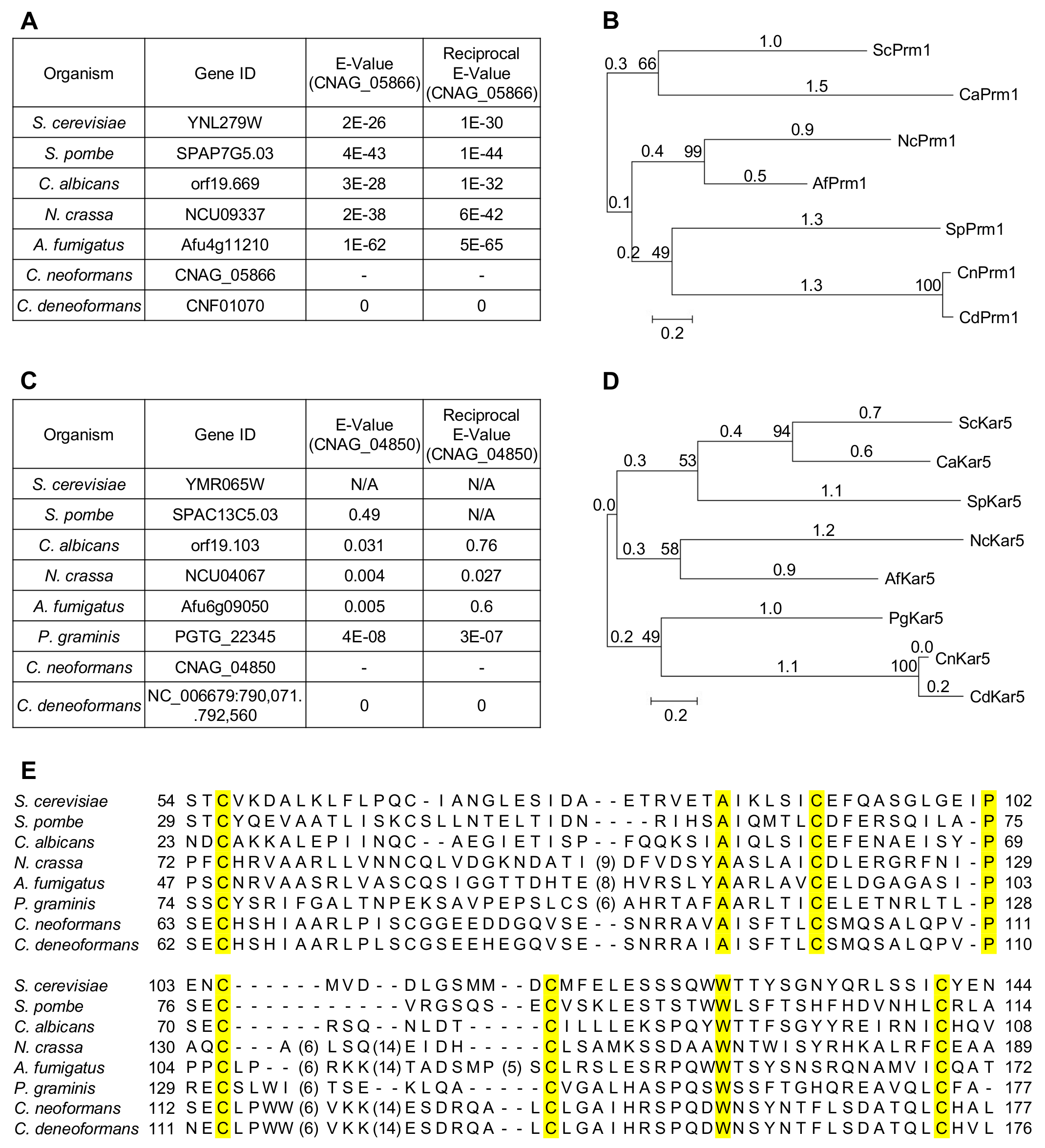

Supplement: S1 Fig — (A) We identified PRM1 homologs for C. neoformans (CNAG_05866) and C. deneoformans (CNF01070) using BLASTP searches of Prm1 protein sequences from S. cerevisiae, S. pombe, C. albicans, N. crassa, and A. fumigatus against the C. neoformans and C. deneoformans protein databases. BLASTP and reciprocal BLASTP E-values for CNAG_05866 are listed. (B) Phylogenetic analysis of Prm1 protein sequences based on the maximum likelihood method in MEGA7. The percentage of trees in which the associated taxa clustered together is shown at each split. Branch length indicates the number of substitutions per site. (C) Identification of the KAR5 genes for C. neoformans (CNAG_04850) and C. deneoformans by BLASTP searches of Kar5 protein sequences from S. cerevisiae, S. pombe, C. albicans, N. crassa, A. fumigatus, and P. graminis against the C. neoformans and C. deneoformans protein databases. BLASTP and reciprocal BLASTP E-values for CNAG_04850 are listed. Only the P. graminis Kar5 protein sequence showed sequence similarity with C. neoformans and C. deneoformans Kar5 protein sequences. (D) Phylogenetic analysis of Kar5 protein sequences from eight fungal species based on the maximum likelihood method in MEGA7. Node and branch annotations for Kar5 tree are the same as for the Prm1 tree. (E) Multiple sequence alignment of Kar5 protein sequences using the MUSCLE alignment program revealed the conserved Cysteine Rich Domain (CRD) for the distantly related Kar5 proteins in the eight fungal species included. (TIF) [file pgen.1007113.s001.tif]

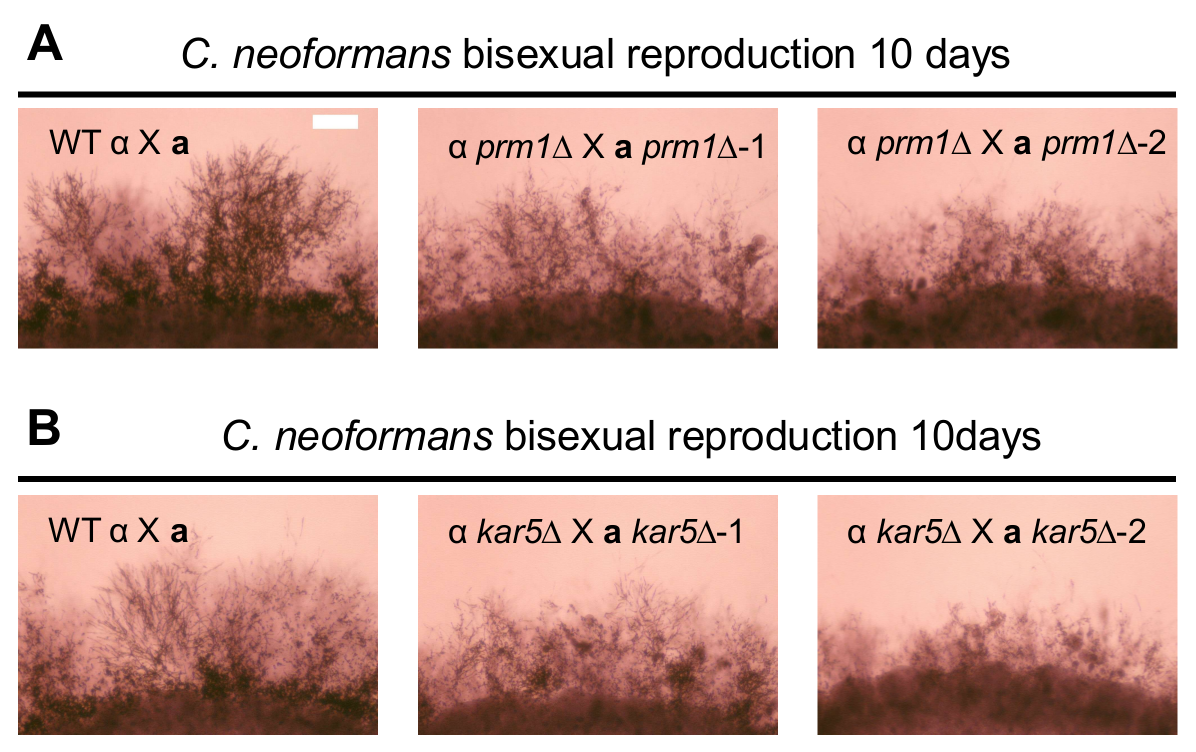

Supplement: S2 Fig — (A) A wild type cross between H99α and KN99a and two independent prm1 bilateral mutant crosses (between CF30 and CF448, and between CF56 and CF562) were incubated on MS medium in the dark at room temperature for 10 days. (B) A wild type cross between H99α and KN99a and two independent kar5 bilateral mutant crosses (between CF57 and CF549, and between CF208 and CF305) were incubated on MS medium in the dark at room temperature for 10 days. The scale bar is 100 μm. (TIF) [file pgen.1007113.s002.tif]

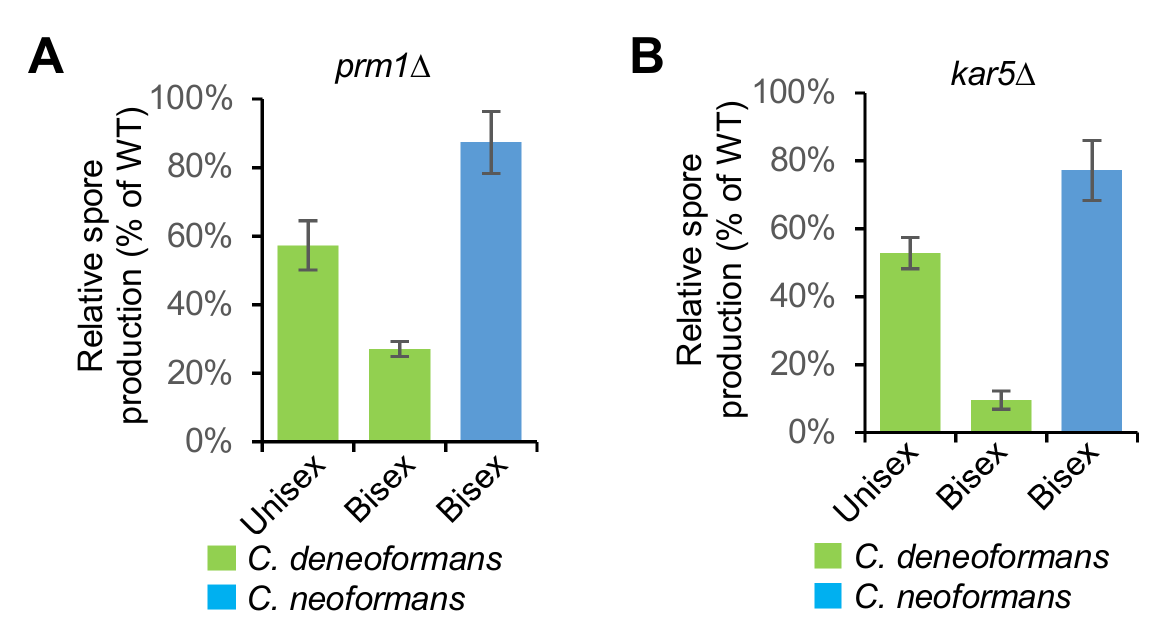

Supplement: S3 Fig — (A) Relative spore production of prm1 mutants and (B) Relative spore production of kar5 mutants compared to wild type after 7-days incubation on V8 medium. (TIF) [file pgen.1007113.s003.tif]

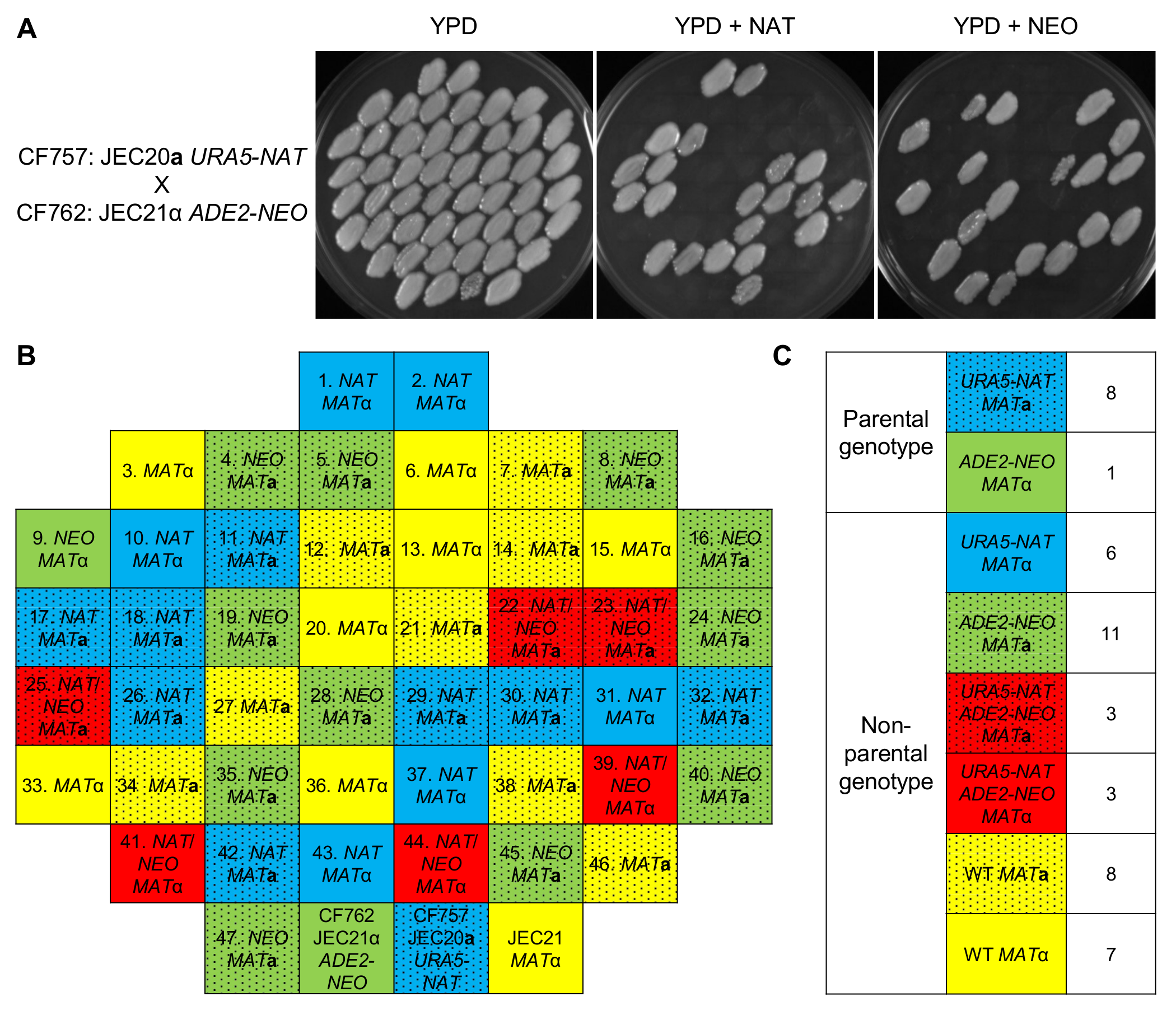

Supplement: S4 Fig — Spores from a wild type cross CF757 (JEC20a URA5-NAT) and CF762 (JEC21α ADE2-NEO) were isolated following seven days of co-incubation. To test recombination among F1 progeny, 47 spore-derived colonies were randomly chosen for phenotypic and genetic analysis. (A) The 47 progeny were grown on YPD medium supplemented with NAT or G418 to test for selectable marker inheritance. (B) Mating type for each progeny was determined by MAT locus specific primer sets. Genotypes for each progeny are provided in the grid in the same order as the progeny were grown on the YPD medium. (C) Parental and non-parental genotypes are summarized in the graphical table. (TIF) [file pgen.1007113.s004.tif]

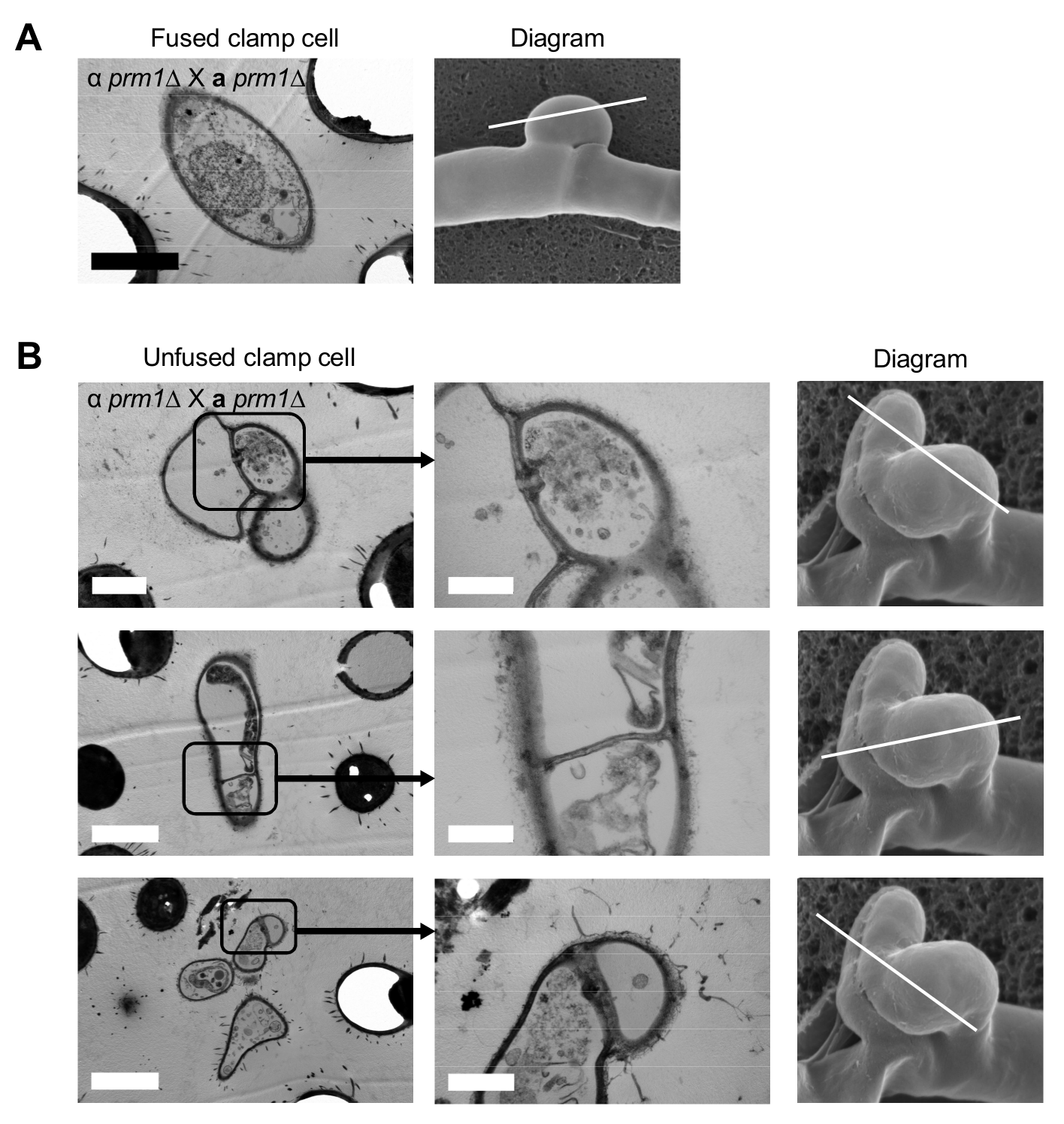

Supplement: S5 Fig — CF56 (H99α prm1Δ::NAT) was mated with CF562 (KN99a prm1Δ::NEO) on V8 medium for four weeks and hyphae on the edge of the mating patch were collected for TEM. (A) Cross section of a fused clamp cell morphology is provided on the left, and the diagram is shown on the right. The scale bar is 2 μm. (B) Plasma membrane structures at three unfused conjugation sites were further examined at higher magnification. The diagram for the clamp cell cross section is provided on the right. In the left panels, the scale bars are 2 μm, and in the middle panels, the scale bars are 0.5 μm. (TIF) [file pgen.1007113.s005.tif]

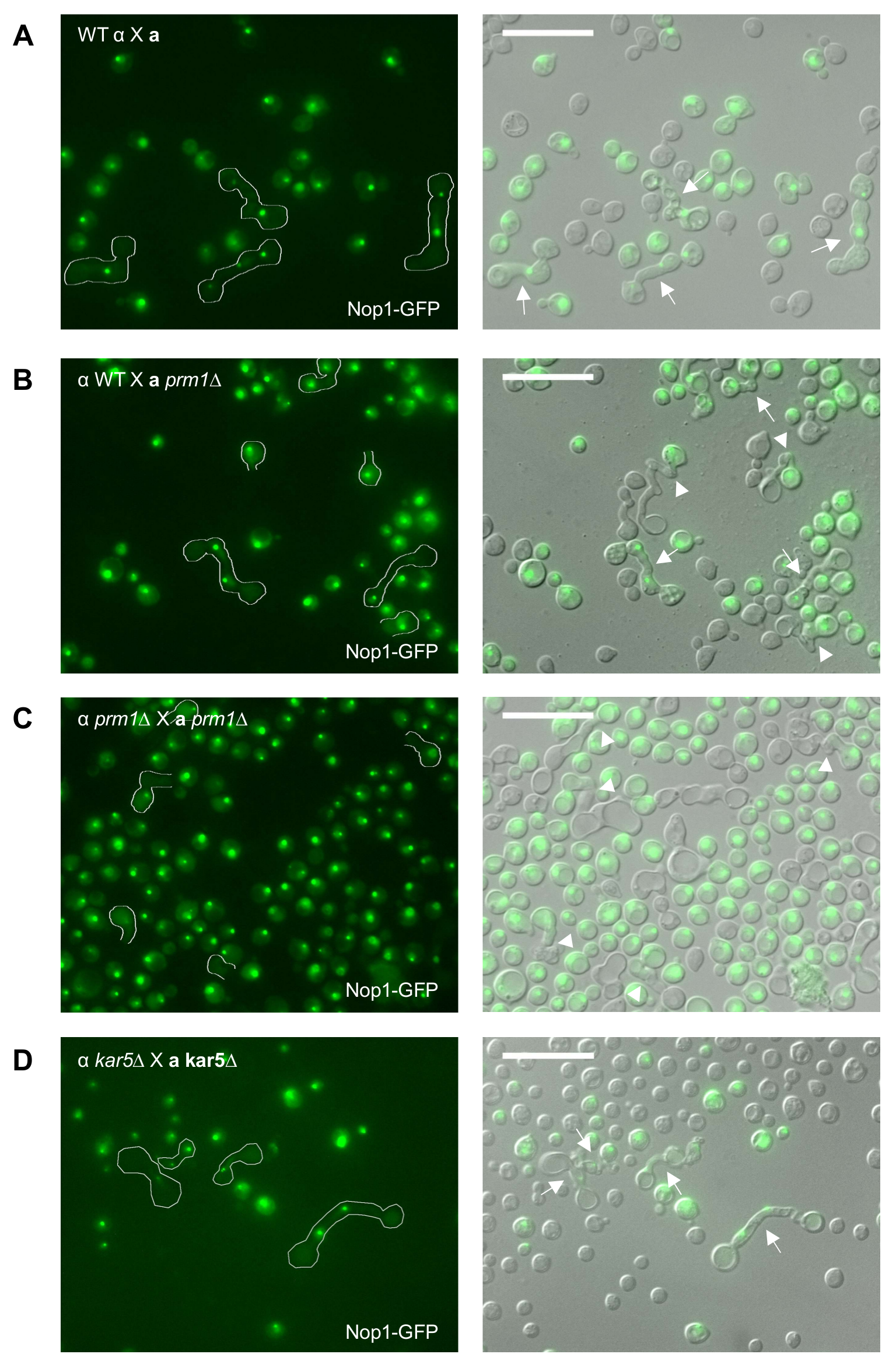

Supplement: S6 Fig — Equal number of cells for each fusion pair were mixed and incubated on V8 medium for 24 hours. Cells were harvested and examined under fluorescent microscope to determine cell fusion frequency based on Nop1-GFP fluorescent signal intermixing between each fusion pair. (A) Wild type cell fusion between CF830 (JEC21α NOP1-GFP-NAT) and JEC20a. (B) Unilateral cell fusion between JEC21α and CF768 (JEC20a prm1Δ::NEO NOP1-GFP-NAT). (C) Bilateral mating between CF1 (JEC21α prm1Δ::NEO) and CF768 (JEC20a prm1Δ::NEO NOP1-GFP-NAT). (D) Bilateral mating between CF487 (JEC21α kar5Δ::NEO) and CF723 (JEC20a kar5Δ::NEO NOP1-GFP-NAT). Closed shapes (left panels) and arrows (right panels) indicate successful cell fusion pairs, whereas open shapes (left panels) and arrowheads (right panels) indicate unfused cell fusion pairs. The scale bars are 20 μm. (TIF) [file pgen.1007113.s006.tif]

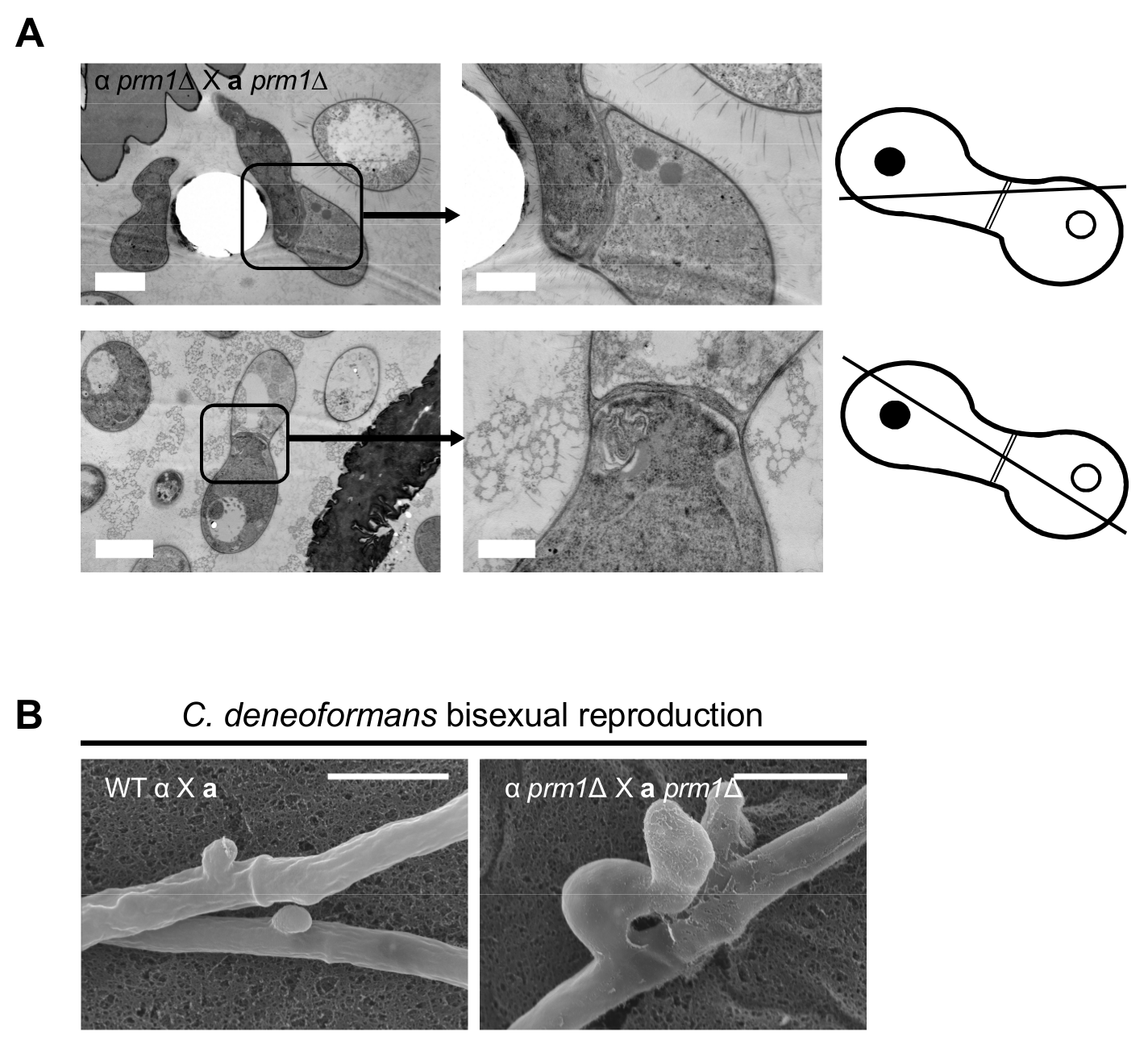

Supplement: S7 Fig — (A) Plasma membrane structures of unfused yeast cells during C. deneoformans bisexual reproduction as visualized by transmission electron microscopy. Unfused cell fusion pairs between CF712 (JEC21α prm1Δ::NAT mCherry-NEO) and CF768 (JEC20a prm1Δ::NEO NOP1-GFP-NAT) were examined by transmission electron microscopy. Plasma membrane structures at the conjugation sites were further examined at higher magnification. The diagram for the fusion pair cross section is provided on the right. For the left panels, the scale bars are 2 μm, and for the middle panels, the scale bars are 0.5 μm. (B) prm1 mutants are defective in clamp cell fusion during C. deneoformans bisexual reproduction. SEM of the unfused clamp cell morphology for the wild type cross (JEC21α X JEC20a) and of the defective clamp cell fusion morphology for the prm1 mutant cross (CF1 X CF313). The scale bar is 5 μm. (TIF) [file pgen.1007113.s007.tif]

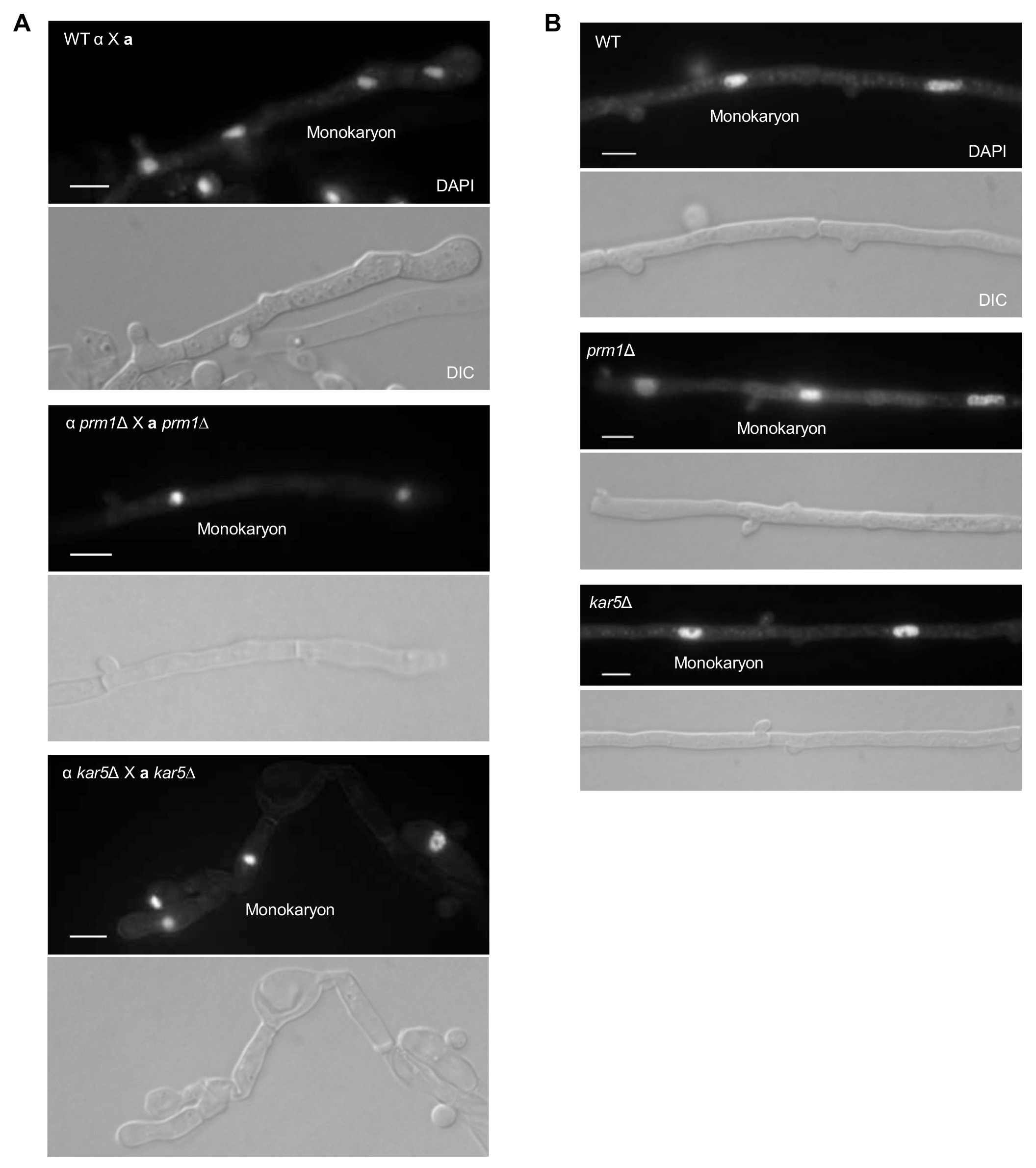

Supplement: S8 Fig — (A) Wild type cross between JEC20a and JEC21α, prm1 mutant cross between CF1 and CF313, and kar5 mutant cross between CF226 and CF364 for bisexual reproduction, and (B) Wild type strain XL280α, prm1 mutant CF659, and kar5 mutant CF260 were incubated on V8 medium in the dark at room temperature for four weeks to generate hyphae and basidia from unisexual reproduction. DAPI staining showed wild type, prm1 mutants, and kar5 mutants all produced monokaryotic hyphae during both unisexual and bisexual reproduction. The scale bar is 5 μm. (TIF) [file pgen.1007113.s008.tif]

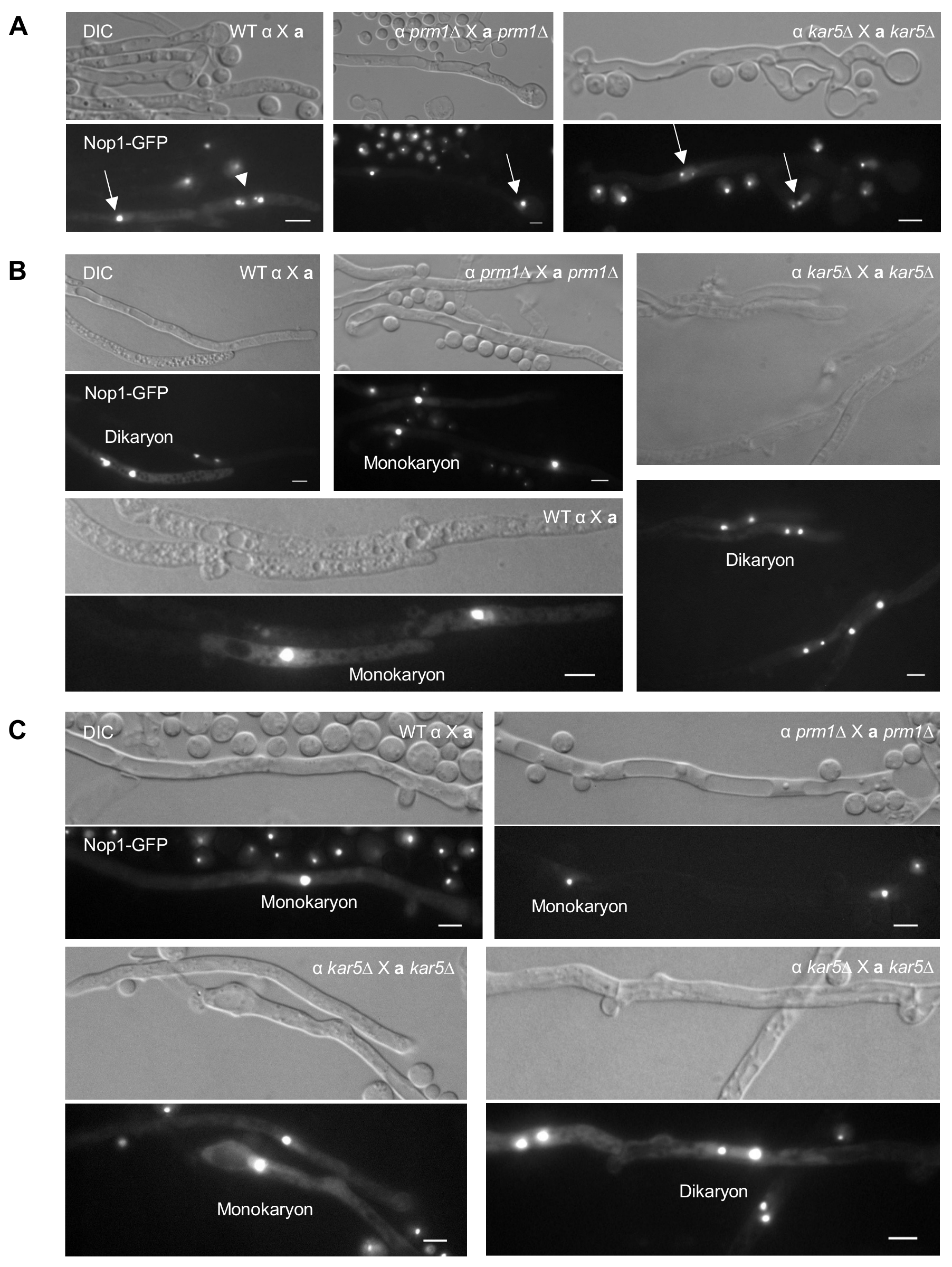

Supplement: S9 Fig — Wild type cross CF830 (JEC21α NOP1-GFP-NAT) X JEC20a, prm1 bilateral mutant cross CF1 (JEC21α prm1Δ::NEO) X CF768 (JEC20a prm1Δ::NEO NOP1-GFP-NAT), and kar5 bilateral mutant cross CF487 (JEC21α kar5Δ::NEO) X CF723 (JEC20a kar5Δ::NAT NOP1-GFP-NAT) were examined by direct fluorescence microscopy to track hyphal nuclear morphology at different stages of sexual reproduction. (A) At 48 hours, wild type and prm1 mutants produced both monokaryotic and dikaryotic hyphae (arrows point to monokaryotic hyphae, and arrowhead points to mitotically dividing dikaryotic wild type hyphae). kar5 mutants produce hyphae with two nuclei in close contact (arrows). (B) At 10 days, the wild type cross produced both monokaryotic and dikaryotic hyphae, prm1 mutants mainly produced monokaryotic hyphae, and kar5 mutants mainly produced dikaryotic hyphae. (C) At six weeks, wild type and prm1 mutants mainly produced monokaryotic hyphae, and kar5 mutants produced both monokaryotic and dikaryotic hyphae. The scale bar is 5 μm. (TIF) [file pgen.1007113.s009.tif]

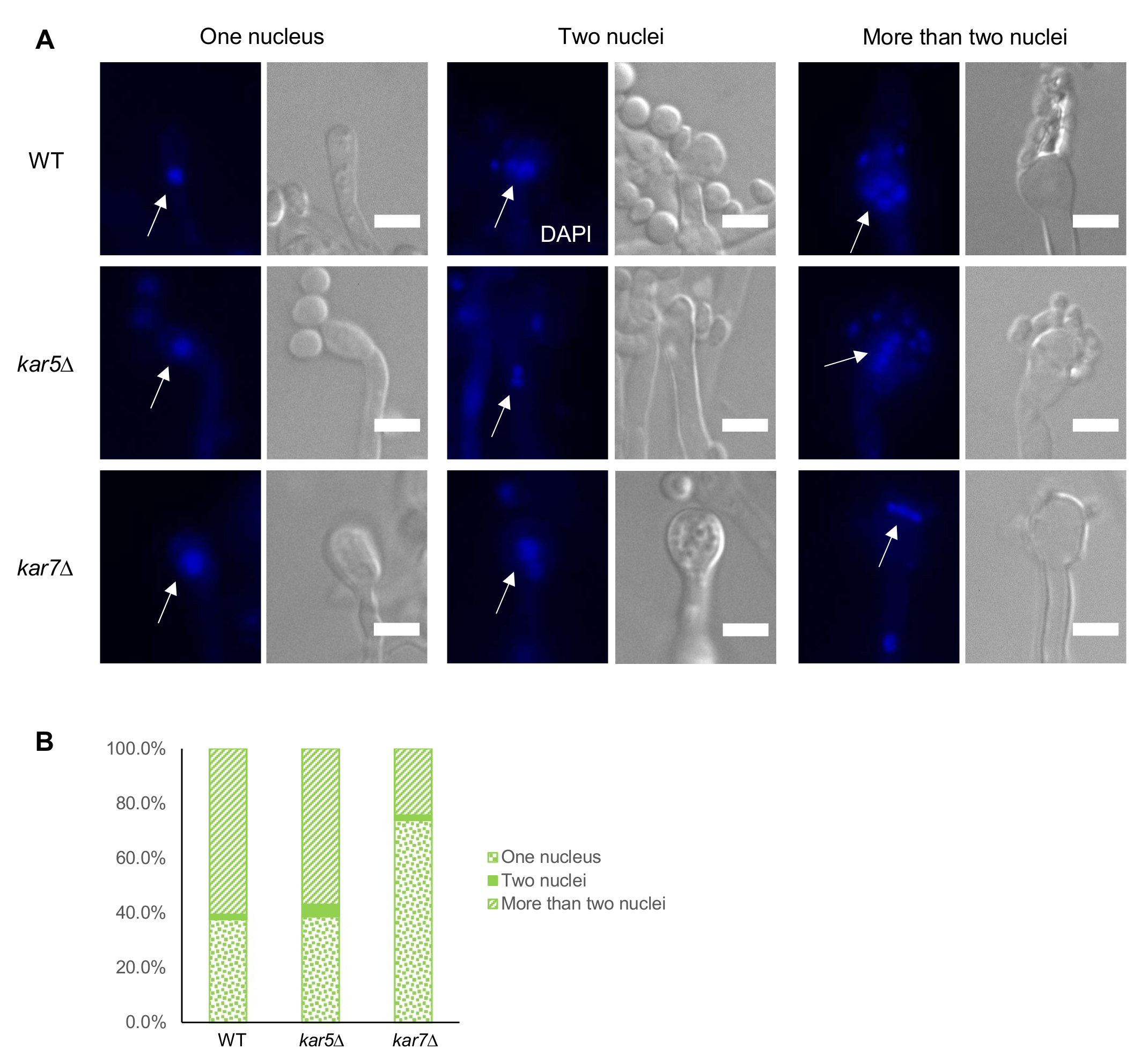

Supplement: S10 Fig — (A) Representative basidia containing one nucleus (left panel), or two nuclei (middle panel), or more than two nuclei (right panel) with DAPI staining are shown for wild type XL280α, kar5 mutant (CF260), and kar7 mutant (SL277). Arrows point to DAPI stained nuclei inside basidia. The scale bars are 5 μm. (B) Basidia containing one nucleus, or two nuclei, or more than four post-meiotic nuclei were quantified for the above strains. (TIF) [file pgen.1007113.s010.tif]

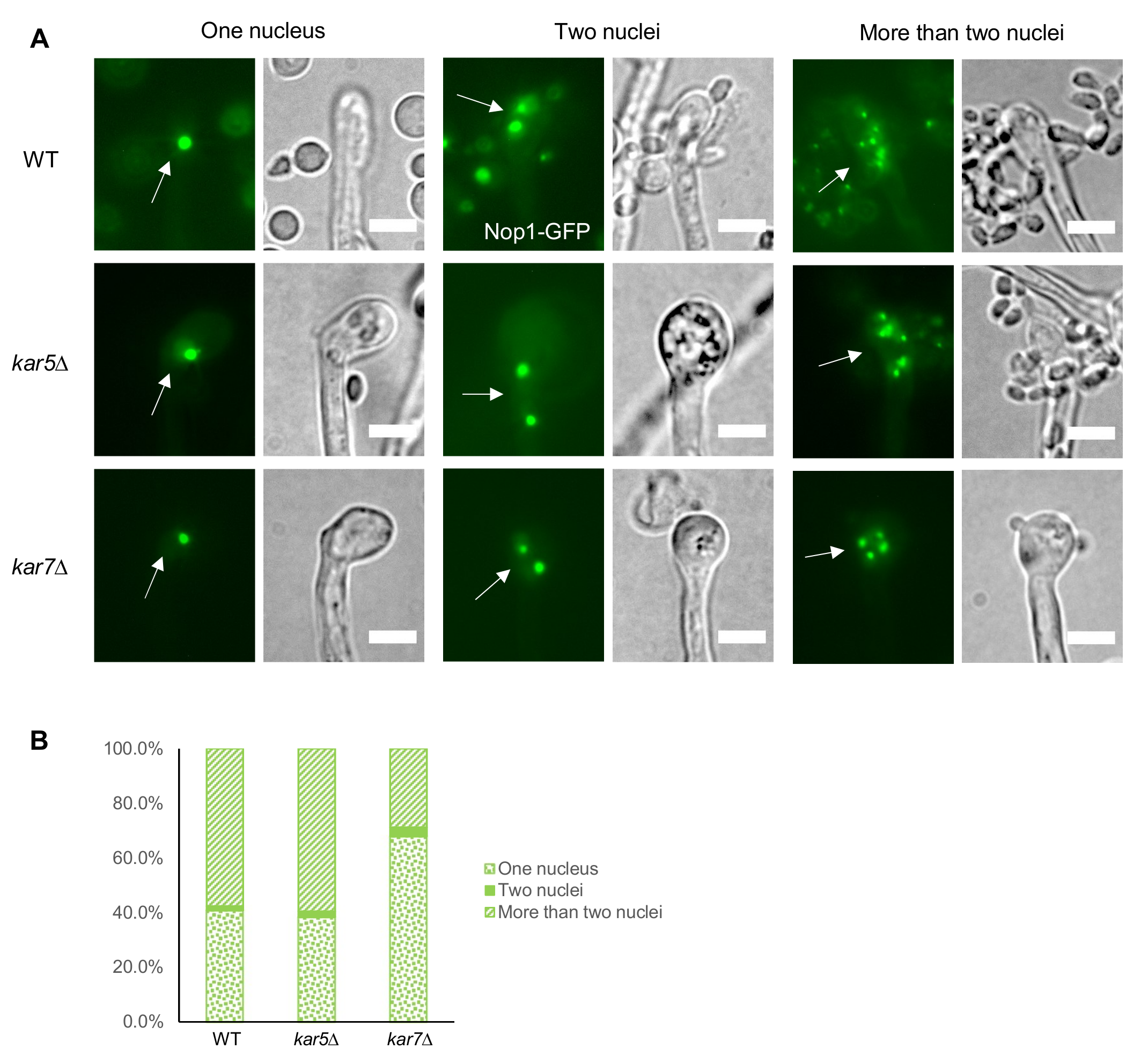

Supplement: S11 Fig — (A) Representative basidia containing one nucleus (left panel), or two nuclei (middle panel), or more than two nuclei (right panel) with the nucleolar marker Nop1-GFP fluorescent signals are shown for wild type XL280α (CF836), kar5 mutant (CF718), and kar7 mutant (CF1442). Arrows point to Nop1-GFP signal inside basidia. The scale bars are 5 μm. (B) Basidia containing one nucleus, or two nuclei, or more than two nuclei were quantified for the above strains. (TIF) [file pgen.1007113.s011.tif]

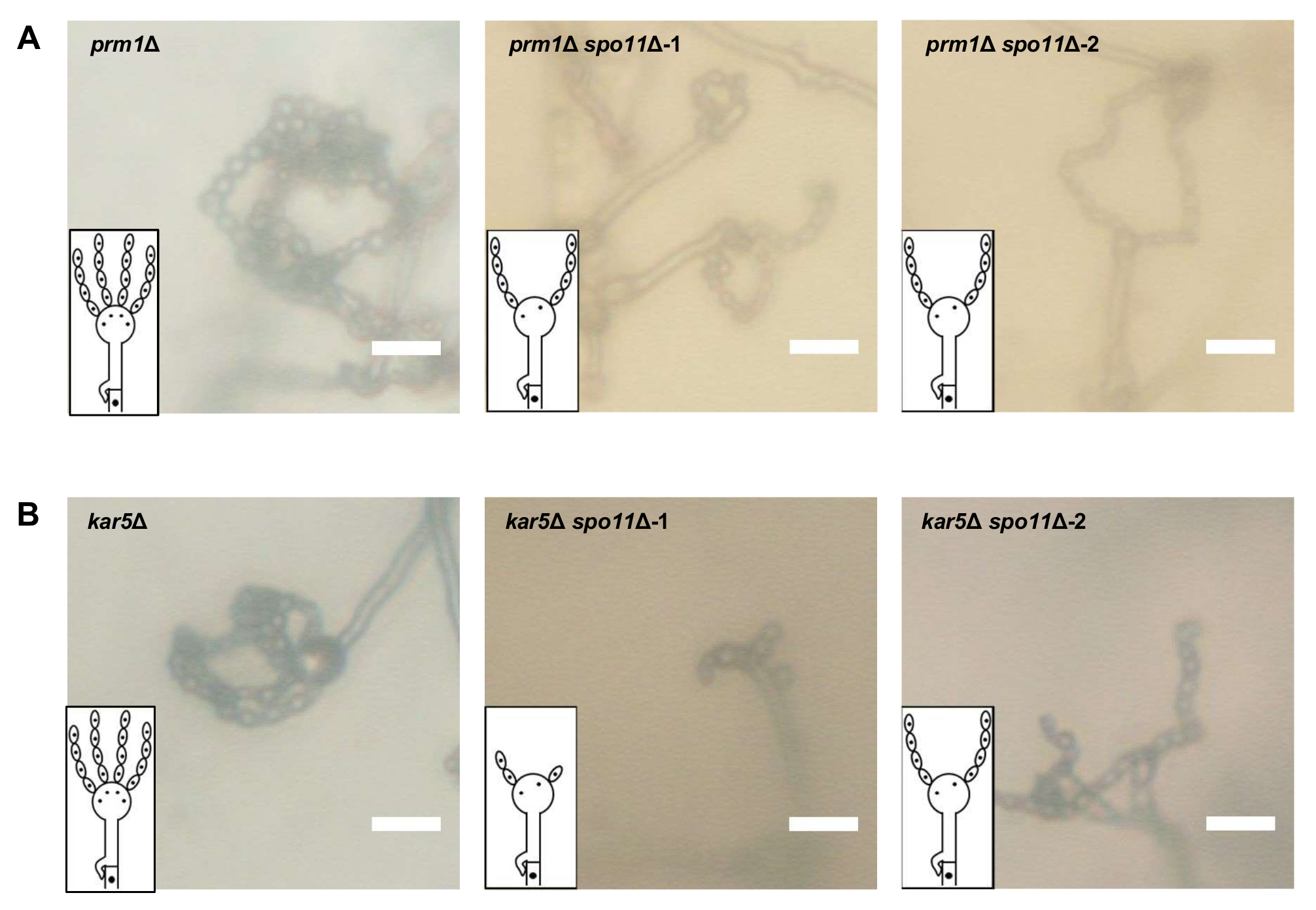

Supplement: S12 Fig — (A) prm1 mutant (CF317) and two independent prm1 spo11 double mutants (CF894 and CF901), and (B) kar5 mutant (CF260) and two independent kar5 spo11 double mutants (CF883 and CF884) were incubated on V8 medium in the dark at room temperature for four weeks. prm1 spo11 double mutants and kar5 spo11 double mutants produced two spore chains compared to the four spore chains produced by prm1 or kar5 mutants. Schemes showing wild type and mutant sporulation patterns were provided at the lower left corner of each image. The scale bar equals 10 μm. (TIF) [file pgen.1007113.s012.tif]

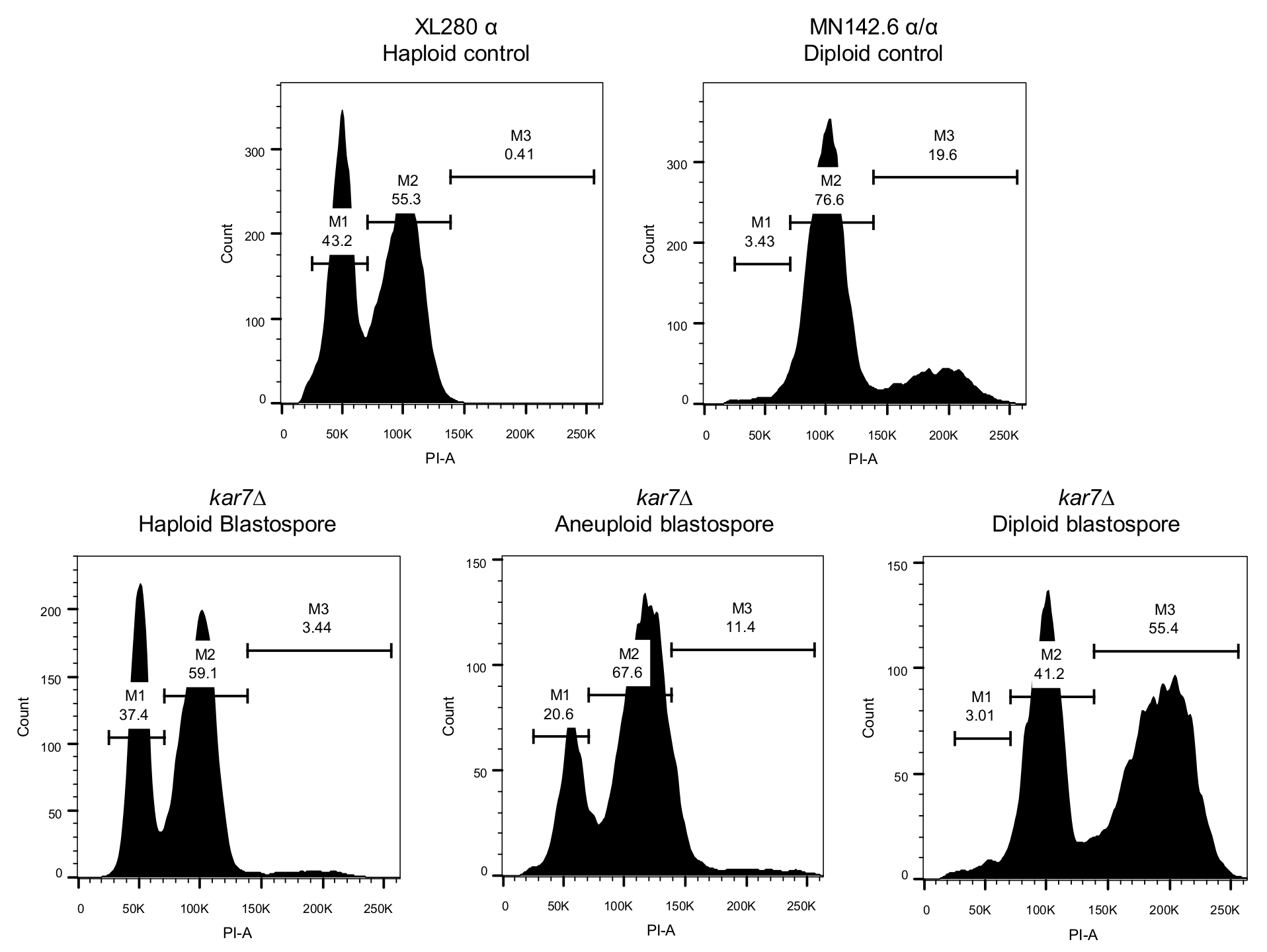

Supplement: S13 Fig — The upper panels are FACS results for haploid control XL280α and diploid control MN142.6 α/α. The lower panels are representative FACS results for haploid, aneuploid, and diploid blastospores produced by the kar7 mutant. (TIF) [file pgen.1007113.s013.tif]
