# Supplementary material for: PRM1 and KAR5 function in cell-cell fusion and karyogamy to drive distinct bisexual and unisexual cycles in the Cryptococcus pathogenic species complex
Source: PLoS Genet. 2017 Nov 27;13(11):e1007113. doi: 10.1371/journal.pgen.1007113 (PMC5720818; doi:10.1371/journal.pgen.1007113)
Supplement: S1 Table — (DOCX) [file pgen.1007113.s017.docx]

**Table S1. Strains and plasmids used in this study.**

|  | **Strain name** | **Genotype** | **Background** | **Sources and comments** |
| --- | --- | --- | --- | --- |
| ***C. deneoformans* unisexual reproduction** | XL280α | *MAT*α |  | [7] |
|  | MN142.6 | *MAT*α/α *ura5*::*NAT*/*ura5*::*NEO* | XL280α | (Ni and Heitman, unpublished data) |
|  | SL277 | *kar7*::*NEO* | XL280 | [38] |
|  | CF750 | *URA5-NAT* | XL280α | This study |
|  | CF752 | *ADE2-NEO* | XL280α | This study |
|  | CF836 | *NOP1-GFP-NAT* | XL280α | This study |
|  | CF1091 | *H3-mCherry-NAT* | XL280α | This study |
|  | CF317 | *prm1*Δ::*NEO* | XL280α | This study |
|  | CF659 | *prm1*Δ::*NAT* | XL280α | This study |
|  | CF894 | *prm1*Δ::*NEO spo11*Δ::*NAT* | XL280α | This study |
|  | CF901 | *prm1*Δ::*NEO spo11*Δ::*NAT* | XL280α | This study |
|  | CF150 | *kar5*Δ::*NAT* | XL280α | This study |
|  | CF260 | *kar5*Δ::*NEO* | XL280α | This study |
|  | CF883 | *kar5*Δ::*NEO spo11*Δ::*NAT* | XL280α | This study |
|  | CF884 | *kar5*Δ::*NEO spo11*Δ::*NAT* | XL280α | This study |
|  | CF718 | *kar5*Δ::*NEO Nop1-GFP-NAT* | XL280α | This study |
|  | CF1442 | *Kar7*Δ::*NEO Nop1-GFP-NAT* | XL280α | This study |
| ***C. deneoformans* bisexual reproduction** | JEC21α | *MAT*α |  | [55] |
|  | JEC20**a** | *MAT***a** |  | [55] |
|  | CF757 | *URA5-NAT* | JEC20**a** | This study |
|  | CF762 | *ADE2-NEO* | JEC21α | This study |
|  | CF830 | *Nop1-GFP-NAT* | JEC21α | This study |
|  | CF1076 | *H3-mCherry-NAT* | JEC20**a** | This study |
|  | CF313 | *prm1*Δ::*NAT* | JEC20**a** | This study |
|  | CF517 | *prm1*Δ::*NEO* | JEC20**a** | This study |
|  | CF1 | *prm1*Δ::*NEO* | JEC21α | This study |
|  | CF316 | *prm1*Δ::*NAT* | JEC21α | This study |
|  | CF712 | *prm1*Δ::*NAT mCherry-NEO* | JEC21α | This study |
|  | CF768 | *prm1*Δ::*NEO NOP1-GFP-NAT* | JEC20**a** | This study |
|  | CF364 | *kar5*Δ::*NAT* | JEC20**a** | This study |
|  | CF464 | *kar5*Δ::*NEO* | JEC20**a** | This study |
|  | CF226 | *kar5*Δ::*NEO* | JEC21α | This study |
|  | CF487 | *kar5*Δ::*NEO* | JEC21α | This study |
|  | CF723 | *kar5*Δ::*NEO NOP1-GFP-NAT* | JEC20**a** | This study |
|  | CF1185 | *kar5*Δ::*NEO H3-mCherry-NAT* | JEC21α | This study |

| ***C. neoformans* bisexual reproduction** | H99α |  |  | [33] |
| --- | --- | --- | --- | --- |
|  | KN99**a** |  |  | [33] |
|  | YSB119 | *aca1*Δ::*NAT ura5 ACA1-URA5* | H99α | [34] |
|  | YSB121 | *aca1*Δ::*NEO ura5 ACA1-URA5* | KN99**a** | [34] |
|  | CF30 | *prm1*Δ::*NEO* | H99α | This study |
|  | CF56 | *prm1*Δ::*NAT* | H99α | This study |
|  | CF448 | *prm1*Δ::*NAT* | KN99**a** | This study |
|  | CF562 | *prm1*Δ::*NEO* | KN99**a** | This study |
|  | CF57 | *kar5*Δ::*NAT* | H99α | This study |
|  | CF208 | *kar5*Δ::*NAT* | H99α | This study |
|  | CF305 | *kar5*Δ::*NEO* | KN99**a** | This study |
|  | CF549 | *kar5*Δ::*NEO* | KN99**a** | This study |
|  | **Plasmid** | **Genotype** | **Background** | **Sources and comments** |
|  | pAI3 | *NAT AMP* |  | [66] |
|  | pXL1 | P*_GPD1_* *NEO* *AMP* |  | (Lin and Heitman, unpublished data) |
|  | pSL04 | *Nop1-GFP NAT KAN* |  | [38] |
|  | pCF1 | P*_GPD1_-mCherry* *NEO* *AMP* | pXL1 | This study |
|  | pCF9 | P*_H3_-H3-mCherry NAT AMP* | pAI3 | This study |
