# Supplementary material for: PRM1 and KAR5 function in cell-cell fusion and karyogamy to drive distinct bisexual and unisexual cycles in the Cryptococcus pathogenic species complex
Source: PLoS Genet. 2017 Nov 27;13(11):e1007113. doi: 10.1371/journal.pgen.1007113 (PMC5720818; doi:10.1371/journal.pgen.1007113)
Supplement: S2 Table — (DOCX) [file pgen.1007113.s018.docx]

**Table S2. Primers used in this study.**

| **Primer name** | **Sequence (5’ to 3’)** | **Description** |
| --- | --- | --- |
| M13F | GTAAAACGACGGCCAGT | *NAT* and *NEO* cassette |
| M13R | CAGGAAACAGCTATGAC | *NAT* and *NEO* cassette |
| JOHE41027 | GAAACCGTTGGTAGCCAAGA | *CdPRM1* 5'UTR F |
| JOHE40968 | GCTCACTGGCCGTCGTTTTACGTCGAGAGTTGGTGGAAAGG | *CdPRM1* 5'UTR R |
| JOHE40969 | CATGGTCATAGCTGTTTCCTGTTATGAACGAGGGCTGTGAG | *CdPRM1* 3'UTR F |
| JOHE41028 | GTGCCTTATGGCTCGGCATA | *CdPRM1 3*'UTR R |
| JOHE41356 | GCTCATACCACTGCAATACAC | *CdPRM1* Junction F |
| JOHE41357 | CAGTTTAGTTGAAGCCAGGAG | *CdPRM1* Junction R |
| JOHE41025 | CGTTTCACAAGCCACAACTC | *CnPRM1* 5'UTR F |
| JOHE40946 | GCTCACTGGCCGTCGTTTTACTCGAGAGTTGGTGGAGAAGG | *CnPRM1* 5'UTR R |
| JOHE40947 | CATGGTCATAGCTGTTTCCTGGCTTTGAGCGTTGAAGATGA | *CnPRM1* 3'UTR F |
| JOHE41026 | GCGCTTACTGGAAACAGCA | *CnPRM1* 3'UTR R |
| JOHE41354 | CCGTCTTCTTCGATACCACC | *CnPRM1* Junction F |
| JOHE41355 | CTTGACTGTGTTCCTCGCAC | *CnPRM1* Junction R |
| JOHE41032 | AGATCATTGCCTGTGGATCG | *CdKAR5* 5'UTR F |
| JOHE40972 | GCTCACTGGCCGTCGTTTTACTCGGGAAGAGTAAGGACGAA | *CdKAR5* 5'UTR R |
| JOHE40973 | CATGGTCATAGCTGTTTCCTGCTGTGGAGAACCTTCGAGTC | *CdKAR5* 3'UTR F |
| JOHE41033 | CCCTCCTCTATGTCGTCTTG | *CdKAR5* 3'UTR R |
| JOHE41360 | CCCGAGGTCAAAGACAAACC | *CdKAR5* Junction F |
| JOHE41361 | GTCAGCATAGGGTCCTACCT | *CdKAR5* Junction R |
| JOHE41029 | GTGGACTGCTCTCTATTGTTTC | *CnKAR5* 5'UTR F |
| JOHE40950 | GCTCACTGGCCGTCGTTTTACTGTCCAATTGGCAGAGTGTC | *CnKAR5* 5'UTR R |
| JOHE40951 | CATGGTCATAGCTGTTTCCTGGCTGCTCTGGAGAACTTTCG | *CnKAR5* 3'UTR F |
| JOHE41030 | AGTGTCCCTCCTTCGCTTG | *CnKAR5* 3'UTR R |
| JOHE41358 | TGAAAGACTGGCGTGAGGTC | *CnKAR5* Junction F |
| JOHE41359 | TCAGCTTAGGGTCCTACCTC | *CnKAR5* Junction R |
| JOHE42818 | GTCCAAACCACTCCCTTGAT | *CdSPO11* 5'UTR F |
| JOHE42820 | ACTGGCCGTCGTTTTACGGTCATAAAGGGACGAAAGG | *CdSPO11* 5'UTR R |
| JOHE42822 | GTCATAGCTGTTTCCTGCTTCCCTTTCTGACCATCTCC | *CdSPO11* 3'UTR F |
| JOHE42823 | GGTATTGTCCGCTTGTTGAG | *CdSPO11* 3'UTR R |
| JOHE42817 | GTGAGAGAGGATAAGTCGGTTG | *CdSPO11* Junction F |
| JOHE42824 | GAACACTTGACTTGCCTCCAC | *CdSPO11* Junction R |
| JOHE42020 | CAGCGATGAGCTCATAAGCC | *CdURA5-NAT* Upstream F |
| JOHE42021 | ACTGGCCGTCGTTTTACCTCGACGAACTTCTTCGAGG | *CdURA5-NAT* Upstream R |
| JOHE42022 | CATGGTCATAGCTGTTTCCTGCTCTGATACCCGACACTCGTG | *CdURA5-NAT* Downstream F |
| JOHE42023 | CGCATCATTCGTTTGACGCC | *CdURA5-NAT* Downstream R |
| JOHE42019 | CTACTGCTACAACAGGAAGG | *CdURA5-NAT* Junction F |
| JOHE42024 | TCCCCTTCGACTTGTGTTTC | *CdURA5-NAT* Junction R |
| JOHE42014 | CTGGACCAATGCGTTAATCCC | *CdADE2-NEO* Upstream F |
| JOHE42015 | ACTGGCCGTCGTTTTACTGTGATTAGCGGATTGTC | *CdADE2-NEO* Upstream R |
| JOHE42016 | GTCATAGCTGTTTCCTGCCATCAGTCTCGCTTGATCAG | *CdADE2-NEO* Downstream F |
| JOHE42017 | CTCTTTCTCCTTCATCGTGGG | *CdADE2-NEO* Downstream R |
| JOHE42013 | GAAGACCATGTGGGAAGAAGG | *CdADE2-NEO* Junction F |
| JOHE42018 | GGATGTCCTTATGGATGCCAC | *CdADE2-NEO* Junction R |
| JOHE44120 | GTCTCCACTGATTTCATTGGCTCTAC | *CdGPD1* RTPCR F |
| JOHE44121 | GTAACCATACTCATTGTCATACCAGCTG | *CdGPD1* RTPCR R |
| JOHE42831 | TGCCTCTTCTTCCGTATCGT | *CdPRM1* RTPCR F |
| JOHE42832 | CCCCAAAGGGATCTTTTCTC | *CdPRM1* RTPCR R |
| JOHE42837 | AGCCCACACTCTCCTACTG | *CdKAR5* RTPCR F |
| JOHE42838 | TGTTCTTCGCTGCCACAT | *CdKAR5* RTPCR R |
| JOHE43005 | ATCTTCACCACCTTCACTTCT | *CdMF*α RTPCR F |
| JOHE43006 | CTAGGCGATGACACAAAGG | *CdMFα* RTPCR R |
| JOHE40392 | GTCTCTACTGATTTCGTTGGCACTAC | *CnGPD1* RTPCR F |
| JOHE40393 | GTAACCGTACTCATTGTCATACCAGCTA | *CnGPD1* RTPCR R |
| JOHE42825 | GCTGTAAGGGGGACTTTGGA | *CnPRM1* RTPCR F |
| JOHE42826 | ATGATAGTGCGGGGATGGAG | *CnPRM1* RTPCR R |
| JOHE42833 | CGAGCCCACATTATCCTTC | *CnKAR5* RTPCR F |
| JOHE42834 | TCTTCTTCGCCGCCACAT | *CnKAR5* RTPCR R |
| JOHE43007 | ATCTTCACCACCTCCATTTCT | *CnMF*α RTPCR F |
| JOHE43008 | TTAGGCGATGACGCATAGG | *CnMF*α RTPCR R |
| JOHE41446 | GCCGTGCAAGGGTGTAGG | *CdSXI1*α F |
| JOHE41447 | GGGCCATTGGAGGAAGCTG | *CdSXI1*α R |
| JOHE41444 | CGGACGAGCTCTCAAATTGG | *CdSXI2***a** F |
| JOHE41445 | TTTGCTCGCTCTCCTTCCAC | *CdSXI2***a** R |
| JOHE41442 | ACCAAAGCCCTCAGAGTCTG | *CnSXI1*α F |
| JOHE41443 | GACCGGAAACCCCAACAGTA | *CnSXI1*α R |
| JOHE41440 | CCAACAGAATGCGTCACCTC | *CnSXI2***a** F |
| JOHE41441 | TTACTCCGATCTCTGCCCAC | *CnSXI2***a** R |
| JOHE40976 | CAGGAAACAGCTATGAC | pCF1 P*_GPD1_* F |
| JOHE41658 | CTCGCCCTTGCTCACCATTGTATTTATGCAAGTATACTCC | pCF1 P*_GPD1_* R |
| JOHE41655 | ATGGTGAGCAAGGGCGAG | pCF1 *mCherry* F |
| JOHE41656 | CTACTTGTACAGCTCGTCCA | pCF1 *mCherry* R |
| JOHE41659 | TGGACGAGCTGTACAAGTAGGGCCGGCCTTAATTAAGG | pCF1 T*_GPD1_* F |
| JOHE40975 | GTAAAACGACGGCCAGT | pCF1 T*_GPD1_* R |
| JOHE41353 | CATGGTCATAGCTGTTTCCTG | pCF1 Backbone F |
| JOHE41352 | ATTCACTGGCCGTCGTTTTAC | pCF1 Backbone R |
| JOHE43323 | CAGGAAACAGCTATGACCGCGGCGTTTGCAATCTCTT | pCF9 P*_H3_-H3-mCherry* F |
| JOHE43322 | CTCGCCCTTGCTCACCATACCAGATCCACCAGACCTCTCGCCTCGGAGACG | pCF9 P*_H3_-H3-mCherry* R |
| JOHE41655 | ATGGTGAGCAAGGGCGAG | pCF9 *mCherry* F |
| JOHE41656 | CTACTTGTACAGCTCGTCCA | pCF9 *mCherry* R |
| JOHE43321 | TGGACGAGCTGTACAAGTAGGCAGCTTTTATTAGCTTGGCTTTTG | pCF9 T*_H3_* F |
| JOHE43320 | GTAAAACGACGGCCAGTTGTGTGAATGAACGAGACAGCG | pCF9 T*_H3_* R |
| JOHE43361 | GTCATAGCTGTTTCCTG | pCF9 Backbone F |
| JOHE41656 | CACACAACTGGCCGTCGTTTTACAACG | pCF9 Backbone R |

F, R: Forward and Reverse.
