# Supplementary material for: PRM1 and KAR5 function in cell-cell fusion and karyogamy to drive distinct bisexual and unisexual cycles in the Cryptococcus pathogenic species complex
Source: PLoS Genet. 2017 Nov 27;13(11):e1007113. doi: 10.1371/journal.pgen.1007113 (PMC5720818; doi:10.1371/journal.pgen.1007113)
Supplement: S3 Table — (DOCX) [file pgen.1007113.s019.docx]

**Table S3. Blastospores dissected in this study.**

| **Genotype** | **Strain description*** | **Ploidy by FACS**** |
| --- | --- | --- |
| Wild type | XL280alpha blastospore 1 66/71, 8 spots, spot 1 11/12) | Diploid |
|  | XL280alpha blastospore 2 (66/71, 8 spots, spot 1 11/12) | Diploid |
|  | XL280alpha blastospore 3 (66/71, 8 spots, spot 1 11/12) |  |
|  | XL280alpha blastospore 4 (66/71, 8 spots, spot 1 11/12) |  |
|  | XL280alpha blastospore 5 (66/71, 8 spots, spot 1 11/12) |  |
|  | XL280alpha blastospore 6 (66/71, 8 spots, spot 1 11/12) |  |
|  | XL280alpha blastospore 7 (66/71, 8 spots, spot 1 11/12) |  |
|  | XL280alpha blastospore 8 (66/71, 8 spots, spot 1 11/12) |  |
|  | XL280alpha blastospore 9 (66/71, 8 spots, spot 1 11/12) |  |
|  | XL280alpha blastospore 10 (66/71, 8 spots, spot 1 11/12) |  |
|  | XL280alpha blastospore 11 (66/71, 8 spots, spot 1 11/12) |  |
|  | XL280alpha blastospore 12 (66/71, 8 spots, spot 2 10/10) | Diploid |
|  | XL280alpha blastospore 13 (66/71, 8 spots, spot 2 10/10) | Diploid |
|  | XL280alpha blastospore 14 (66/71, 8 spots, spot 2 10/10) |  |
|  | XL280alpha blastospore 15 (66/71, 8 spots, spot 2 10/10) |  |
|  | XL280alpha blastospore 16 (66/71, 8 spots, spot 2 10/10) |  |
|  | XL280alpha blastospore 17 (66/71, 8 spots, spot 2 10/10) |  |
|  | XL280alpha blastospore 18 (66/71, 8 spots, spot 2 10/10) |  |
|  | XL280alpha blastospore 19 (66/71, 8 spots, spot 2 10/10) |  |
|  | XL280alpha blastospore 20 (66/71, 8 spots, spot 2 10/10) |  |
|  | XL280alpha blastospore 21 (66/71, 8 spots, spot 2 10/10) |  |
|  | XL280alpha blastospore 22 (66/71, 8 spots, spot 3 8/8) | Diploid |
|  | XL280alpha blastospore 23 (66/71, 8 spots, spot 3 8/8) | Diploid |
|  | XL280alpha blastospore 24 (66/71, 8 spots, spot 3 8/8) |  |
|  | XL280alpha blastospore 25 (66/71, 8 spots, spot 3 8/8) |  |
|  | XL280alpha blastospore 26 (66/71, 8 spots, spot 3 8/8) |  |
|  | XL280alpha blastospore 27 (66/71, 8 spots, spot 3 8/8) |  |
|  | XL280alpha blastospore 28 (66/71, 8 spots, spot 3 8/8) |  |
|  | XL280alpha blastospore 29 (66/71, 8 spots, spot 3 8/8) |  |
|  | XL280alpha blastospore 30 (66/71, 8 spots, spot 4 11/11) | Diploid |
|  | XL280alpha blastospore 31 (66/71, 8 spots, spot 4 11/11) | Diploid |
|  | XL280alpha blastospore 32 (66/71, 8 spots, spot 4 11/11) |  |
|  | XL280alpha blastospore 33 (66/71, 8 spots, spot 4 11/11) |  |
|  | XL280alpha blastospore 34 (66/71, 8 spots, spot 4 11/11) |  |
|  | XL280alpha blastospore 35 (66/71, 8 spots, spot 4 11/11) |  |
|  | XL280alpha blastospore 36 (66/71, 8 spots, spot 4 11/11) |  |
|  | XL280alpha blastospore 37 (66/71, 8 spots, spot 4 11/11) |  |
|  | XL280alpha blastospore 38 (66/71, 8 spots, spot 4 11/11) |  |
|  | XL280alpha blastospore 39 (66/71, 8 spots, spot 4 11/11) |  |
|  | XL280alpha blastospore 40 (66/71, 8 spots, spot 4 11/11) |  |
|  | XL280alpha blastospore 41 (66/71, 8 spots, spot 5 7/8) | Diploid |
|  | XL280alpha blastospore 42 (66/71, 8 spots, spot 5 7/8) | Diploid |
|  | XL280alpha blastospore 43 (66/71, 8 spots, spot 5 7/8) |  |
|  | XL280alpha blastospore 44 (66/71, 8 spots, spot 5 7/8) |  |
|  | XL280alpha blastospore 45 (66/71, 8 spots, spot 5 7/8) |  |
|  | XL280alpha blastospore 46 (66/71, 8 spots, spot 5 7/8) |  |
|  | XL280alpha blastospore 47 (66/71, 8 spots, spot 5 7/8) |  |
|  | XL280alpha blastospore 48 (66/71, 8 spots, spot 6 8/9) | Diploid |
|  | XL280alpha blastospore 49 (66/71, 8 spots, spot 6 8/9) | Diploid |
|  | XL280alpha blastospore 50 (66/71, 8 spots, spot 6 8/9) |  |
|  | XL280alpha blastospore 51 (66/71, 8 spots, spot 6 8/9) |  |
|  | XL280alpha blastospore 52 (66/71, 8 spots, spot 6 8/9) |  |
|  | XL280alpha blastospore 53 (66/71, 8 spots, spot 6 8/9) |  |
|  | XL280alpha blastospore 54 (66/71, 8 spots, spot 6 8/9) |  |
|  | XL280alpha blastospore 55 (66/71, 8 spots, spot 6 8/9) |  |
|  | XL280alpha blastospore 56 (66/71, 8 spots, spot 7 6/6) | Diploid |
|  | XL280alpha blastospore 57 (66/71, 8 spots, spot 7 6/6) | Diploid |
|  | XL280alpha blastospore 58 (66/71, 8 spots, spot 7 6/6) |  |
|  | XL280alpha blastospore 59 (66/71, 8 spots, spot 7 6/6) |  |
|  | XL280alpha blastospore 60 (66/71, 8 spots, spot 7 6/6) |  |
|  | XL280alpha blastospore 61 (66/71, 8 spots, spot 7 6/6) |  |
|  | XL280alpha blastospore 62 (66/71, 8 spots, spot 8 5/7) | Diploid |
|  | XL280alpha blastospore 63 (66/71, 8 spots, spot 8 5/7) | Diploid |
|  | XL280alpha blastospore 64 (66/71, 8 spots, spot 8 5/7) |  |
|  | XL280alpha blastospore 65 (66/71, 8 spots, spot 8 5/7) |  |
|  | XL280alpha blastospore 66 (66/71, 8 spots, spot 8 5/7) |  |
| *prm1*∆ | CF659 blastospore 1 (10/52, 8 spots, spot1 1/3) 2-0/8, 5-0/7, 8-0/7 | Diploid |
|  | CF659 blastospore 2 (10/52, 8 spots, spot3 1/6) 2-0/8, 5-0/7, 8-0/7 | Diploid |
|  | CF659 blastospore 3 (10/52, 8 spots, spot4 4/6) 2-0/8, 5-0/7, 8-0/7 | Diploid |
|  | CF659 blastospore 4 (10/52, 8 spots, spot4 4/6) 2-0/8, 5-0/7, 8-0/7 | Diploid |
|  | CF659 blastospore 5 (10/52, 8 spots, spot4 4/6) 2-0/8, 5-0/7, 8-0/7 | Diploid |
|  | CF659 blastospore 6 (10/52, 8 spots, spot4 4/6) 2-0/8, 5-0/7, 8-0/7 | Diploid |
|  | CF659 blastospore 7 (10/52, 8 spots, spot6 2/6) 2-0/8, 5-0/7, 8-0/7 | Diploid |
|  | CF659 blastospore 8 (10/52, 8 spots, spot6 2/6) 2-0/8, 5-0/7, 8-0/7 | Diploid |
|  | CF659 blastospore 9 (10/52, 8 spots, spot7 2/9) 2-0/8, 5-0/7, 8-0/7 | Diploid |
|  | CF659 blastospore 10 (10/52, 8 spots, spot7 2/9) 2-0/8, 5-0/7, 8-0/7 | Diploid |
| *prm1*∆ *spo11*∆ | CF894 blastospore 1 (38/60, 8 spots, spot 2 2/5) 1-0/7, 3-0/4 | Diploid |
|  | CF894 blastospore 2 (38/60, 8 spots, spot 2 2/5) 1-0/7, 3-0/4 | Diploid |
|  | CF894 blastospore 3 (38/60, 8 spots, spot 4 7/7) 1-0/7, 3-0/4 | Diploid |
|  | CF894 blastospore 4 (38/60, 8 spots, spot 4 7/7) 1-0/7, 3-0/4 | Diploid |
|  | CF894 blastospore 5 (38/60, 8 spots, spot 4 7/7) 1-0/7, 3-0/4 |  |
|  | CF894 blastospore 6 (38/60, 8 spots, spot 4 7/7) 1-0/7, 3-0/4 |  |
|  | CF894 blastospore 7 (38/60, 8 spots, spot 4 7/7) 1-0/7, 3-0/4 |  |
|  | CF894 blastospore 8 (38/60, 8 spots, spot 4 7/7) 1-0/7, 3-0/4 |  |
|  | CF894 blastospore 9 (38/60, 8 spots, spot 4 7/7) 1-0/7, 3-0/4 |  |
|  | CF894 blastospore 10 (38/60, 8 spots, spot 5 6/7) 1-0/7, 3-0/4 | Diploid |
|  | CF894 blastospore 11 (38/60, 8 spots, spot 5 6/7) 1-0/7, 3-0/4 | Diploid |
|  | CF894 blastospore 12 (38/60, 8 spots, spot 5 6/7) 1-0/7, 3-0/4 |  |
|  | CF894 blastospore 13 (38/60, 8 spots, spot 5 6/7) 1-0/7, 3-0/4 |  |
|  | CF894 blastospore 14 (38/60, 8 spots, spot 5 6/7) 1-0/7, 3-0/4 |  |
|  | CF894 blastospore 15 (38/60, 8 spots, spot 5 6/7) 1-0/7, 3-0/4 |  |
|  | CF894 blastospore 16 (38/60, 8 spots, spot 6 12/12) 1-0/7, 3-0/4 | Diploid |
|  | CF894 blastospore 17 (38/60, 8 spots, spot 6 12/12) 1-0/7, 3-0/4 | Diploid |
|  | CF894 blastospore 18 (38/60, 8 spots, spot 6 12/12) 1-0/7, 3-0/4 |  |
|  | CF894 blastospore 19 (38/60, 8 spots, spot 6 12/12) 1-0/7, 3-0/4 |  |
|  | CF894 blastospore 20 (38/60, 8 spots, spot 6 12/12) 1-0/7, 3-0/4 |  |
|  | CF894 blastospore 21 (38/60, 8 spots, spot 6 12/12) 1-0/7, 3-0/4 |  |
|  | CF894 blastospore 22 (38/60, 8 spots, spot 6 12/12) 1-0/7, 3-0/4 |  |
|  | CF894 blastospore 23 (38/60, 8 spots, spot 6 12/12) 1-0/7, 3-0/4 |  |
|  | CF894 blastospore 24 (38/60, 8 spots, spot 6 12/12) 1-0/7, 3-0/4 |  |
|  | CF894 blastospore 25 (38/60, 8 spots, spot 6 12/12) 1-0/7, 3-0/4 |  |
|  | CF894 blastospore 26 (38/60, 8 spots, spot 6 12/12) 1-0/7, 3-0/4 |  |
|  | CF894 blastospore 27 (38/60, 8 spots, spot 6 12/12) 1-0/7, 3-0/4 |  |
|  | CF894 blastospore 28 (38/60, 8 spots, spot 7 3/10) 1-0/7, 3-0/4 | Diploid |
|  | CF894 blastospore 29 (38/60, 8 spots, spot 7 3/10) 1-0/7, 3-0/4 | Diploid |
|  | CF894 blastospore 30 (38/60, 8 spots, spot 7 3/10) 1-0/7, 3-0/4 |  |
|  | CF894 blastospore 31 (38/60, 8 spots, spot 8 8/8) 1-0/7, 3-0/4 | Diploid |
|  | CF894 blastospore 32 (38/60, 8 spots, spot 8 8/8) 1-0/7, 3-0/4 | Diploid |
|  | CF894 blastospore 33 (38/60, 8 spots, spot 8 8/8) 1-0/7, 3-0/4 |  |
|  | CF894 blastospore 34 (38/60, 8 spots, spot 8 8/8) 1-0/7, 3-0/4 |  |
|  | CF894 blastospore 35 (38/60, 8 spots, spot 8 8/8) 1-0/7, 3-0/4 |  |
|  | CF894 blastospore 36 (38/60, 8 spots, spot 8 8/8) 1-0/7, 3-0/4 |  |
|  | CF894 blastospore 37 (38/60, 8 spots, spot 8 8/8) 1-0/7, 3-0/4 |  |
|  | CF894 blastospore 38 (38/60, 8 spots, spot 8 8/8) 1-0/7, 3-0/4 |  |
| *kar5*∆ | CF260 blastospore 1 (36/58, 8 spots, spot 2 7/9) 1-0/10 | Diploid |
|  | CF260 blastospore 2 (36/58, 8 spots, spot 2 7/9) 1-0/10 | Diploid |
|  | CF260 blastospore 3 (36/58, 8 spots, spot 2 7/9) 1-0/10 |  |
|  | CF260 blastospore 4 (36/58, 8 spots, spot 2 7/9) 1-0/10 |  |
|  | CF260 blastospore 5 (36/58, 8 spots, spot 2 7/9) 1-0/10 |  |
|  | CF260 blastospore 6 (36/58, 8 spots, spot 2 7/9) 1-0/10 |  |
|  | CF260 blastospore 7 (36/58, 8 spots, spot 2 7/9) 1-0/10 |  |
|  | CF260 blastospore 8 (36/58, 8 spots, spot 3 4/5) 1-0/10 | Diploid |
|  | CF260 blastospore 9 (36/58, 8 spots, spot 3 4/5) 1-0/10 | Diploid |
|  | CF260 blastospore 10 (36/58, 8 spots, spot 3 4/5) 1-0/10 |  |
|  | CF260 blastospore 11 (36/58, 8 spots, spot 3 4/5) 1-0/10 |  |
|  | CF260 blastospore 12 (36/58, 8 spots, spot 4 4/8) 1-0/10 | Diploid |
|  | CF260 blastospore 13 (36/58, 8 spots, spot 4 4/8) 1-0/10 | Diploid |
|  | CF260 blastospore 14 (36/58, 8 spots, spot 4 4/8) 1-0/10 |  |
|  | CF260 blastospore 15 (36/58, 8 spots, spot 4 4/8) 1-0/10 |  |
|  | CF260 blastospore 16 (36/58, 8 spots, spot 5 2/4) 1-0/10 | Diploid |
|  | CF260 blastospore 17 (36/58, 8 spots, spot 5 2/4) 1-0/10 | Diploid |
|  | CF260 blastospore 18 (36/58, 8 spots, spot 6 5/8) 1-0/10 | Diploid |
|  | CF260 blastospore 19 (36/58, 8 spots, spot 6 5/8) 1-0/10 | Diploid |
|  | CF260 blastospore 20 (36/58, 8 spots, spot 6 5/8) 1-0/10 |  |
|  | CF260 blastospore 21 (36/58, 8 spots, spot 6 5/8) 1-0/10 |  |
|  | CF260 blastospore 22 (36/58, 8 spots, spot 6 5/8) 1-0/10 |  |
|  | CF260 blastospore 23 (36/58, 8 spots, spot 7 7/7) 1-0/10 | Diploid |
|  | CF260 blastospore 24 (36/58, 8 spots, spot 7 7/7) 1-0/10 | Diploid |
|  | CF260 blastospore 25 (36/58, 8 spots, spot 7 7/7) 1-0/10 |  |
|  | CF260 blastospore 26 (36/58, 8 spots, spot 7 7/7) 1-0/10 |  |
|  | CF260 blastospore 27 (36/58, 8 spots, spot 7 7/7) 1-0/10 |  |
|  | CF260 blastospore 28 (36/58, 8 spots, spot 7 7/7) 1-0/10 |  |
|  | CF260 blastospore 29 (36/58, 8 spots, spot 7 7/7) 1-0/10 |  |
|  | CF260 blastospore 30 (36/58, 8 spots, spot 8 7/7) 1-0/10 | Diploid |
|  | CF260 blastospore 31 (36/58, 8 spots, spot 8 7/7) 1-0/10 | Diploid |
|  | CF260 blastospore 32 (36/58, 8 spots, spot 8 7/7) 1-0/10 |  |
|  | CF260 blastospore 33 (36/58, 8 spots, spot 8 7/7) 1-0/10 |  |
|  | CF260 blastospore 34 (36/58, 8 spots, spot 8 7/7) 1-0/10 |  |
|  | CF260 blastospore 35 (36/58, 8 spots, spot 8 7/7) 1-0/10 |  |
|  | CF260 blastospore 36 (36/58, 8 spots, spot 8 7/7) 1-0/10 |  |
| *kar5*∆ *spo11*∆ | CF884 blastospore 52 (37/40, 10 spots, spot 1, 3/3) | Diploid |
|  | CF884 blastospore 53 (37/40, 10 spots, spot 1, 3/3) | Diploid |
|  | CF884 blastospore 54 (37/40, 10 spots, spot 1, 3/3) |  |
|  | CF884 blastospore 55 (37/40, 10 spots, spot 2, 3/3) | Diploid |
|  | CF884 blastospore 56 (37/40, 10 spots, spot 2, 3/3) | Diploid |
|  | CF884 blastospore 57 (37/40, 10 spots, spot 2, 3/3) |  |
|  | CF884 blastospore 58 (37/40, 10 spots, spot 3, 5/5) | Diploid |
|  | CF884 blastospore 59 (37/40, 10 spots, spot 3, 5/5) | Diploid |
|  | CF884 blastospore 60 (37/40, 10 spots, spot 3, 5/5) |  |
|  | CF884 blastospore 61 (37/40, 10 spots, spot 3, 5/5) |  |
|  | CF884 blastospore 62 (37/40, 10 spots, spot 3, 5/5) |  |
|  | CF884 blastospore 63 (37/40, 10 spots, spot 4, 4/4) | Diploid |
|  | CF884 blastospore 64 (37/40, 10 spots, spot 4, 4/4) | Diploid |
|  | CF884 blastospore 65 (37/40, 10 spots, spot 4, 4/4) |  |
|  | CF884 blastospore 66 (37/40, 10 spots, spot 4, 4/4) |  |
|  | CF884 blastospore 67 (37/40, 10 spots, spot 5, 4/4) | Haploid |
|  | CF884 blastospore 68 (37/40, 10 spots, spot 5, 4/4) | Haploid |
|  | CF884 blastospore 69 (37/40, 10 spots, spot 5, 4/4) |  |
|  | CF884 blastospore 70 (37/40, 10 spots, spot 5, 4/4) |  |
|  | CF884 blastospore 71 (37/40, 10 spots, spot 6, 2/2) | Diploid |
|  | CF884 blastospore 72 (37/40, 10 spots, spot 6, 2/2) | Diploid |
|  | CF884 blastospore 73 (37/40, 10 spots, spot 7, 7/7) | Haploid |
|  | CF884 blastospore 74 (37/40, 10 spots, spot 7, 7/7) | Haploid |
|  | CF884 blastospore 75 (37/40, 10 spots, spot 7, 7/7) |  |
|  | CF884 blastospore 76 (37/40, 10 spots, spot 7, 7/7) |  |
|  | CF884 blastospore 77 (37/40, 10 spots, spot 7, 7/7) |  |
|  | CF884 blastospore 78 (37/40, 10 spots, spot 7, 7/7) |  |
|  | CF884 blastospore 79 (37/40, 10 spots, spot 7, 7/7) |  |
|  | CF884 blastospore 80 (37/40, 10 spots, spot 8, 5/5) | Haploid |
|  | CF884 blastospore 81 (37/40, 10 spots, spot 8, 5/5) | Haploid |
|  | CF884 blastospore 82 (37/40, 10 spots, spot 8, 5/5) |  |
|  | CF884 blastospore 83 (37/40, 10 spots, spot 8, 5/5) |  |
|  | CF884 blastospore 84 (37/40, 10 spots, spot 8, 5/5) |  |
|  | CF884 blastospore 85 (37/40, 10 spots, spot 9, 1/3) | Diploid |
|  | CF884 blastospore 86 (37/40, 10 spots, spot 10, 3/4) | Diploid |
|  | CF884 blastospore 87 (37/40, 10 spots, spot 10, 3/4) | Diploid |
|  | CF884 blastospore 88 (37/40, 10 spots, spot 10, 3/4) |  |
| *Kar7*∆ | SL277 blastospore 1 (16/248, 46 spots, spot 8, 1/1) | Diploid |
|  | SL277 blastospore 2 (16/248, 46 spots, spot 15, 2/5) | Diploid |
|  | SL277 blastospore 3 (16/248, 46 spots, spot 15, 2/5) | Diploid |
|  | SL277 blastospore 4 (16/248, 46 spots, spot 17, 1/2) | Diploid |
|  | SL277 blastospore 5 (16/248, 46 spots, spot 21, 2/4) | Haploid |
|  | SL277 blastospore 6 (16/248, 46 spots, spot 21, 2/4) | Haploid |
|  | SL277 blastospore 7 (16/248, 46 spots, spot 22, 2/5) | Aneuploid |
|  | SL277 blastospore 8 (16/248, 46 spots, spot 22, 2/5) | Haploid |
|  | SL277 blastospore 9 (16/248, 46 spots, spot 24, 2/9) | Diploid |
|  | SL277 blastospore 10 (16/248, 46 spots, spot 24, 2/9) | Diploid |
|  | SL277 blastospore 11 (16/248, 46 spots, spot 30, 1/9) | Diploid |
|  | SL277 blastospore 12 (16/248, 46 spots, spot 38, 3/10) | Aneuploid |
|  | SL277 blastospore 13 (16/248, 46 spots, spot 38, 3/10) | Aneuploid |
|  | SL277 blastospore 14 (16/248, 46 spots, spot 38, 3/10) |  |
|  | SL277 blastospore 15 (16/248, 46 spots, spot 41, 1/3) | Diploid |
|  | SL277 blastospore 16 (16/248, 46 spots, spot 43, 1/5) | Diploid |

* Blastospore information (survival of all blastospores dissected for the given strain, total number of spots, the spot that the blastospore is derived from, survival of blastospores dissected from this spot) budding sites without spore germination.

** For each budding site, no more than two blastospores were chosen for FACS determination of ploidy.
